# Synthesis of Azidodifluoromethyl Phenyl Sulfone and Its Use as a Synthetic Equivalent of the Azidodifluoromethyl Anion

**DOI:** 10.1021/acs.joc.3c00256

**Published:** 2023-05-18

**Authors:** Mykyta Ziabko, Blanka Klepetářová, Petr Beier

**Affiliations:** † Institute of Organic Chemistry and Biochemistry of the Czech Academy of Sciences, Flemingovo Náměstí, 2, 166 10 Prague 6, Czech Republic; ‡ Department of Organic Chemistry, Faculty of Science, Charles University, Hlavova 2030/8, 128 43 Prague, Czech Republic

## Abstract

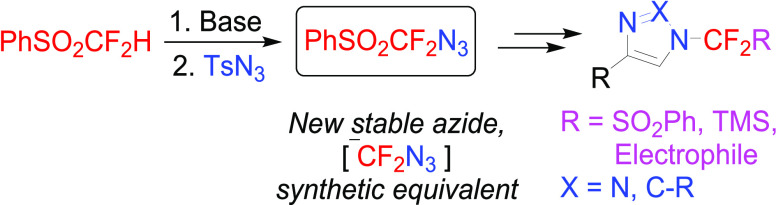

Azidodifluoromethyl phenyl sulfone, a new stable fluorinated azide, was synthesized on a multi-gram scale from difluoromethyl phenyl sulfone. The synthetic utility of the title azide in the preparation of *N*-difluoro(phenylsulfonyl)methyl-1,2,3-triazoles was demonstrated on examples of azide–alkyne cycloaddition reactions. Subsequent reductive desulfonylation/silylation afforded *N*-difluoro(trimethylsilyl)methyl-1,2,3-triazoles, and rhodium(II)-catalyzed transannulation with nitriles provided *N*-difluoro(phenylsulfonyl)methyl-substituted imidazoles. The title azide thus represents a synthetic equivalent of the azidodifluoromethyl anion.

## Introduction

Organic azides are highly valuable compounds in synthesis.^1,2^ Their utility, however, extends outside this realm, especially since the development of copper-catalyzed^3,4^ and strain-promoted azide–alkyne cycloaddition reactions.^5−7^ Nowadays, organic azides are widely used in bioconjugation, drug discovery, pharmacology, and also in materials science.^8−14^

Fluorinated analogues of organic azides, especially α-fluorinated azidoalkanes, were very rare until 2017, when we reported the synthesis of azidoperfluoroalkanes by the reaction of fluorinated carbanion precursors and an electrophilic azide source.^15^ Since then, many new fluorinated azides have been prepared and their stability and reactivity investigated.^16,17^ They serve as versatile intermediates in the synthesis of new nitrogen heterocycles and N-alkenyl compounds.^18−20^

The following one-carbon fluorinated azidoalkanes, azidotrifluoromethane (CF_3_N_3_), azidodifluoromethane (HCF_2_N_3_), and azidofluoromethane (FCH_2_N_3_), were reported. However, simple halogenated or silylated difluoromethyl azides XCF_2_N_3_ (X = Cl, Br, I, TMS), which might potentially serve as azidodifluoromethyl carbanion or radical precursors, are unknown. Our experience with azidofluoroalkanes suggests that this is caused by a lack of suitable methods for their synthesis rather than low product stability. We therefore set out to attempt the synthesis of new difluoromethylated azides which could serve as azidodifluoromethyl carbanion precursors for the synthesis of previously unknown N-CF_2_X-substituted nitrogen heterocycles by [3 + 2] cycloaddition with alkynes and follow-up chemistry.

## Results and Discussion

**Scheme 1:**
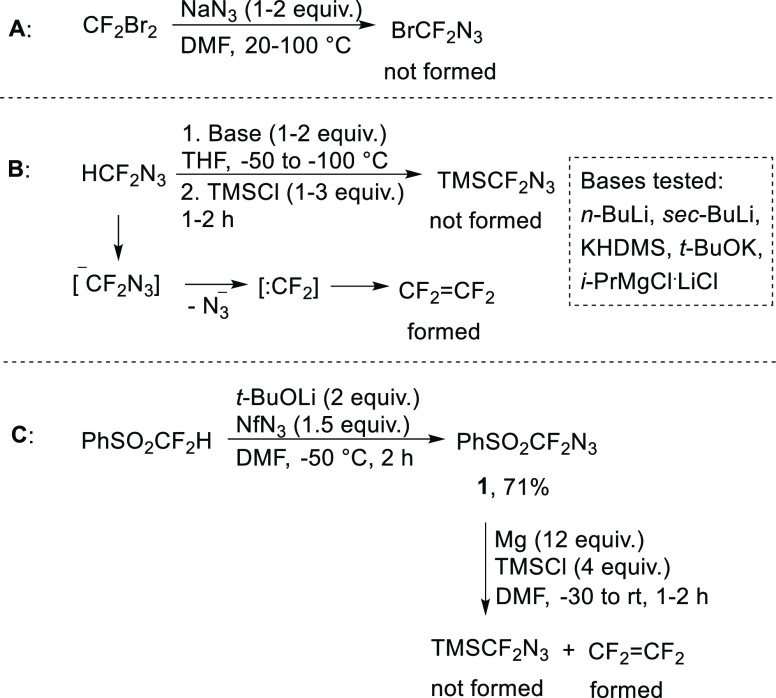
Synthetic Approaches to XCF_2_N_3_

For the synthesis of azidobromodifluoromethane (BrCF_2_N_3_), we attempted to substitute the bromine atom of CF_2_Br_2_ with sodium azide, however, without success (Scheme 1A). TMSCF_2_N_3_ might be accessible by the deprotonation of azidodifluoromethane with a suitable base, followed by silylation with trimethylsilyl chloride. This approach, however, led only to the elimination of the azide-leaving group and the formation of tetrafluoroethylene by dimerization of difluorocarbene (Scheme 1B). The other approach to the synthesis of TMSCF_2_N_3_ was based on the use of a masked phenylsulfonyl group, from which the silyl group would be formed by a reductive desulfonylation/silylation process.^21,22^ Deprotonation of difluoromethyl phenyl sulfone with *t*-BuOLi and a reaction with nonaflyl azide (NfN_3_) smoothly produced the new azide **1** in good isolated yield (Scheme 1C). Subsequent silylation in the presence of magnesium metal was again unsuccessful and gave tetrafluoroethylene decomposition side products. These initial investigations point to a low stability of the azidodifluoromethyl anion; its decomposition proceeds by azide anion elimination. However, sulfone **1** can act as a synthetic equivalent of the azidodifluoromethyl anion, as demonstrated herein below.

Optimization of the synthesis of azide **1** was performed. The reaction proceeded well at low temperatures with an excess of *t*-BuOLi or *t*-BuOK, with nonaflyl azide (NfN_3_), or with the more readily available tosyl azide (TsN_3_) (Table 1). The highest isolated yield of **1** was achieved using excess *t*-BuOK and an equimolar amount of TsN_3_ at low temperature (entry 7). Scaling up to 6 grams of **1** was straightforward.

**Table 1:**
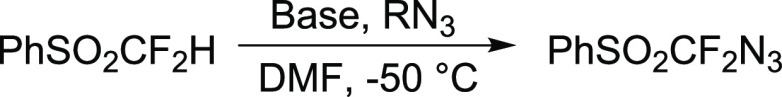
Optimization of Sulfone 1 Preparation

entry	base (equiv)	RN_3_ (equiv)	time (h)	yield of **1** (%)^a^
1	*t*-BuOLi (2.0)	NfN_3_ (1.5)	1.0	80 (78)
2^b^	*t*-BuOLi (2.5)	NfN_3_ (2.0)	1.5	64 (52)
3^b^	*t*-BuOLi (3.0)	NfN_3_ (2.0)	1.5	51 (40)
4	*t*-BuOLi (4.5)	NfN_3_ (3.0)	2.0	90 (84)
5	*t*-BuOK (3.0)	TsN_3_ (1.0)	2.0	80
6	*t*-BuOK (4.0)	TsN_3_ (1.0)	2.0	90 (80)
7^c^	*t*-BuOK (5.0)	TsN_3_ (1.0)	2.0	>97 (90)

a ^19^F NMR yield; in parentheses, isolated yield.

b Reaction temperature: −50 °C to rt.

c Reaction performed on a 6 g scale. Nf = *n*-C_4_F_9_SO_2_, Ts = *p*-TolSO_2_.

Thermal stability is an important characteristic of each new organic azide. The stability of azide **1** was determined by heating CDCl_3_ or DMSO-*d*_6_ solutions in a thick wall, sealable NMR tube and analysis by ^1^H and ^19^F NMR and by differential scan calorimetry (DSC) and thermogravimetric (TG) analyses. No signs of decomposition were observed when the sample was heated to 100 °C for 1 h. Trace amounts of decomposition products were observed by heating the DMSO-*d*_6_ solution to 140 °C for 1 h, and complete decomposition was observed upon heating the sample to 180 °C for 1 h. DSC and TG analysis confirmed these observations (onset of decomposition at 130–140 °C and exotherm maximum at 176 °C). Fall-hammer test established the insensitivity of the compound to the impact of energy below 50 J. We therefore concluded that azide **1** is safe to use on a laboratory scale at ambient temperature.

With azide **1** in hand, copper(I)-catalyzed azide–alkyne cycloaddition was performed under conditions previously employed for the click reaction with other known fluorinated azides^15^ (Table 2). With the use of a slight excess of various aryl, heteroaryl, alkyl, cycloalkyl, cycloalkenyl, and substituted aryl and alkyl acetylenes, a catalytic amount of copper(I) methylsalicylate in THF under mild conditions, a variety of *N*-difluoro(phenylsulfonyl)methyl-1,2,3-triazoles (**2**) were prepared in good to excellent yields (Table 2). Ether, hydroxyl, ester, and halogen groups, as well as unsaturation, are all compatible with the reaction. A double click reaction was performed, and bis(triazole) **2p** was isolated in high yield. In addition, triazole **2a** was prepared on a scale of 6.3 g.

**Table 2:**
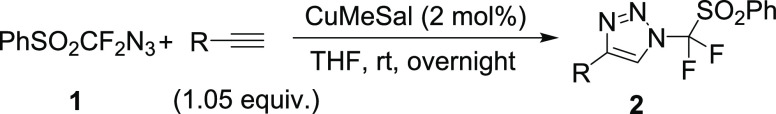

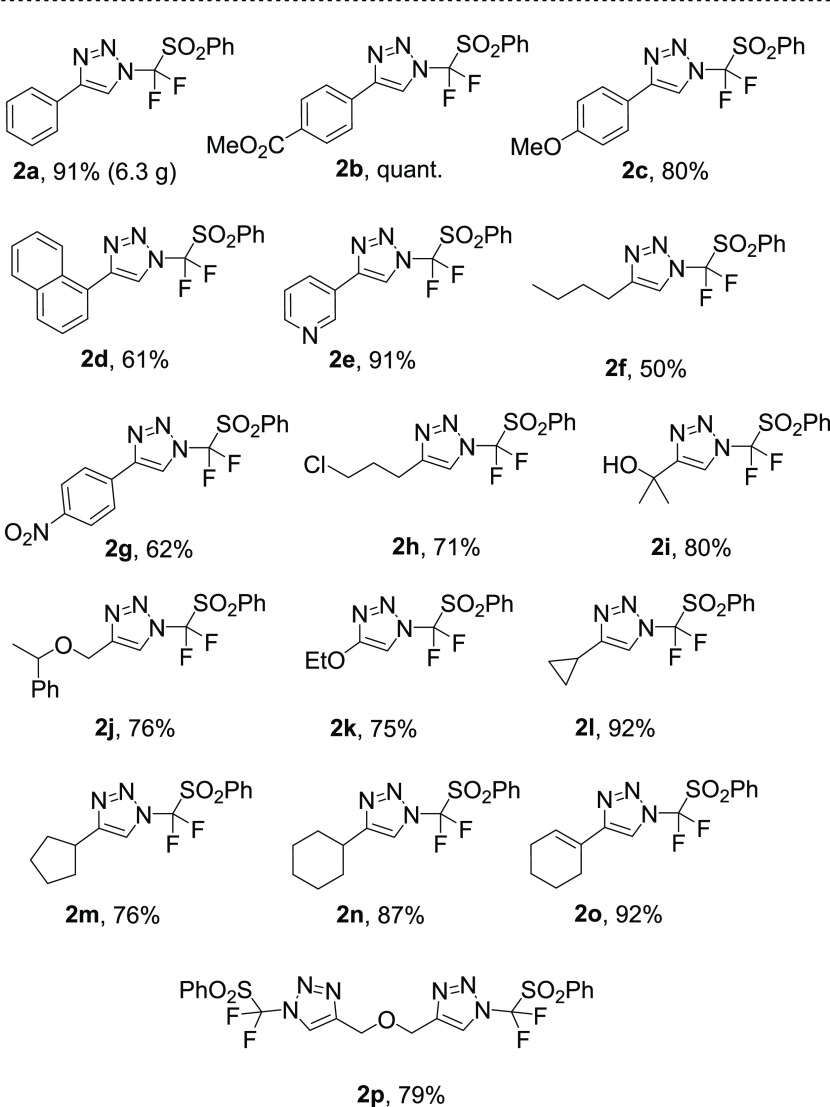
Synthesis of *N*-Difluoro(phenylsulfonyl)methyl-1,2,3-triazoles (**2**) by Cycloaddition Reactions

**Scheme 2:**
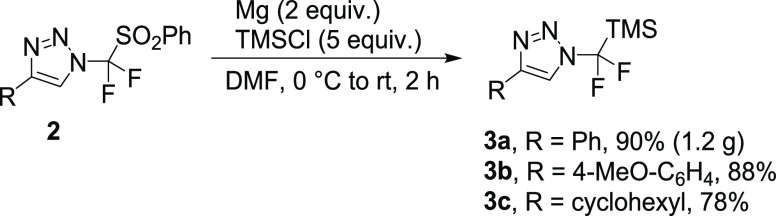
Synthesis of *N*-Difluoro(trimethylsilyl)methyl-1,2,3-triazoles (**3**)

Whereas reductive desulfonylation/silylation did not proceed on sulfone **1** (Scheme 1C), triazoles **2** proved to be competent substrates in this reaction mediated by magnesium metal, and three examples of *N*-difluoro(trimethylsilyl)methyl-1,2,3-triazoles (**3a**, **3b**, and **3c**) were obtained in high yields (Scheme 2).

**Scheme 3:**
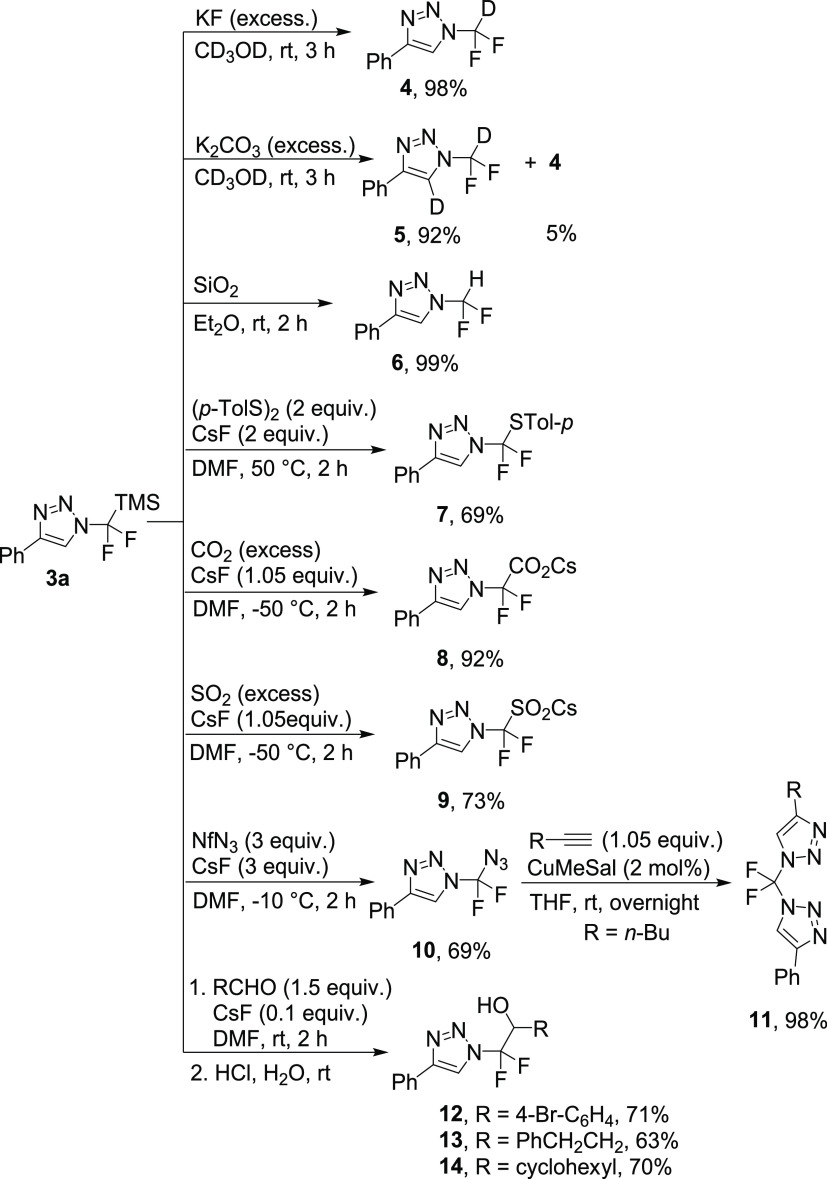
Reaction of *N*-Difluoro(trimethylsilyl)methyl-1,2,3-triazole **3a** with Electrophiles

Triazole **3a** was investigated as a substrate for *N*-difluoromethyl group manipulation (Scheme 3). In the presence of potassium fluoride in methanol-*d*_4_, **3a** is converted to N-CF_2_D triazole **4** in almost quantitative yield. An analogous reaction, only using the stronger base potassium carbonate, capable of deprotonating the triazole at position five, promoted the formation of the doubly deuterated triazole **5**. Silica gel converted triazole **3a** into *N*-difluoromethyl triazole **6** in quantitative yield. Other electrophiles were also tested with good success. Ditolyl disulfide afforded product **7** in good yield. Carbon dioxide or sulfur dioxide were also competent electrophiles in this anion transfer reaction and cesium carboxylate **8** and sulfinate **9**, respectively, were isolated in high yields. Nonaflyl azide as the electrophile yielded the unique *N*-azidodifluoromethyl triazole **10** ready for the next click reaction to form the asymmetrical bis(triazole) **11**. The use of various aryl and alkyl aldehydes as electrophiles produced secondary alcohols **12–14**.

**Scheme 4:**
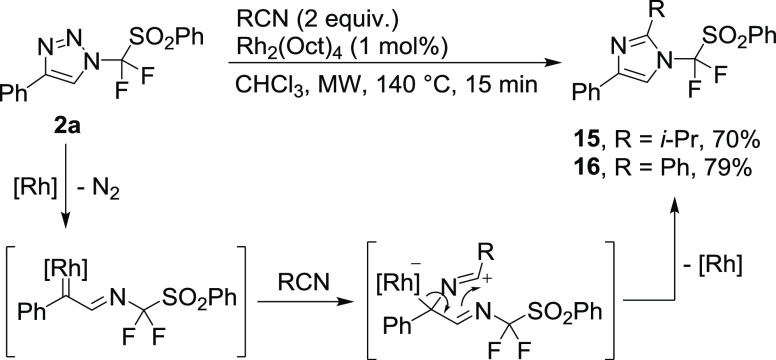
Synthesis of *N*-Difluoro(phenylsulfonyl)methyl-imidazoles **15** and **16** by Rh(II)-Catalyzed Transannulation

In 2018, we published rhodium-catalyzed transannulation reactions of *N*-perfluoroalkyl-1,2,3-triazoles to access various *N*-perfluoroalkylated five-membered nitrogen heterocycles.^23^ The application of this methodology to triazole **2a** and aryl or alkyl nitriles under short microwave heating provided new imidazoles with *N*-difluoro(phenylsulfonyl)methyl functionality **15** and **16** in good yields (Scheme 4).

## Conclusions

In conclusion, azidodifluoromethyl phenyl sulfone (**1**), a new stable azide, was prepared on a multi-gram scale in 90% yield from the commercially available reagents difluoromethyl phenyl sulfone and tosyl azide. Copper(I)-catalyzed azide–alkyne cycloaddition of structurally diverse terminal alkynes and azide **1** proceeded with high efficiency, yielding 1,2,3-triazoles with a difluoro(phenylsulfonyl)methyl substitution on the nitrogen atom. Reductive desulfonylation/silylation of those triazoles with Mg/TMSCl afforded triazoles with the N-CF_2_TMS moiety. In addition, nucleophilic triazolyldifluoromethyl transfer to various electrophiles was demonstrated on numerous examples. Finally, rhodium(II)-catalyzed transannulation of *N*-difluoro(phenylsulfonyl)methyl-1,2,3-triazole with nitriles provided *N*-difluoro(phenylsulfonyl)methyl-imidazoles. Although the synthesis of neither BrCF_2_N_3_ nor TMSCF_2_N_3_, intended to serve for the transfer of the azidodifluoromethyl anion or radical, was achieved, the title azide turned out to be an effective synthetic equivalent of the azidodifluoromethyl anion, making it possible to obtain new substituted N-difluoromethyl triazoles and imidazoles with potential applications in the life sciences.

## Experimental Section

### Materials and Methods

All synthetic reactions were carried out in oven-dried vessels under a dry N_2_ atmosphere. All chemicals were obtained from commercial sources and used as received. THF was freshly distilled over Na/benzophenone prior to use. CDCl_3_ and DMF were dried using molecular sieves (3 and 4 Å, respectively). Microwave heating was performed using sealed flasks on a CEM Discover System 908010. Automated flash column chromatography was performed on a Teledyne ISCO CombiFlash Rf^+^ Lumen automated flash chromatography System with UV/vis detection. ^1^H, ^13^C, and ^19^F NMR spectra were measured at ambient temperature using 5 mm diameter NMR tubes. The chemical shift values (δ) are reported in ppm relative to internal Me_4_Si (0 ppm for ^1^H and ^13^C NMR) or residual solvents and internal CFCl_3_ (0 ppm for ^19^F NMR). High-resolution MS spectra (HRMS) were recorded on an LTQ Orbitrap XL using electrospray (ESI) or APCI ionization on a Waters Micromass AutoSpec Ultima or Agilent 7890A GC coupled with Waters GCT Premier orthogonal acceleration TOF detector using electron impact (EI) or chemical ionization (CI). Simultaneous thermogravimetric and differential scan calorimetry (TG-DSC) was carried out using a Setaram Sensys Evo thermal analyzer equipped with a symmetrical balance and a Calvet 3D sensor.

#### Azidodifluoromethyl Phenyl Sulfone (**1**)

Under an argon atmosphere, into a 250 mL round-bottom flask containing difluoromethyl phenyl sulfone (5.70 g, 29.66 mmol) and tosyl azide (5.88 g, 29.66 mmol) in DMF (33 mL) at −50 °C, a solution of *t*-BuOK (16.60 g, 148 mmol) in DMF (70 mL) was added slowly along the wall. The reaction mixture was stirred at −50 °C for 2 h, and the completion was monitored by ^19^F NMR. The reaction was quenched with 2 M aqueous HCl (50 mL) at −50 °C, followed by warming to room temperature. Then, the mixture was extracted with Et_2_O (4 × 40 mL); the organic layer was washed with saturated NaHCO_3_ (5 × 50 mL) and then with distilled water (5 × 50 mL). The organic phase was dried (MgSO_4_), filtered, and the solvent was removed under reduced pressure, affording **1** (6.20 g, 90%) as a pale-yellow oil. ^1^H NMR (400 MHz, CDCl_3_) δ: 8.04–8.01 (m, 2H), 7.84–7.79 (m, 1H), 7.68–6.64 (m, 2H); ^13^C{^1^H} NMR (101 MHz, CDCl_3_) δ 136.3, 132.1, 130.8, 129.9, 118.9 (t, ^1^*J*_C–F_ = 302.9 Hz); ^19^F NMR (376 MHz, CDCl_3_) δ: −86.9 (s, 2F); Anal. calcd for C_7_H_5_N_3_O_2_F_2_S: C, 36.05; H, 2.16; N, 18.02. Found: C, 36.31; H, 2.22; N, 17.81.

#### General Procedure for the Synthesis of Triazoles **2**

To a solution of **1** (1.0 mmol) and alkyne (1.05 mmol, 1.05 equiv) in THF (3.5 mL) in a 10 mL screw-cap glass, copper(I) 3-methylsalicylate (0.02 mmol, 2 mol %) was added. The flask was closed and stirred at rt overnight (45–50 °C for **2f**, **2h**, and **2l**) (^19^F NMR monitoring). Once the reaction was completed, the solvent was evaporated under reduced pressure, and the crude product was purified by column chromatography on silica gel.

##### 1-(Difluoro(phenylsulfonyl)methyl)-4-phenyl-1*H*-1,2,3-triazole (**2a**)

Purified by column chromatography (cyclohexane/EtOAc, 3:1) and obtained as a white solid. Yield 300 mg, 90%; scale-up 6.30 g, 91%; ^1^H NMR (400 MHz, CDCl_3_) δ: 8.23 (s, 1H), 7.96–7.94 (m, 2H), 7.89–7.82 (m, 3H), 7.68–7.64 (m, 2H), 7.50–7.41 (m, 3H); ^13^C{^1^H} NMR (101 MHz, CDCl_3_) δ: 148.8, 136.9, 131.2, 130.7, 130.0, 129.4, 129.2, 128.8, 126.3, 118.9, 116.2 (t, ^1^*J*_C–F_ = 306.2 Hz); ^19^F NMR (376 MHz, CDCl_3_) δ: −92 (s, 2F); HRMS (APSI^+^) *m*/*z* calcd for C_15_H_12_F_2_N_3_O_2_S [M + H]^+^: 336.0613, found 336.0610; CCDC2234805 (crystal obtained by slow solvent evaporation from CDCl_3_ solution).

##### Methyl 4-(1-(Difluoro(phenylsulfonyl)methyl)-1*H*-1,2,3-triazol-4-yl)benzoate (**2b**)

Purified by column chromatography (cyclohexane/EtOAc, 9:1) and obtained as a white solid. Yield 393 mg, quant.; ^1^H NMR (400 MHz, CDCl_3_) δ: 8.33 (s, 1H), 8.16–8.13 (m, 2H), 7.98–7.95 (m, 4H), 7.88–7.84 (m, 1H), 7.70–7.65 (m, 2H), 3.95 (s, 3H); ^13^C{^1^H} NMR (101 MHz, CDCl_3_) δ: 166.6, 147.8, 137.0, 133.0, 131.2, 130.9, 130.6, 130.5, 130.1, 126.2, 119.9, 116.2 (t, ^1^*J*_C–F_ = 306.6 Hz), 52.4; ^19^F NMR (376 MHz, CDCl_3_) δ: −91.8 (s, 2F); HRMS (ESI^+^) *m*/*z* calcd for C_17_H_14_F_2_N_3_O_4_S [M + H]^+^: 394.0668, found 394.0673.

##### 1-(Difluoro(phenylsulfonyl)methyl)-4-(4-methoxyphenyl)-1*H*-1,2,3-triazole (**2c**)

Purified by column chromatography (cyclohexane/EtOAc, 9:1) and obtained as a white solid. Yield 290 mg, 80%; ^1^H NMR (400 MHz, CDCl_3_) δ: 8.13 (s, 1H), 7.96–7.93 (m, 2H), 7.86–7.78 (m, 3H), 7.67–7.63 (m, 2H), 7.01–6.97 (m, 2H), 3.86 (s, 3H); ^13^C{^1^H} NMR (101 MHz, CDCl_3_) δ: 160.6, 148.7, 136.8, 131.2, 130.7, 130.00, 127.7, 121.3, 117.9, 116.17, 114.6 (t, ^1^*J*_C–F_ = 305.9 Hz), 55.5; ^19^F NMR (376 MHz, CDCl_3_) δ: −92 (s, 2F); HRMS (ESI^+^) *m*/*z* calcd for C_16_H_14_F_2_N_3_O_3_S [M + H]^+^: 366.0718, found 366.0716.

##### 1-(Difluoro(phenylsulfonyl)methyl)-4-(naphthalen-1-yl)-1*H*-1,2,3-triazole (**2d**)

Purified by column chromatography (cyclohexane/EtOAc, 9:1) and obtained as a white solid. Yield 235 mg, 61%; ^1^H NMR (400 MHz, CDCl_3_) δ: 8.29 (s, 1H), 8.26–8.23 (m, 1H), 8.0–7.92 (m, 4H), 7.86–7.82 (m, 1H), 7.78–7.75 (m, 1H), 7.68–7.63 (m, 2H), 7.57–7.53 (m, 3H); ^13^C{^1^H} NMR (101 MHz, CDCl_3_) δ: 147.9, 136.9, 133.9, 131.1, 131.0, 130.7, 130.1, 130.0, 128.8, 128.0, 127.3, 126.4, 126.0, 125.4, 124.9, 122.0, 116.3 (t, ^1^*J*_C–F_ = 306.2 Hz); ^19^F NMR (376 MHz, CDCl_3_) δ: −91.9 (s, 2F); HRMS (ESI^+^) *m*/*z* calcd for C_19_H_14_F_2_N_3_O_2_S [M + H]^+^: 386.0769, found 386.0771.

##### 3-(1-(Difluoro(phenylsulfonyl)methyl)-1*H*-1,2,3-triazol-4-yl)pyridine (**2e**)

Purified by column chromatography (cyclohexane/EtOAc, 9:1) and obtained as a white solid. Yield 307 mg, 91%; ^1^H NMR (400 MHz, CDCl_3_) δ: 9.08 (s, 1H), 8.66–8.65 (m, 1H), 8.33 (s, 1H), 8.24–8.21 (m, 1H), 7.98–7.97 (m, 2H), 7.88–7.84 (m, 1H), 7.70–7.64 (m, 2H), 7.43–7.40 (m, 1H); ^13^C{^1^H} NMR (101 MHz, CDCl_3_) δ: 150.5, 147.5, 145.9, 137.0, 133.7, 131.2, 130.6, 130.1, 125.0, 124.0, 119.5, 116.2 (t, ^1^*J*_C–F_ = 306.7 Hz); ^19^F NMR (376 MHz, CDCl_3_) δ: −91.7 (s, 2F); HRMS (ESI^+^) *m*/*z* calcd for C_14_H_11_F_2_N_4_O_2_S [M + H]^+^: 337.0565, found 337.0566.

##### 4-Butyl-1-(difluoro(phenylsulfonyl)methyl)-1*H*-1,2,3-triazole (**2f**)

Purified by column chromatography (cyclohexane/EtOAc, 9:1) and obtained as a white solid. Yield 162 mg, 50%; ^1^H NMR (400 MHz, CDCl_3_) δ: 7.92–7.89 (m, 2H), 7.85–7.81 (m, 1H), 7.74 (s, 1H), 7.66–7.62 (m, 2H), 2.78(t, ^3^*J*_H–H_ = 7.6 Hz, 2H), 1.73–1.66 (m, 2H), 1.45–1.35 (m, 2H), 0.95 (t, ^3^*J*_H–H_ = 7.5 Hz, 3H); ^13^C{^1^H} NMR (101 MHz, CDCl_3_) δ: 149.5, 136.6, 131.0, 130.8, 129.8, 120.2, 116.0 (t, ^1^*J*_C–F_ = 305.2 Hz), 31.0, 25.0, 22.2, 13.7; ^19^F NMR (376 MHz, CDCl_3_) δ: −91.9 (s, 2F); HRMS (ESI^+^) *m*/*z* calcd for C_13_H_16_F_2_N_3_O_2_S [M + H]^+^: 316.0926, found 316.0926.

##### 1-(Difluoro(phenylsulfonyl)methyl)-4-(4-nitrophenyl)-1*H*-1,2,3-triazole (**2g**)

Purified by column chromatography (cyclohexane/EtOAc, 9:1) and obtained as a white solid. Yield 237 mg, 62%; ^1^H NMR (400 MHz, DMSO-*d*_6_) δ: 9.67 (s, 1H), 8.38–8.66 (m, 2H), 8.25–8.23 (m, 2H), 8.05–7.98 (m, 3H), 7.88–7.79 (m, 2H); ^13^C{^1^H} NMR (101 MHz, DMSO-*d*_6_) δ: 147.5, 145.9, 137.7, 134.7, 130.8, 130.6, 129.2, 126.8, 124.5, 123.5, 115.7 (t, ^1^*J*_C–F_ = 306.2 Hz); ^19^F NMR (376 MHz, DMSO-*d*_6_) δ: −90.9 (s, 2F); HRMS (ESI^+^) *m*/*z* calcd for C_15_H_11_F_2_N_4_O_4_S [M + H]^+^: 381.0464, found 381.0461.

##### 4-(3-Chloropropyl)-1-(difluoro(phenylsulfonyl)methyl)-1*H*-1,2,3-triazole (**2h**)

Purified by column chromatography (cyclohexane/EtOAc, 9:1) and obtained as a white solid. Yield 240 mg, 71%; ^1^H NMR (400 MHz, CDCl_3_) δ: 7.55–7.52 (m, 2H), 7.47–7.42 (m, 2H), 7.28–7.24 (m, 2H), 3.19 (t, ^3^*J*_H–H_ = 6.3 Hz, 2H), 2.58 (t, ^3^*J*_H–H_ = 7.9 Hz, 2H), 1.85–1.78 (m, 2H); ^13^C{^1^H} NMR (101 MHz, CDCl_3_) δ: 147.5, 136.7, 131.0, 130.7, 129.9, 120.8, 116.0 (t, ^1^*J*_C–F_ = 305.6 Hz); 43.8, 31.3, 22.4; ^19^F NMR (376 MHz, CDCl_3_) δ: −91.7 (s, 2F); HRMS (ESI^+^) *m*/*z* calcd for C_12_H_13_ClF_2_N_3_O_2_S [M + H]^+^: 336.0380, found 336.0379.

##### 2-(1-(Difluoro(phenylsulfonyl)methyl)-1*H*-1,2,3-triazol-4-yl)propan-2-ol (**2i**)

Purified by column chromatography (cyclohexane/EtOAc, 9:1) and obtained as a white solid. Yield 258 mg, 80%; ^1^H NMR (400 MHz, CDCl_3_) δ: 7.93–7.89 (m, 3H), 7.84–7.80 (m, 1H), 7.66–7.61 (m, 2H), 2.82 (s, 1H), 1.64 (s, 6H); ^13^C{^1^H} NMR (101 MHz, CDCl_3_) δ: 156.9, 136.9, 136.8, 131.1, 130.7, 130.0, 119.3, 116.1 (t, ^1^*J*_C–F_ = 306 Hz), 68.7, 30.4; ^19^F NMR (376 MHz, CDCl_3_) δ: −91 (s, 2F); HRMS (ESI^+^) *m*/*z* calcd for C_12_H_13_F_2_N_3_O_3_SNa [M + Na]^+^: 340.0538, found 340.0539.

##### 1-(Difluoro(phenylsulfonyl)methyl)-4-((1-phenylethoxy)methyl)-1*H*-1,2,3-triazole (**2j**)

Purified by column chromatography (cyclohexane/EtOAc, 9:1) and obtained as a white solid. Yield 307 mg, 76%; ^1^H NMR (400 MHz, CDCl_3_) δ: 7.95–7.81 (m, 3H), 7.85–7.81 (m, 1H), 7.66–7.62 (m, 2H), 7.39–7.28 (m, 5H), 4.86 (q, ^3^*J*_H–H_ = 6.5 Hz, 1H), 4.56 (s, 2H), 1.61(d, ^3^*J*_H–H_ = 6.5 Hz, 6H); ^13^C{^1^H} NMR (101 MHz, CDCl_3_) δ: 151.8, 137.9, 136.8, 131.1, 130.8, 130.0, 128.6, 127.94, 127.87, 116.2 (t, ^1^*J*_C–F_ = 306.1 Hz), 71.0, 69.5, 21.5; ^19^F NMR (376 MHz, CDCl_3_) δ: −91.7 (s, 2F); HRMS (ESI^+^) *m*/*z* calcd for C_18_H_17_F_2_N_3_O_3_SNa [M + Na]^+^: 416.0851, found 416.0851.

##### 1-(Difluoro(phenylsulfonyl)methyl)-4-ethoxy-1*H*-1,2,3-triazole (**2k**)

Purified by column chromatography (cyclohexane/EtOAc, 9:1) and obtained as a white solid. Yield 226 mg, 75%; ^1^H NMR (400 MHz, CDCl_3_) δ: 7.93–7.89 (m, 2H), 7.85–7.80 (m, 1H), 7.67–7.61 (m, 2H), 7.41 (s, 1H), 4.30 (q, ^3^*J*_H–H_ = 6.9 Hz, 2H), 1.43 (t, ^3^*J*_H–H_ = 7.0 Hz, 3H); ^13^C{^1^H} NMR (101 MHz, CDCl_3_) δ: 161.1, 136.8, 131.1, 130.8, 130.0, 115.9 (t, ^1^*J*_C–F_ = 306.6 Hz), 105.2, 67.3, 14.8; ^19^F NMR (376 MHz, CDCl_3_) δ: −92.9 (s, 2F); HRMS (ESI^+^) *m*/*z* calcd for C_11_H_12_F_2_N_3_O_3_S [M + H]^+^: 304.0562, found 304.0559.

##### 4-Cyclopropyl-1-(difluoro(phenylsulfonyl)methyl)-1*H*-1,2,3-triazole (**2l**)

Purified by column chromatography (cyclohexane/EtOAc, 9:1) and obtained as a white solid. Yield 276 mg, 92%; ^1^H NMR (400 MHz, CDCl_3_) δ: 7.93–7.89 (m, 2H), 7.85–7.79 (m, 1H), 7.70 (s, 1H), 7.66–7.62 (m, 2H), 2.03–1.96 (m, 2H), 0.94–0.91 (m, 2H); ^13^C{^1^H} NMR (101 MHz, CDCl_3_) δ: 151.4, 136.7, 129.9, 119.3, 116.0 (t, ^1^*J*_C–F_ = 305.2 Hz), 8.2, 6.6; ^19^F NMR (376 MHz, CDCl_3_) δ: −91.8 (s, 2F); HRMS (ESI^+^) *m*/*z* calcd for C_12_H_12_F_2_N_3_O_2_S [M + H]^+^: 300.0613, found 300.0610.

##### 4-Cyclopentyl-1-(difluoro(phenylsulfonyl)methyl)-1*H*-1,2,3-triazole (**2m**)

Purified by column chromatography (cyclohexane/EtOAc, 9:1) and obtained as a white solid. Yield 250 mg, 76%; ^1^H NMR (400 MHz, CDCl_3_) δ: 7.90–7.88 (m, 2H), 7.83–7.79 (m, 1H), 7.70 (s, 1H), 7.64–7.60 (m, 2H), 3.25–3.17 (m, 1H), 2.15–2.07 (m, 2H), 1.81–1.63 (m, 6H); ^13^C{^1^H} NMR (101 MHz, CDCl_3_) δ: 153.8, 136.7, 131.0, 130.8, 129.9, 119.3, 116.1 (t, ^1^*J*_C–F_ = 305.1 Hz), 36.4, 33.0, 25.2; ^19^F NMR (376 MHz, CDCl_3_) δ: −91.7 (s, 2F); HRMS (ESI^+^) *m*/*z* calcd for C_14_H_16_F_2_N_3_O_2_S [M + H]^+^: 328.0926, found 328.0926.

##### 4-Cyclohexyl-1-(difluoro(phenylsulfonyl)methyl)-1*H*-1,2,3-triazole (**2n**)

Purified by column chromatography (cyclohexane/EtOAc, 9:1) and obtained as a white solid. Yield 297 mg, 87%; ^1^H NMR (400 MHz, CDCl_3_) δ: 7.89–7.87 (m, 2H), 7.83–7.78 (m, 1H), 7.68 (s, 1H), 7.63–7.60 (m, 2H), 2.84–2.75 (m, 1H), 2.10–2.01 (m, 2H), 1.85–1.77 (m, 2H), 1.75–1.69 (m, 1H), 1.43–1.33 (m, 4H), 1.30–1.19 (m, 1H); ^13^C{^1^H} NMR (101 MHz, CDCl_3_) δ: 154.7, 136.7, 131.0, 130.8, 129.9, 119.2, 116.1 (t, ^1^*J*_C–F_ = 305.4 Hz), 35.0, 32.6, 26.0, 25.9; ^19^F NMR (376 MHz, CDCl_3_) δ: −91.8 (s, 2F); HRMS (ESI^+^) *m*/*z* calcd for C_15_H_18_F_2_N_3_O_2_S [M + H]^+^: 342.1082, found 342.1078.

##### 4-(Cyclohex-1-en-1-yl)-1-(difluoro(phenylsulfonyl)methyl)-1*H*-1,2,3-triazole (**2o**)

Purified by column chromatography (cyclohexane/EtOAc, 9:1) and obtained as a white solid. Yield 240 mg, 92%; ^1^H NMR (400 MHz, CDCl_3_) δ: 7.93–7.90 (m, 2H), 7.85–7.81 (m, 1H), 7.79 (s, 1H), 7.69–7.62 (m, 2H), 6.72–6.69 (m, 1H), 2.39–2.34 (m, 2H), 2.26–2.22 (m, 2H), 1.82–1.77 (m, 2H), 1.72–1.66 (m, 2H); ^13^C{^1^H} NMR (101 MHz, CDCl_3_) δ: 150.4, 136.7, 131.1, 130.9, 130.0, 128.2, 125.8, 116.2 (t, ^1^*J*_C–F_ = 304.6 Hz), 26.4, 25.4, 22.4, 22.1; ^19^F NMR (376 MHz, CDCl_3_) δ: −91.9 (s, 2F); HRMS (ESI^+^) *m*/*z* calcd for C_15_H_16_F_2_N_3_O_2_S [M + H]^+^: 340.0926, found 340.0925.

##### 4,4′-(Oxybis(methylene))bis(1-(difluoro(phenylsulfonyl)methyl)-1*H*-1,2,3-triazole) (**2p**)

Purified by column chromatography (cyclohexane/EtOAc, 9:1) and obtained as a white solid. Yield 300 mg, 79%; ^1^H NMR (400 MHz, CDCl_3_) δ: 8.07 (s, 2H), 7.94–7.91 (m, 4H), 7.85–7.81 (m, 2H), 7.67–7.62 (m, 4H), 4.80(s, 4H); ^13^C{^1^H} NMR (101 MHz, CDCl_3_) δ: 145.6, 136.9, 131.1, 130.5, 130.0, 122.7, 116.1 (t, ^1^*J*_C–F_ = 306.4 Hz), 63.4; ^19^F NMR (376 MHz, CDCl_3_) δ: −91.6 (s, 2F); HRMS (ESI^+^) *m*/*z* calcd for C_20_H_17_F_4_N_6_O_5_S_2_ [M + H]^+^: 561.0633, found 561.0632.

#### General Procedure for the Synthesis of Triazoles **3** via Reductive Desulfonylation/Silylation

To an oven-dried high-pressure tube containing Mg turnings (43.8 mg, 1.8 mmol) in dry DMF (2.5 mL), TMSCl (570 μL, 4.5 mmol) was added under N_2_ and stirred for 2 min at 0 °C. A solution of the corresponding **2** (0.9 mmol) in DMF (3 mL) was added to the mixture and stirred for 1 h at 0 °C and 2 h at room temperature (^19^F NMR monitoring). The reaction mixture was poured over water/ice and extracted with Et_2_O. The organic layer was washed with water (2–3 times), dried (MgSO_4_), and filtered, and the solvent was removed under reduced pressure to yield pure **3**.

##### 1-(Difluoro(trimethylsilyl)methyl)-4-phenyl-1*H*-1,2,3-triazole (**3a**)

Yield 300 mg, 90%, scale-up 1.20 g, 91%; ^1^H NMR (400 MHz, CDCl_3_) δ: 8.14 (s, 1H), 7.89–7.86 (m, 2H), 7.48–7.43 (m, 2H), 7.40–7.35 (m, 1H), 0.44 (s, 9H); ^13^C{^1^H} NMR (101 MHz, CDCl_3_) δ: 148.2, 129.8, 129.1, 128.9, 126.1, 123.6 (t, ^1^*J*_C–F_ = 282.5 Hz), 116.1, −3.8; ^19^F NMR (376 MHz, CDCl_3_) δ: −87 (s, 2F); HRMS (ESI^+^) *m*/*z* calcd for C_12_H_16_F_2_N_3_Si [M + H]^+^: 268.1076, found 268.1075.

##### 1-(Difluoro(trimethylsilyl)methyl)-4-(4-methoxyphenyl)-1*H*-1,2,3-triazole (**3b**)

Yield 131 mg, 88%; ^1^H NMR (400 MHz, CDCl_3_) δ: 8.05 (s, 1H), 7.80–7.78 (m, 2H), 6.99–6.96 (m, 2H), 3.85 (s, 3H), 0.43 (s, 9H); ^13^C{^1^H} NMR (101 MHz, CDCl_3_) δ: 160.1, 148.1, 127.4, 123.6 (t, ^1^*J*_C–F_ = 283.5 Hz), 122.5, 115.1, 114.5, 55.4, −3.8; ^19^F NMR (376 MHz, CDCl_3_) δ: −87 (s, 2F); HRMS (EI^+^) *m*/*z* calcd for C_13_H_17_F_2_N_3_OSi [M + H]^+^: 297.1103, found 297.1110.

##### 4-Cyclohexyl-1-(difluoro(trimethylsilyl)methyl)-1*H*-1,2,3-triazole (**3c**)

Yield 107 mg, 78%; ^1^H NMR (400 MHz, CDCl_3_) δ: 7.60 (s, 1H), 2.80–2.73 (m, 1H), 2.11–2.02 (m, 2H), 1.81–1.68 (m, 3H), 1.41–1.33 (m, 5H), 1.30–1.22 (m, 1H), 0.37 (s, 9H); ^13^C{^1^H} NMR (101 MHz, CDCl_3_) δ: 154.0, 123.4 (t, ^1^*J*_C–F_ = 282.4 Hz), 115.8, 35.2, 32.8, 26.14, 26.09, −3.8; ^19^F NMR (376 MHz, CDCl_3_) δ: −86.7 (s, 2F); HRMS (EI^+^) *m*/*z* calcd for C_12_H_21_F_2_N_3_Si [M + H]^+^: 273.1467, found 273.1474.

##### Synthesis of 1-(Difluoromethyl-*d*)-4-phenyl-1*H*-1,2,3-triazole **4**

To a solution of **3a** (137 mg, 0.5 mmol) in CD_3_OD (3 mL), finely ground dry KF (58 mg, 1 mmol) was added. After stirring for 2 h at room temperature, the solvent was removed under reduced pressure, and the product was dissolved in Et_2_O. Filtration and solvent removal afforded pure **4** as a pale-yellow solid. Yield 97 mg, 98%. ^1^H NMR (400 MHz, CDCl_3_) δ: 8.15 (s, 1H), 7.89–7.84 (m, 2H), 7.47–7.43 (m, 2H), 7.40–7.37 (m, 1H); ^13^C{^1^H} NMR (101 MHz, CDCl_3_) δ: 149.2, 129.19, 129.13, 129.08,126.1, 115.8, 109.7 (tt, ^1^*J*_C–F_ = 252.7 Hz, ^1^*J*_C–D_ = 32.5 Hz); ^19^F NMR (376 MHz, CDCl_3_) δ: −96.4 (t, ^2^*J*_F–D_ = 9.1 Hz, 2F); HRMS (EI^+^) *m*/*z* calcd for C_9_H_7_DF_2_N_3_ [M + H]^+^: 197.0744, found 197.0744.

##### Synthesis of 1-(Difluoromethyl-*d*)-4-phenyl-1*H*-1,2,3-triazole-5-*d***5**

To a solution of **3a** (137 mg, 0.5 mmol) in CD_3_OD (3 mL), finely ground dry K_2_CO_3_ (135.2 mg, 1 mmol) was added. After stirring for 2 h at room temperature, the solvent was removed under reduced pressure, and the product was dissolved in Et_2_O. Filtration and solvent removal afforded pure **5** as a pale-yellow solid. Yield 90 mg, 92%. ^1^H NMR (400 MHz, CDCl_3_) δ: 7.88–7.86 (m, 2H), 7.48–7.45 (m, 2H), 7.42–7.38 (m, 1H); ^13^C{^1^H} NMR (101 MHz, CDCl_3_) δ: 149.2, 129.23, 129.21, 129.16, 126.2, 115.6 (t, ^1^*J*_C–D_ = 30,4 Hz), 109.8 (tt, ^1^*J*_C–F_ = 252.8 Hz, ^1^*J*_C–D_ = 32.4 Hz); ^19^F NMR (376 MHz, CDCl_3_) δ: −96.4 (t, ^2^*J*_F–D_=9 Hz, 2F); HRMS (ESI^+^) *m*/*z* calcd for C_9_H_6_D_2_F_2_N_3_ [M + H]^+^: 198.0806, found 198.0808.

##### Synthesis of 1-(Difluoromethyl)-4-phenyl-1*H*-1,2,3-triazole **6**

**3a** (200 mg, 0.75 mmol) was dissolved in Et_2_O (50 mL), and silica gel (40–50 mg) was added. This heterogeneous mixture was stirred at room temperature for 2 h and filtered, and the solvent was removed under reduced pressure, affording pure **6** as a colorless crystalline solid (145 mg, 99%). ^1^H NMR (400 MHz, CDCl_3_) δ 8.16 (s, 1H), 7.89–7.86 (m, 2H), 7.59 (t, *J* = 59.1 Hz, 1H), 7.50–7.44 (m, 2H), 7.43–7.38 (m, 1H); ^13^C, ^19^F NMR and HRMS data corresponded to previously published ones.^24^

##### Synthesis of 1-(Difluoro(*p*-tolylthio)methyl)-4-phenyl-1*H*-1,2,3-triazole (**7**)

Ditolyl disulfide (246 mg, 1 mmol) and anhydrous CsF (152 mg, 1 mmol) were dissolved in DMF (2.5 mL) under N_2_. A solution of **3a** (134 mg, 0.5 mmol) in DMF (2.5 mL) was added, and the mixture was heated for 2 h at 80 °C. The solution was cooled to room temperature, water (2.5 mL) was added, and the product was extracted with Et_2_O. The organic phase was washed with brine, dried (MgSO_4_), and filtered, and the solvent was removed under reduced pressure. The residue was purified by silica-gel column chromatography (dichloromethane/hexane, 5:95) to afford **7** as white solid (**7**): Yield 110 mg, 69%; ^1^H NMR (400 MHz, CDCl_3_) δ: 7.95 (s, 1H), 7.82–7.79 (m, 2H), 7.51–7.49 (m, 2H), 7.46–7.35 (m, 3H), 7.19–7.17 (m, 2H), 2.36 (s, 3H); ^13^C{^1^H} NMR (101 MHz, CDCl_3_) δ: 148.1, 142.0, 137.0, 130.5, 129.4, 129.1, 129.0, 126.2, 124.4 (t, ^1^*J*_C–F_ = 290 Hz), 120.8, 117.6, 21.5; ^19^F NMR (376 MHz, CDCl_3_) δ: −56.4 (s, 2F); HRMS (ESI^+^) *m*/*z* calcd for C_16_H_14_F_2_N_3_S [M + H]^+^: 318.0871, found 318.0871.

#### General Procedure for the Synthesis of Salts **8** and **9**

An oven-dried two-neck flask was evacuated, backfilled with argon, and then charged with anhydrous CsF (160 mg, 1.05 mmol, 1.05 equiv) and anhydrous DMF (20 mL). Dry SO_2_ or CO_2_ was bubbled into the solution, followed by cooling the solution to −50 °C, and the bubbling was continued for 30 min at −50 °C. Then, a solution of **3a** (267 mg, 1 mmol, 1.0 equiv) in anhydrous DMF (3 mL) was added dropwise. Stirring was continued for another 60 min at −50 °C, and then the reaction mixture was slowly warmed to room temperature, and the solvent was removed under reduced pressure. The solid residue was triturated with anhydrous Et_2_O, filtered, and washed on filter with Et_2_O several times.

##### Cesium 2,2-Difluoro-2-(4-phenyl-1*H*-1,2,3-triazol-1-yl)acetate (**8**)

Yellow solid. Yield 340 mg, 92%; ^1^H NMR (400 MHz, D_2_O) δ: 8.56 (s, 1H), 7.70–7.66 (m, 2H), 7.45–7.36 (m, 3H); ^13^C{^1^H} NMR (101 MHz, D_2_O) δ: 162.9 (t, ^1^*J*_F–C_ = 29.2 Hz), 147.6, 129.12, 129.05, 128.4, 125.8, 119.6, 110.6 (t, ^1^*J*_C–F_ = 270.4 Hz); ^19^F NMR (376 MHz, D_2_O) δ: −87 (s, 2F); HRMS (ESI^–^) *m*/*z* calcd for C_10_H_6_F_2_N_3_O_2_ [M]^−^: 238.0423, found 238.0423.

##### Cesium Difluoro(4-phenyl-1*H*-1,2,3-triazol-1-yl)methanesulfinate (**9**)

Pale-yellow solid. Yield 290 mg, 73%; ^1^H NMR (400 MHz, D_2_O) δ: 8.44 (s, 1H), 7.72–7.69 (m, 2H), 7.48–7.38 (m, 3H); ^13^C{^1^H} NMR (101 MHz, D_2_O) δ: 147.4, 129.14, 129.10, 128.4, 125.8, 120.2 (t, ^1^*J*_C–F_ = 314.9 Hz); 119.8; ^19^F NMR (376 MHz, D_2_O) δ: −99.1 (s, 2F); HRMS (ESI^–^) *m*/*z* calcd for C_9_H_6_F_2_N_3_O_2_S [M]^−^: 258.0154, found 258.0153.

##### Synthesis of 1-(Azidodifluoromethyl)-4-phenyl-1*H*-1,2,3-triazole (**10**)

Nonaflyl azide (585 mg, 1.80 mmol) and anhydrous CsF (273 mg, 1.8 mmol) were dissolved in DMF (5 mL) under N_2_, followed by cooling the solution to −10 °C. To the resulting mixture, a solution of **3a** (160 mg, 0.6 mmol) in DMF (3 mL) was added dropwise, and the mixture was stirred at −10 °C for 15 min and then slowly warmed to room temperature. The mixture was stirred at room temperature for 2 h, water (4 mL) was added, and the product was extracted with Et_2_O. The organic phase was washed with brine, dried (MgSO_4_), and filtered, and the solvent was removed under reduced pressure. The residue was purified by silica-gel column chromatography (hexane/EtOAc, 9:1) to afford **10** as a colorless liquid. Yield 98 mg, 69%; ^1^H NMR (400 MHz, CDCl_3_) δ: 8.15 (s, 1H), 7.86–7.83 (m, 2H), 7.44–7.34 (m, 3H); ^13^C{^1^H} NMR (101 MHz, CDCl_3_) δ: 148.5, 129.1, 129.0, 128.8, 126.0, 117.5, 116.9 (t, ^1^*J*_C–F_ = 262.3 Hz); ^19^F NMR (376 MHz, CDCl_3_) δ: −58.8 (s, 2F); HRMS (EI^+^) *m*/*z* calcd for C_9_H_6_F_2_N_6_ [M + H]^+^: 236.0617, found 236.0613.

##### Synthesis of 4-Butyl-1-(difluoro(4-phenyl-1*H*-1,2,3-triazol-1-yl)methyl)-1*H*-1,2,3-triazole (**11**)

1-Hexyne (40 mg, 0.48 mmol) was placed into a 10 mL screw-cap glass tube, and a solution of **10** (95 mg, 0.4 mmol) in THF (4 mL) was added. Copper(I) 3-methylsalicylate (2 mg, 8 μmol) was added, the flask was closed, and the mixture was stirred at rt for 18 h. A saturated solution of NH_4_Cl (10 mL) was added, and the product was extracted with DCM (3 × 10 mL). The combined organic phase was washed with water (2 × 10 mL), dried (MgSO_4_), filtered, and concentrated in vacuo. The crude product was purified by column chromatography on silica gel (cyclohexane/EtOAc, 3:1). White solid. Yield 125 mg, 98%; ^1^H NMR (400 MHz, CDCl_3_) δ: 8.30 (s, 1H), 7.85–7.79 (m, 3H), 7.42–7.32 (m, 3H), 2.75 (t, ^3^*J*_H–H_ = 7.4 Hz, 2H), 1.70–1.62 (m, 2H), 1.40–1.32 (m, 2H), 0.90 (^3^*J*_H–H_ = 7.4 Hz, 3H); ^13^C{^1^H} NMR (101 MHz, CDCl_3_) δ: 149.5, 148.5, 129.2, 129.0, 128.6, 126.1, 119.7, 118.4, 113.4 (t, ^1^*J*_C–F_ = 260.2 Hz), 30.9, 25.0, 22.2, 13.7; ^19^F NMR (376 MHz, CDCl_3_) δ: −65.6 (s, 2F); HRMS (ESI^+^) *m*/*z* calcd for C_15_H_16_F_2_N_6_Na [M + Na]^+^: 341.1297, found 341.1298.

#### General Procedure for the Synthesis of Alcohols **12**–**14**

The corresponding aldehyde (0.75 mmol, 1.5 equiv) and anhydrous CsF (0.075 mmol, 10 mol %) were dissolved in DMF (2 mL) under an inert atmosphere. To the resulting solution, a solution of **3a** (134 mg, 0.5 mmol, 1.0 equiv) in DMF (2 mL) was added, and the mixture was stirred for 3 h at room temperature. Ice-cold HCl (2 M, 3 mL) was added, and the product was extracted with Et_2_O. The organic phase was washed with brine, dried (MgSO_4_), and filtered, and the solvent was removed under reduced pressure. The residue was purified by silica-gel column chromatography (hexane/EtOAc, 9:1) to afford pure **12**–**14**.

##### 1-(4-Bromophenyl)-2,2-difluoro-2-(4-phenyl-1*H*-1,2,3-triazol-1-yl)ethan-1-ol (**12**)

White solid. Yield 135 mg, 71%; ^1^H NMR (400 MHz, DMSO-*d*_6_) δ: 9.08 (s, 1H), 7.96–7.94 (m, 2H), 7.62–7,60 (m, 2H), 7.51–7.38 (m, 5H), 7.08 (d, ^3^*J*_H–H_ = 5.8 Hz, 1H), 5.66 (dt, ^3^*J*_H–H_ = 6.2 Hz, ^4^*J*_F–H_ = 15.3 Hz, 1H). ^13^C{^1^H} NMR (101 MHz, DMSO-*d*_6_) δ: 146.8, 135.1, 131.2, 130.1, 129.5, 129.1, 128.7, 125.6, 122.3, 120.3, 118.5(t, ^1^*J*_C–F_ = 261.2 Hz), 71.8 (dd, ^2^*J*_C–F_ = 31.7 Hz, ^2^*J*_C–F_ = 25.3 Hz), ^19^F NMR (376 MHz, DMSO-*d*_6_) δ: −84.4 (d, ^3^*J*_H–F_ = 201.2 Hz, 1F), −96.9 (dd, ^3^*J*_H–F_ = 207.7 Hz, ^4^*J*_H–F_ = 15.3 Hz, 1F), HRMS (ESI^+^) *m*/*z* calcd for C_16_H_13_F_2_N_3_OBr [M + H]^+^: 380.0205, found 380.0204.

##### 1,1-Difluoro-4-phenyl-1-(4-phenyl-1*H*-1,2,3-triazol-1-yl)butan-2-ol (**13**)

White solid. Yield 105 mg, 63%; ^1^H NMR (400 MHz, CDCl_3_) δ: 8.10 (s, 1H), 7.77–7.74 (m, 2H), 7.45–7.21 (m, 8H), 4.68 (m, 1H), 3.67 (s, 1H), 3.08–3.01 (m, 1H) 2.87–2.79 (m, 1H), 2.18–2.08 (m, 2H); ^13^C{^1^H} NMR (101 MHz, CDCl_3_) δ: 148.0, 140.9, 129.12, 129.11, 129.0, 128.74, 128.70, 126.4, 126.0, 119.1 (t, ^1^*J*_C–F_ = 265.7 Hz), 118.1, 70.9 (dd, ^2^*J*_C–F_ = 30.9 Hz, ^2^*J*_C–F_ = 25.0 Hz), 31.4, 30.7; ^19^F NMR (376 MHz, CDCl_3_) δ: −85.5 (d, ^3^*J*_H–F_ = 209.7 Hz, 1F), −98.6 (d, ^3^*J*_H–F_ = 209.6 Hz, 1F), HRMS (ESI^+^) *m*/*z* calcd for C_18_H_18_F_2_N_3_O [M + H]^+^: 330.1413, found 330.1412.

##### 1-Cyclohexyl-2,2-difluoro-2-(4-phenyl-1*H*-1,2,3-triazol-1-yl)ethan-1-ol (**14**)

White solid. Yield 108 mg, 70%; ^1^H NMR (400 MHz, CDCl_3_) δ: 8.09 (s, 1H), 7.74–7.71 (m, 2H), 7.44–7.34 (m, 3H), 4.55–4.46 (m, 1H), 3.71 (d, ^3^*J*_H–H_ = 6.6 Hz, 1H), 1.99–1.89 (m, 2H), 1.83–1.78 (m, 3H), 1.71–1.66 (m, 1H), 1.50–1.14 (m, 6H), ^13^C{^1^H} NMR (101 MHz, CDCl_3_) δ: 147.8, 129.2, 129.1, 129.0, 126.0, 119.8 (t, ^1^*J*_C–F_ = 268.7 Hz), 118.0, 75.0 (dd, ^2^*J*_C–F_ = 30.2 Hz, ^2^*J*_C–F_ = 23.4 Hz), 38.3, 30.1, 27.11, 27.09, 26.4, 26.2, 26.0; ^19^F NMR (376 MHz, CDCl_3_) δ: −83 (d, ^3^*J*_H–F_ = 209 Hz, 1F), −96.5 (dd, ^3^*J*_H–F_ = 209 Hz, ^4^*J*_H–F_ = 19.9 Hz, 1F), HRMS (EI^+^) *m*/*z* calcd for C_16_H_19_F_2_N_3_O [M + H]^+^: 307.1491, found 307.1491.

#### General Procedure for the Synthesis of Imidazoles **15** and **16**

Triazole **2a** (0.5 mmol, 1.0 equiv) was dissolved in anhydrous CHCl_3_ (4 mL) in a microwave tube. Rh_2_(Oct)_4_ (5 μmol, 1 mol %) and the corresponding nitrile (1.0 mmol, 2.0 equiv) were added, the tube was closed, and briefly sonicated. The reaction mixture was heated at 140 °C for 15 min in a microwave reactor. The solvent was evaporated under reduced pressure, and the crude product was purified by column chromatography on silica gel (cyclohexane/EtOAc, 0:100 to 10:90).

##### 1-(Difluoro(phenylsulfonyl)methyl)-2-isopropyl-4-phenyl-1*H*-imidazole (**15**)

White solid. Yield 132 mg, 70%; ^1^H NMR (400 MHz, CDCl_3_) δ: 7.97–7.94 (m, 2H), 7.83–7.79 (m, 1H), 7.76–7.73 (m, 2H), 7.66–7.62 (m, 2H), 7.40–7.35 (m, 2H), 7.30–7.26 (m, 1H), 7.16 (s, 1H), 3.18–3.10 (m, 1H), 1.36 (d, ^3^*J*_H–H_ = 6.7 Hz, 6H);^13^C{^1^H} NMR (101 MHz, CDCl_3_) δ: 155.4, 141.6, 136.3, 132.9, 131.8, 130.9, 129.9, 128.7, 127.7, 125.5, 117.1 (t, ^1^*J*_C–F_ = 302.4 Hz), 112.2, 28.3 (t, ^4^*J*_C–F_ = 3.9 Hz), 22.5; ^19^F NMR (376 MHz, CDCl_3_) δ: −86.9 (s, 2F); HRMS (CI^+^) *m*/*z* calcd for C_19_H_19_F_2_N_2_O_2_S [M + H]^+^: 377.1130, found 377.1129.

##### 1-(Difluoro(phenylsulfonyl)methyl)-2,4-diphenyl-1*H*-imidazole (**16**)

Pale-brown solid. Yield 163 mg, 79%; ^1^H NMR (400 MHz, CDCl_3_) δ: 7.98–7.83 (m, 4H), 7.82–7.78 (m, 1H), 7.62–7.58 (m, 3H), 7.53–7.51 (m, 2H), 7.48–7.38 (m, 5H), 7.35–7.23 (m, 1H); ^13^C NMR (126 MHz, CDCl_3_) δ 148.6, 142.6, 136.4, 132.3, 131.6, 131.0, 130.0, 129.92, 129.90, 128.8, 128.2, 128.0, 125.7, 116.9 (t, ^1^*J*_C–F_ = 304 Hz), 114.0; ^19^F NMR (376 MHz, CDCl_3_) δ: −84.4 (s, 2F); HRMS (EI^+^) *m*/*z* calcd for C_22_H_16_F_2_N_2_O_2_S [M]^+^: 410.0895, found 410.0912.

## Data Availability

The data underlying this study are available in the published article and its Supporting Information.

## Supporting Information Available

DSC and TG analyses; X-ray crystallography, and copies of NMR spectra (PDF)
