# Supplementary material for: Synthesis of Azidodifluoromethyl Phenyl Sulfone and Its Use as a Synthetic Equivalent of the Azidodifluoromethyl Anion
Source: J Org Chem. 2023 May 18;88(11):6939–46. doi: 10.1021/acs.joc.3c00256 (PMC10242755; doi:10.1021/acs.joc.3c00256)
Supplement: Supplementary file 1 — jo3c00256_si_001.pdf [file jo3c00256_si_001.pdf]

Synthesis of Azidodifluoromethyl Phenyl Sulfone and its Use as a Synthetic Equivalent of the Azidodifluoromethyl Anion

Mykyta Ziabko[†,‡] , Blanka Klepetářová[†] and Petr Beier[*,†]

[†]Institute of Organic Chemistry and Biochemistry of the Czech Academy of Sciences, Flemingovo náměstí, 2, 166 10, Prague 6, Czech Republic.

[‡]Department of Organic Chemistry, Faculty of Science, Charles University, Hlavova 2030/8, 128 43 Prague, Czech Republic.

Corresponding author: beier@uochb.cas.cz

Supporting Information Placeholder

Supporting information

Contents

DSC an TG analysis of azide **1**........S1
X-ray crystallography of **2a**........S2
Copies of NMR spectra S3
References........S51

DSC and TG analysis of 1

Figure S1. DSC (blue curve) and TG (green curve) analysis of azide **1** (sample measured on air, range 30-300 °C, velocity 15 K/min).

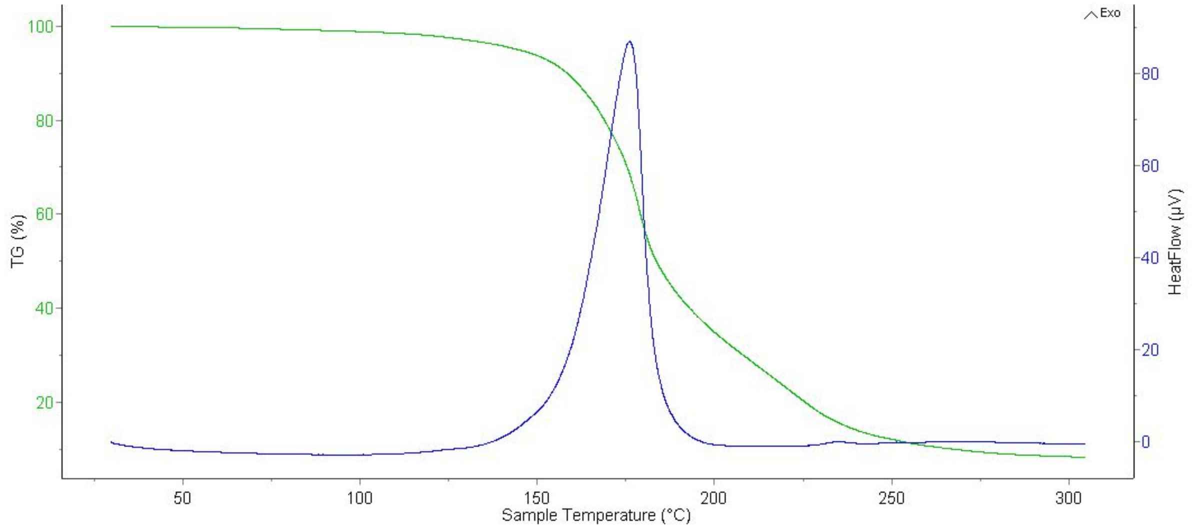

X-ray crystallography

Single-crystal data of **2a** were collected on a Bruker D8 VENTURE system equipped with a Photon 100 CMOS detector, a multilayer monochromator, and a CuKα Incoatec microfocus sealed tube (λ = 1.54178 Å) at 180 K. The data reduction and absorption correction were performed with Apex3[1] software. The structure was solved by direct methods with SIR92[2] and refined by full-matrix least-squares on F with CRYSTALS.[3] The positional and anisotropic thermal parameters of non-hydrogen atoms were refined. The positional and anisotropic thermal parameters of all non-hydrogen atoms were refined. All hydrogen atoms were found from a Fourier difference map and then recalculated into idealized positions and refined with riding constraints.

Crystal data for 2a (colorless, 0.082 x 0.086 x 0.696 mm):

$C_{15}H_{11}F_2N_3O_2S_1$, orthorhombic, space group $P2_12_12_1$, a = 5.3020(2) Å, b = 11.2363(5) Å, c = 24.7407(11) Å, V = 1473.92(11) Å^3, Z = 4, M = 335.33, 15904 reflections measured, 2598 independent reflections. Final R = 0.024, wR = 0.026, GoF = 1.085 for 2401 reflections with $I > 2\sigma(I)$ and 210 parameters. Flack parameter x = 0.073(13). CCDC2234805 (Figure S2).

Figure S2. ORTEP diagram of **2a**, displacement ellipsoids shown with 50% probability.

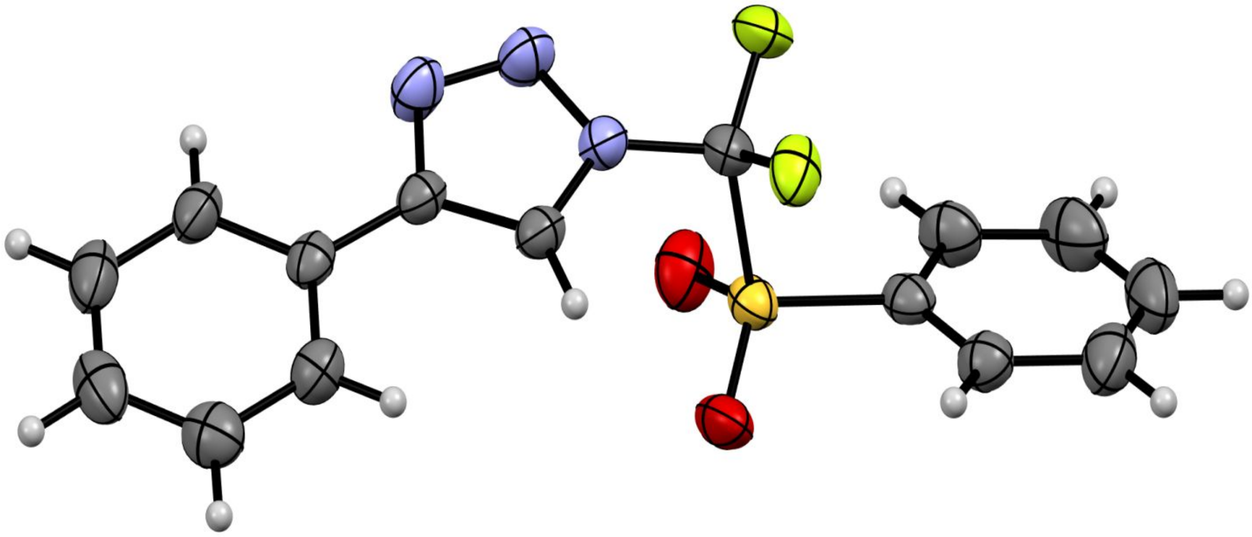

Copies of NMR spectra

Figure S3. ^1H NMR spectrum of **1** ($CDCl_3$, 400 MHz)

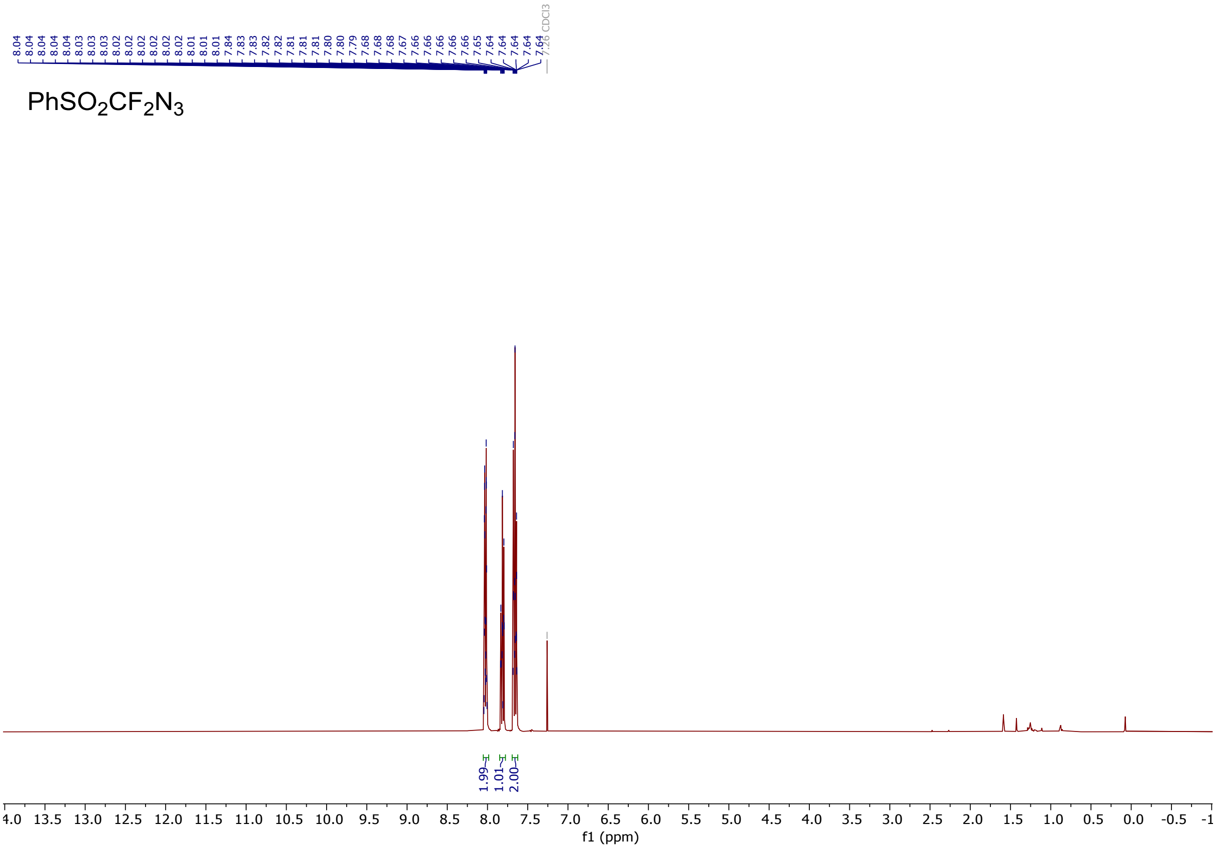

Figure S4. $^{13}C\{^1H\}$ NMR spectrum of **1** ($CDCl_3$, 101 MHz)

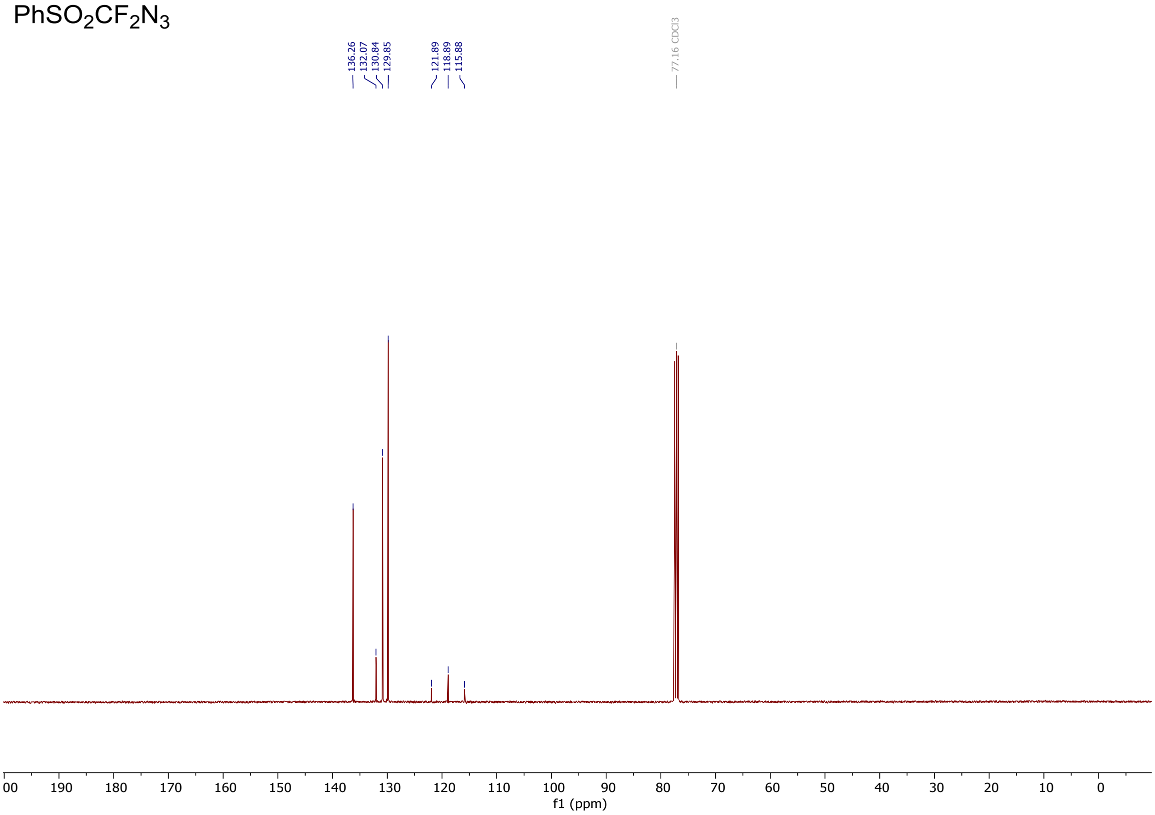

Figure S5. ^{19}F NMR spectrum of **1** ($CDCl_3$, 377 MHz)

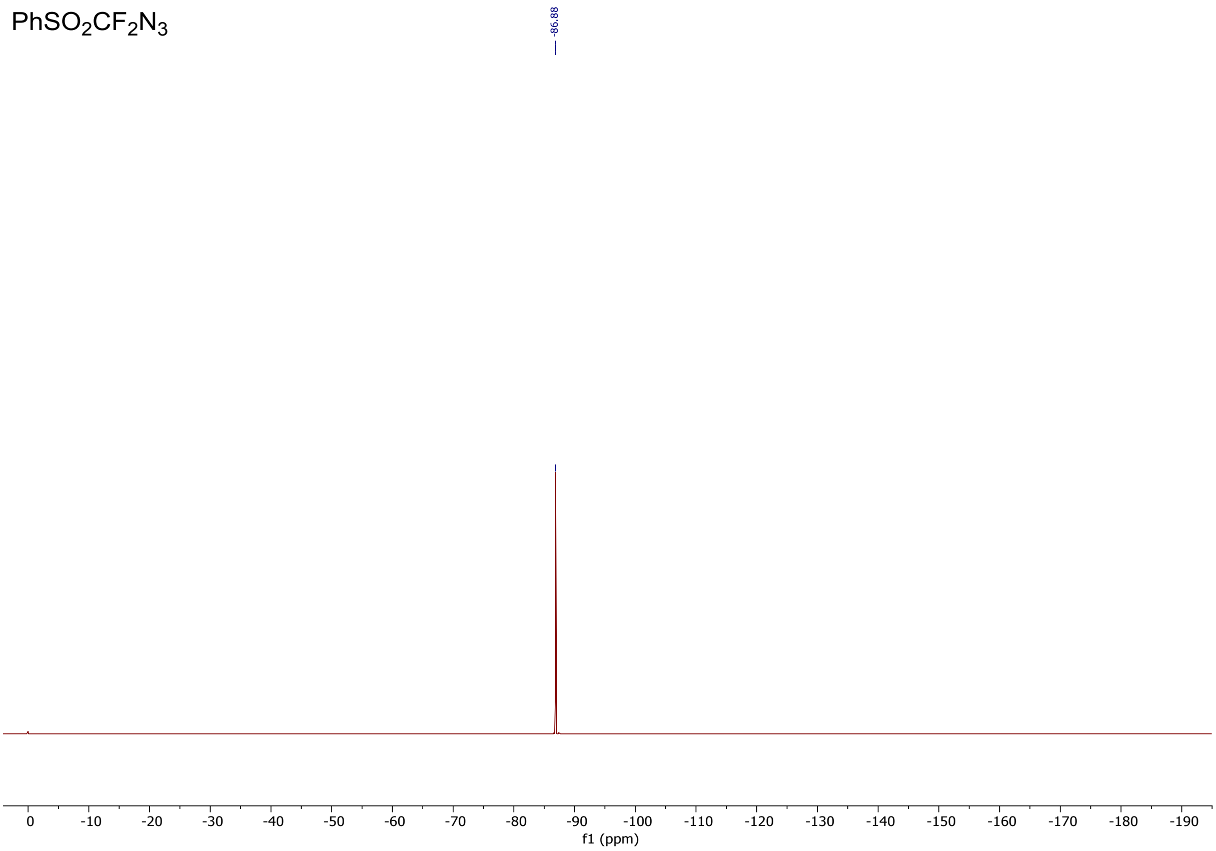

Figure S6. ^{1}H NMR spectrum of **2a** ($CDCl_3$, 400 MHz)

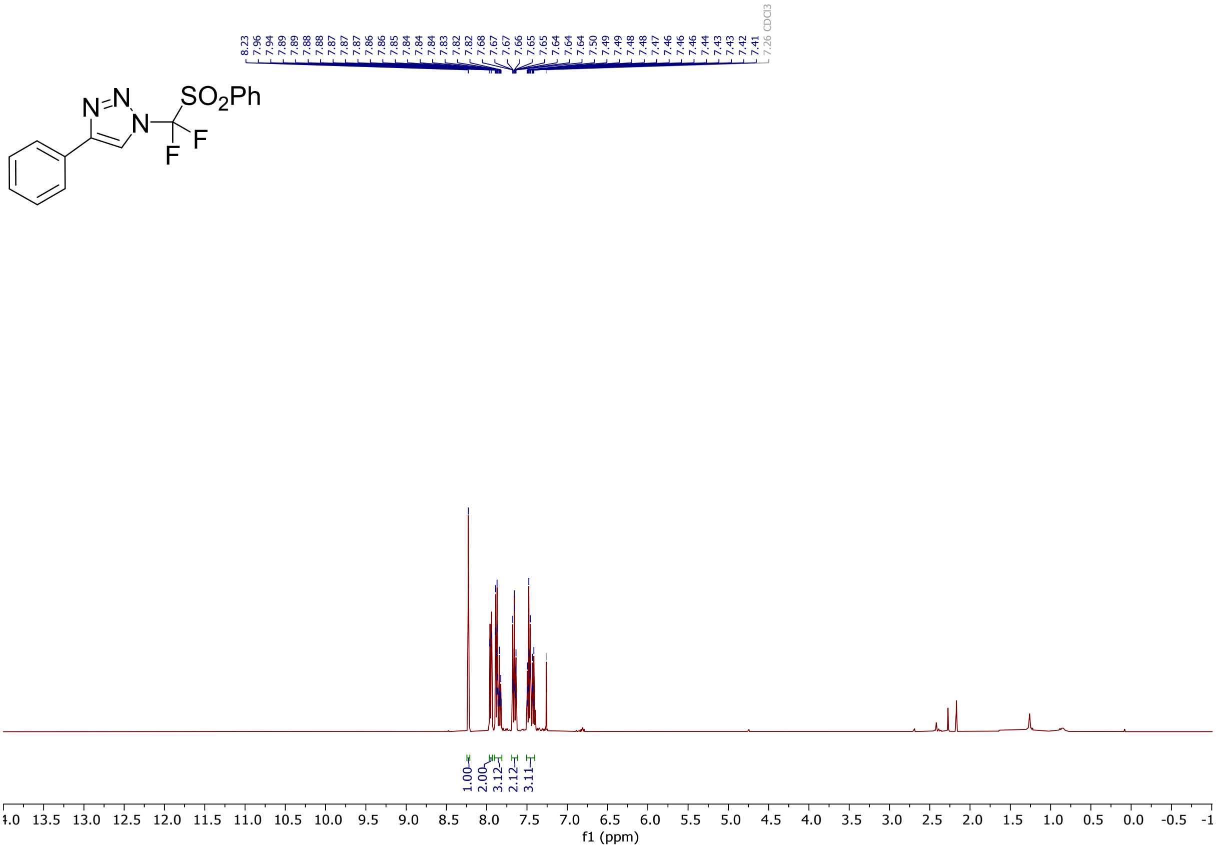

Figure S7. $^{13}C\{^{1}H\}$ NMR spectrum of **2a** ($CDCl_3$, 101 MHz)

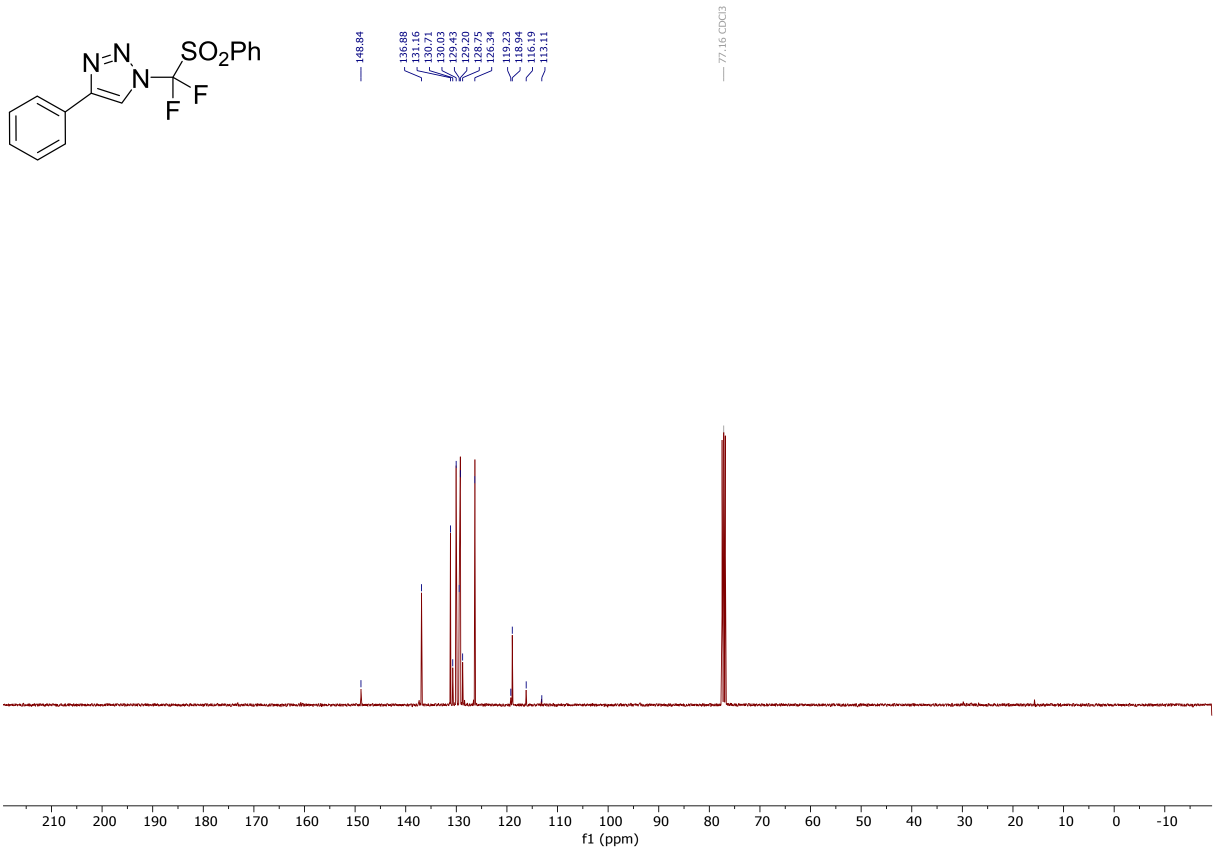

Figure S8. ^{19}F NMR spectrum o f**2a** ($CDCl_3$, 377 MHz)

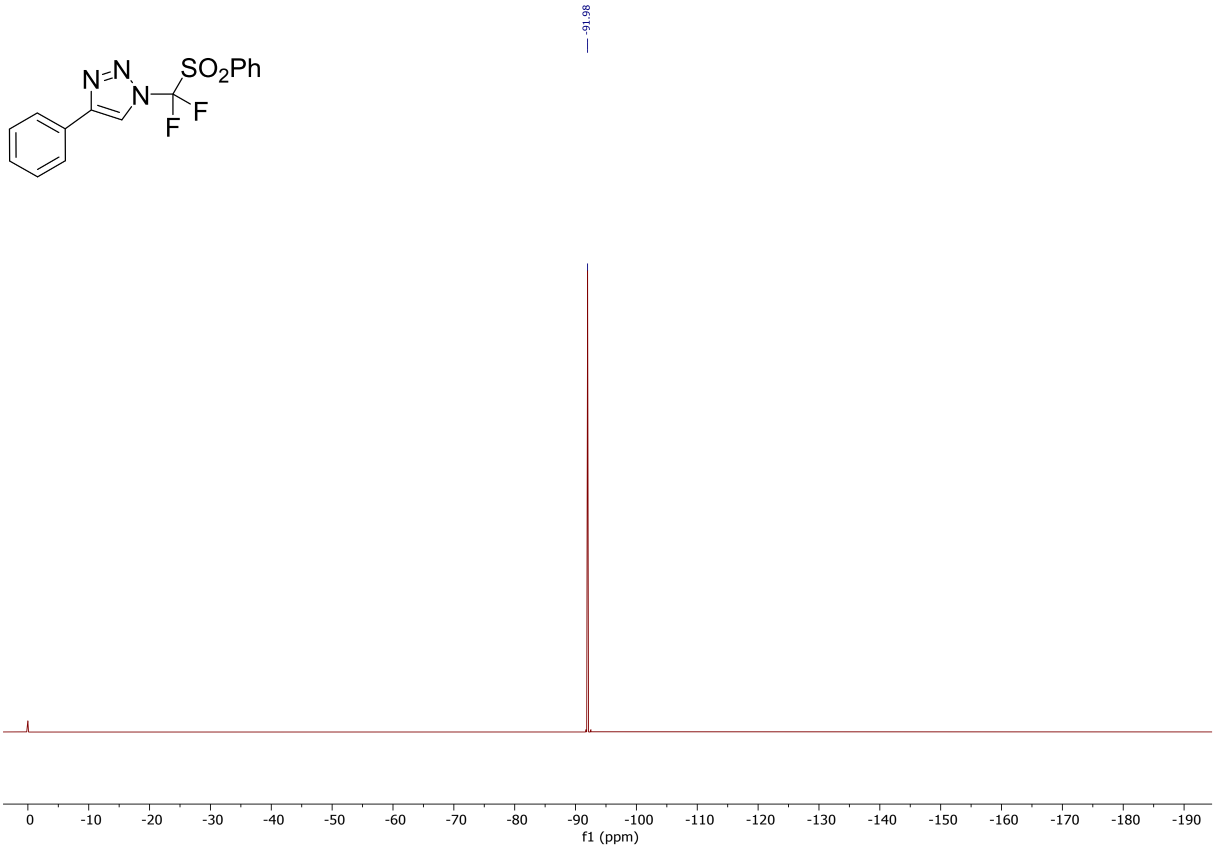

Figure S9. ^{1}H NMR spectrum of **2b** ($CDCl_3$, 400 MHz)

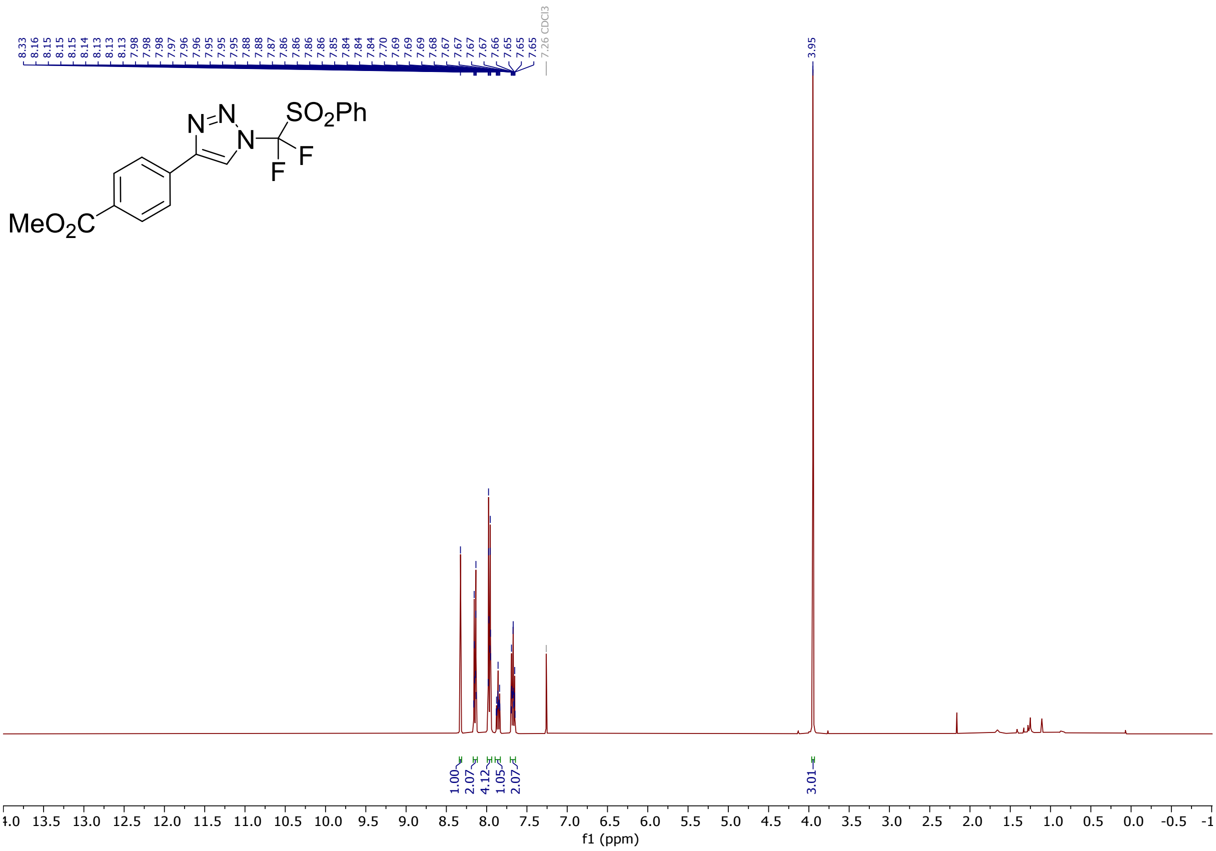

Figure S10. $^{13}C\{^{1}H\}$ NMR spectrum of **2b** ($CDCl_3$, 101 MHz)

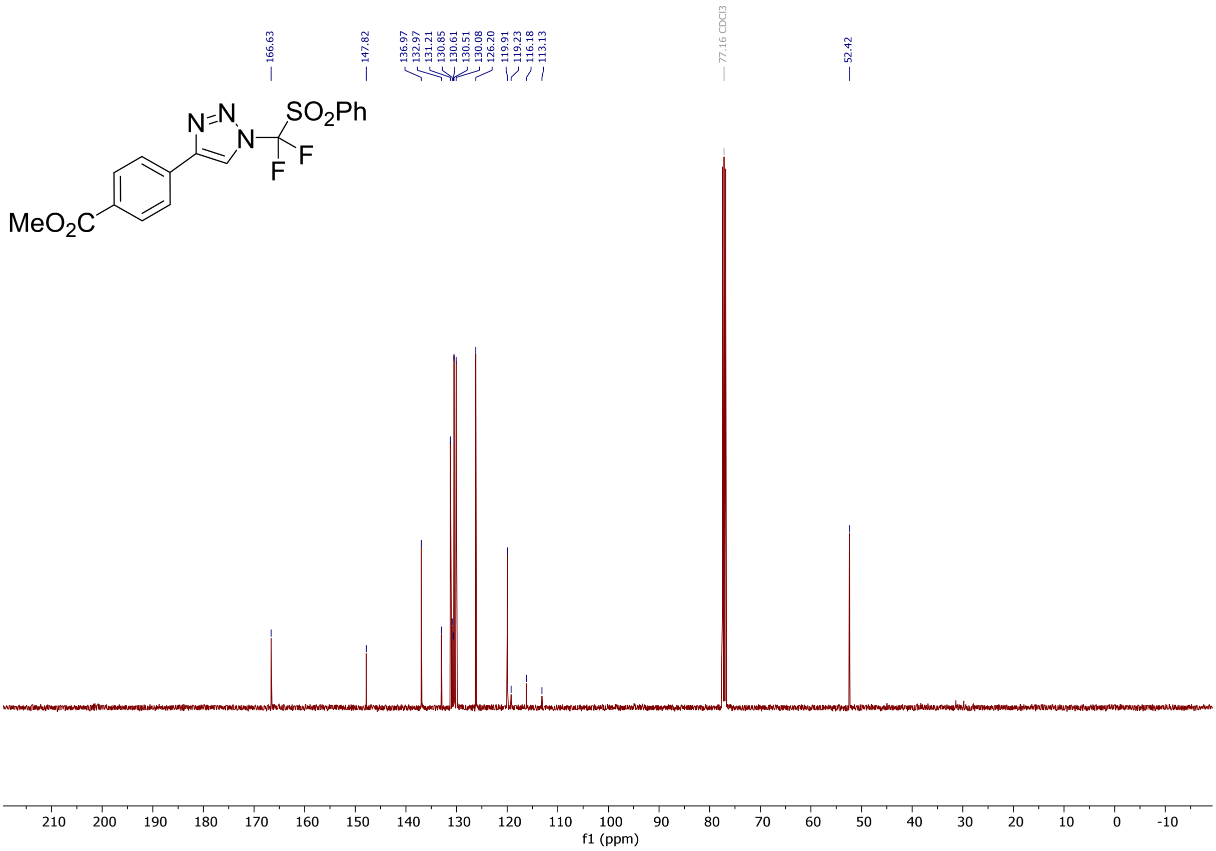

Figure S11. ^{19}F NMR spectrum of **2b** ($CDCl_3$, 377 MHz)

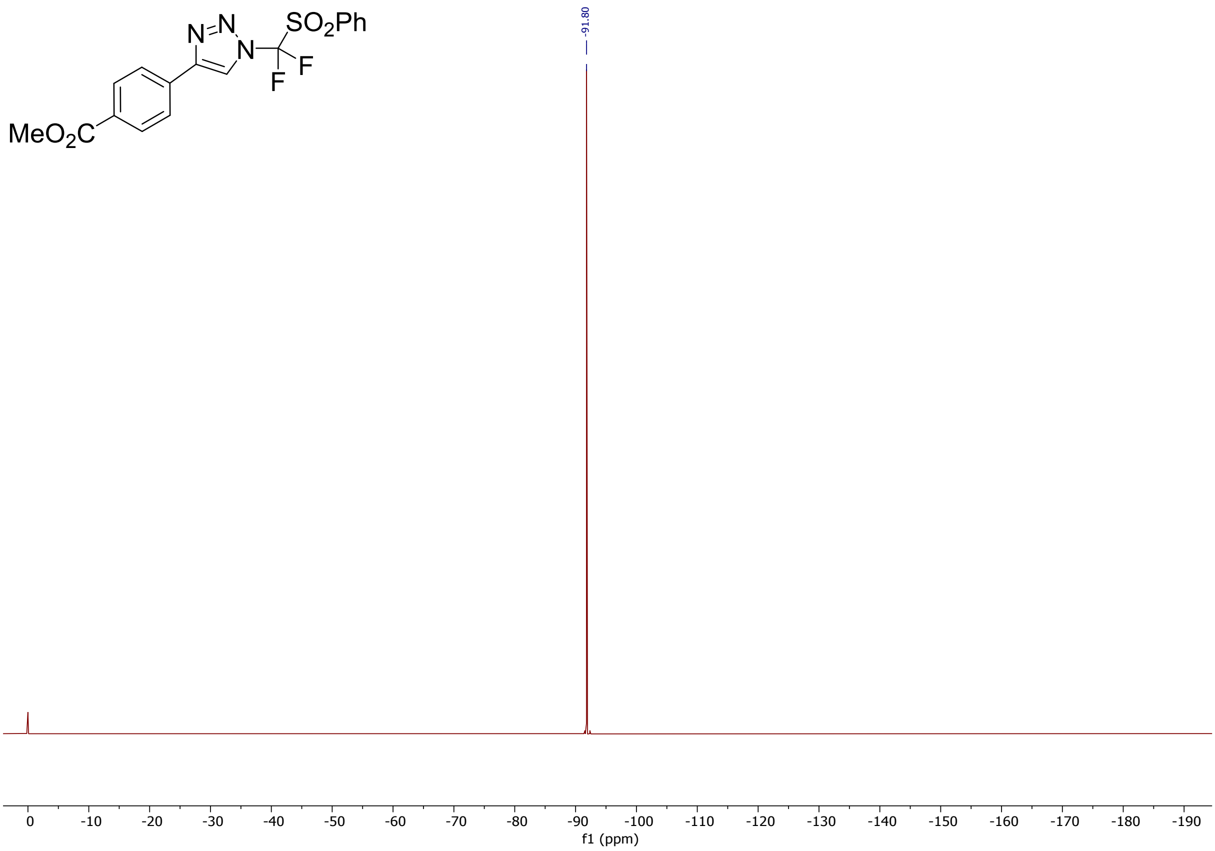

Figure S12. ^{1}H NMR spectrum of **2c** ($CDCl_3$, 400 MHz)

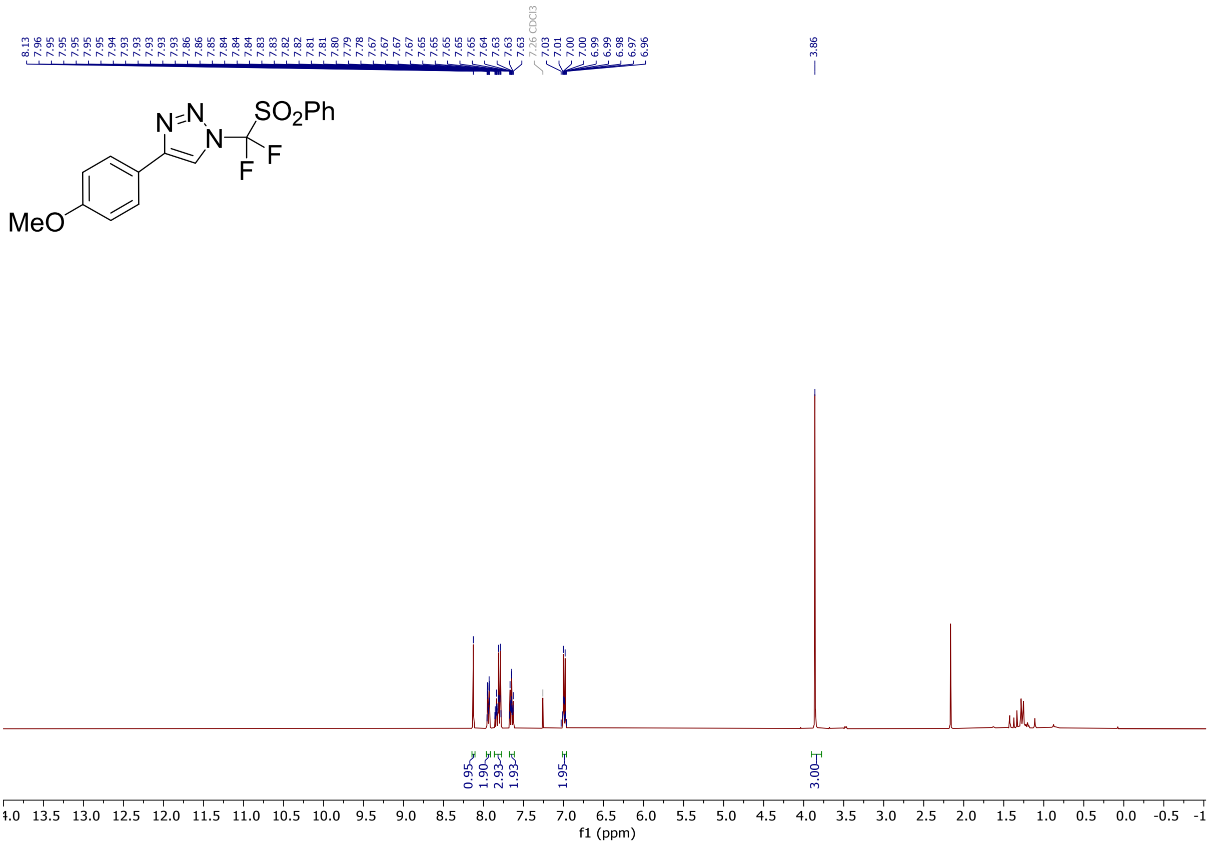

Figure S13. $^{13}C\{^{1}H\}$ NMR spectrum of **2c** ($CDCl_3$, 101 MHz)

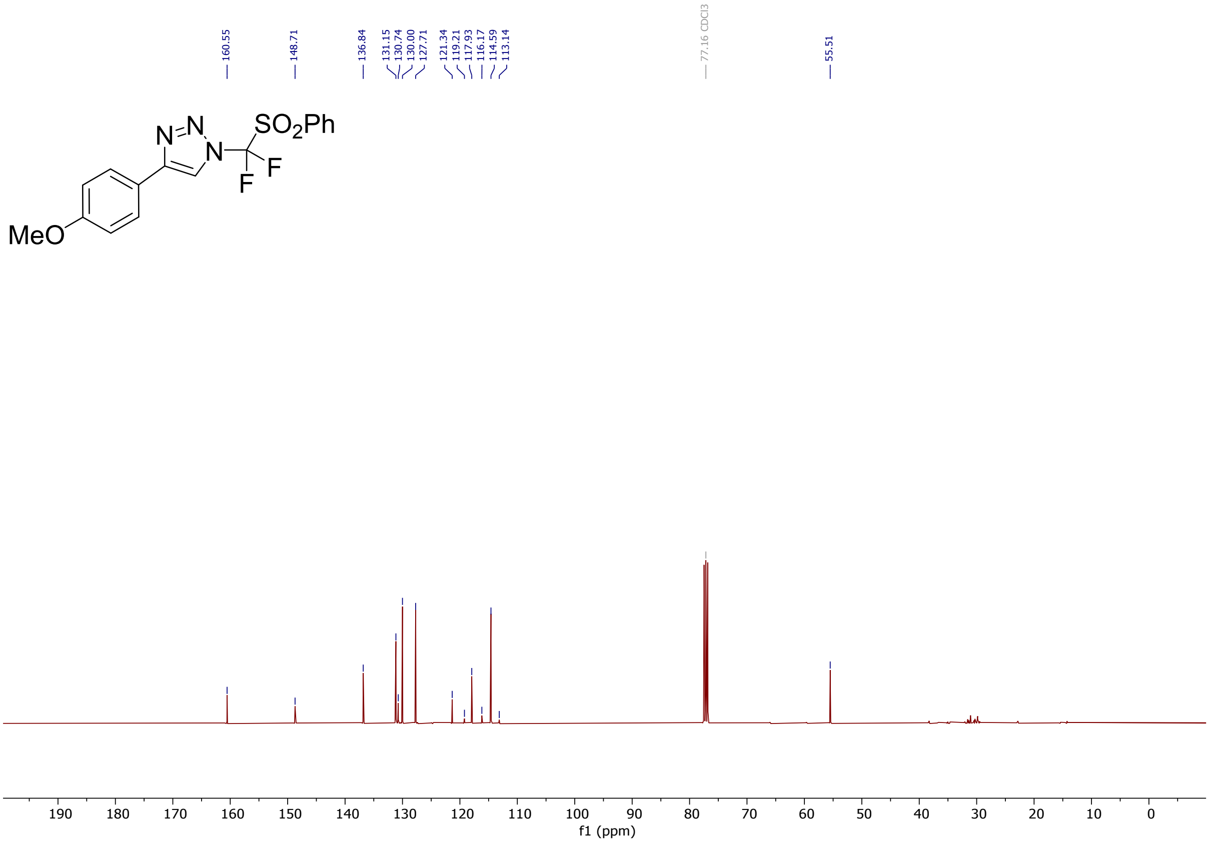

Figure S14. ^{19}F NMR spectrum of **2c** ($CDCl_3$, 377 MHz)

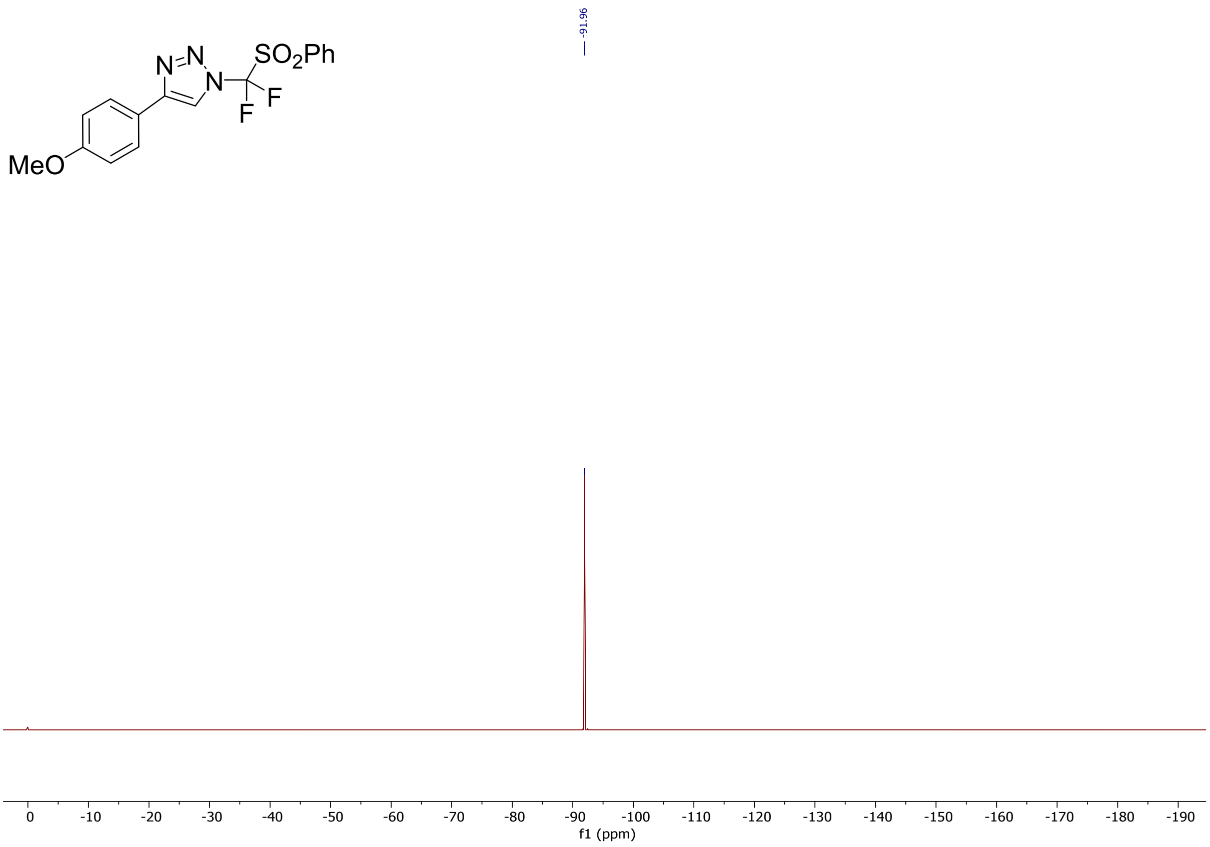

Figure S15. ^{1}H NMR spectrum of **2d** ($CDCl_3$, 400 MHz)

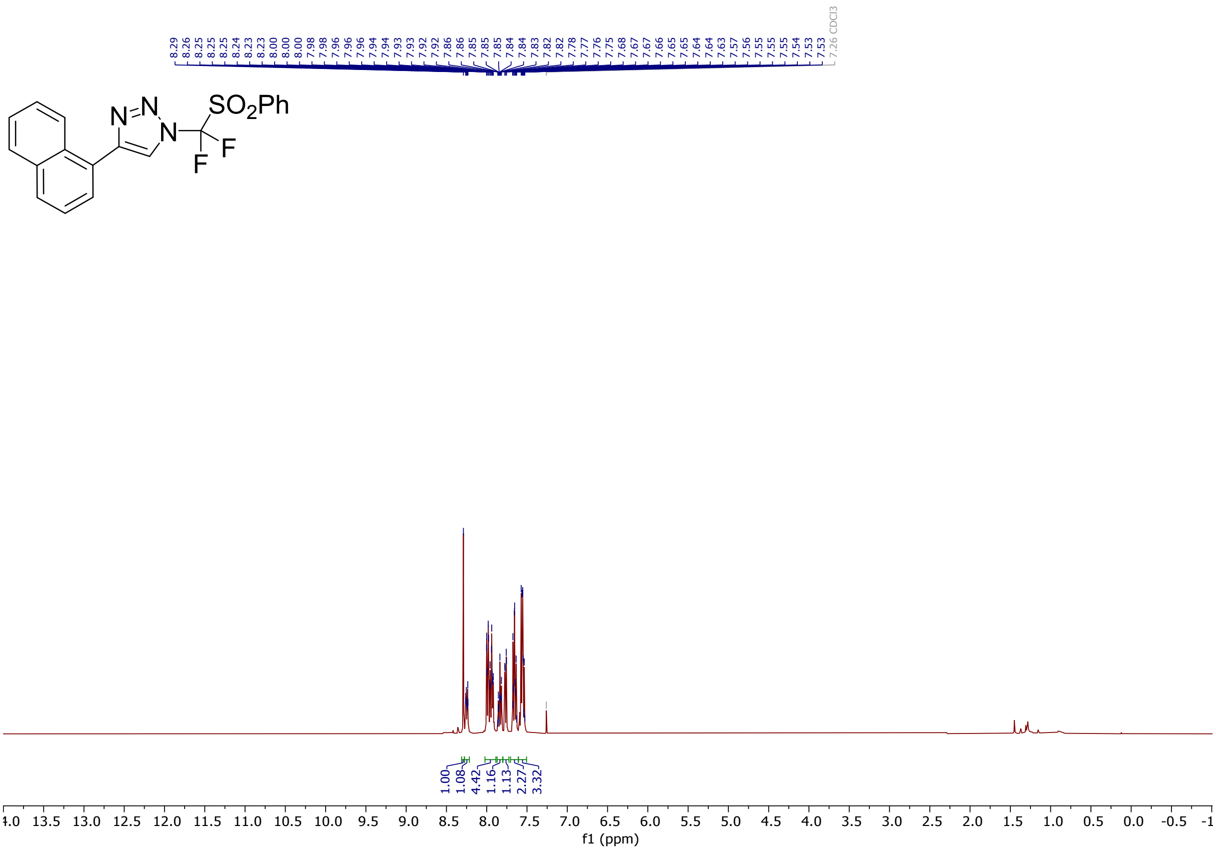

Figure S16. $^{13}C\{^{1}H\}$ NMR spectrum of **2d** ($CDCl_3$, 101 MHz)

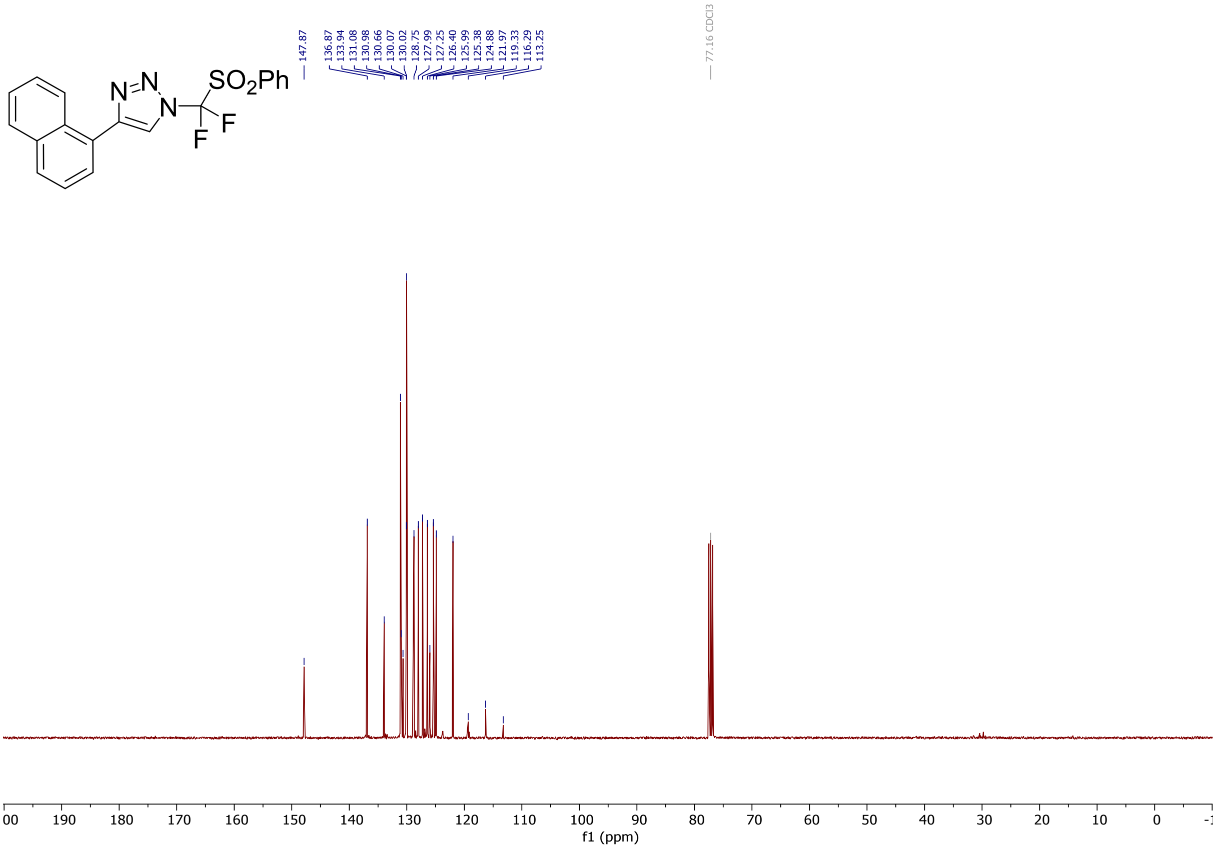

Figure S17. ^{19}F NMR spectrum of **2d** ($CDCl_3$, 377 MHz)

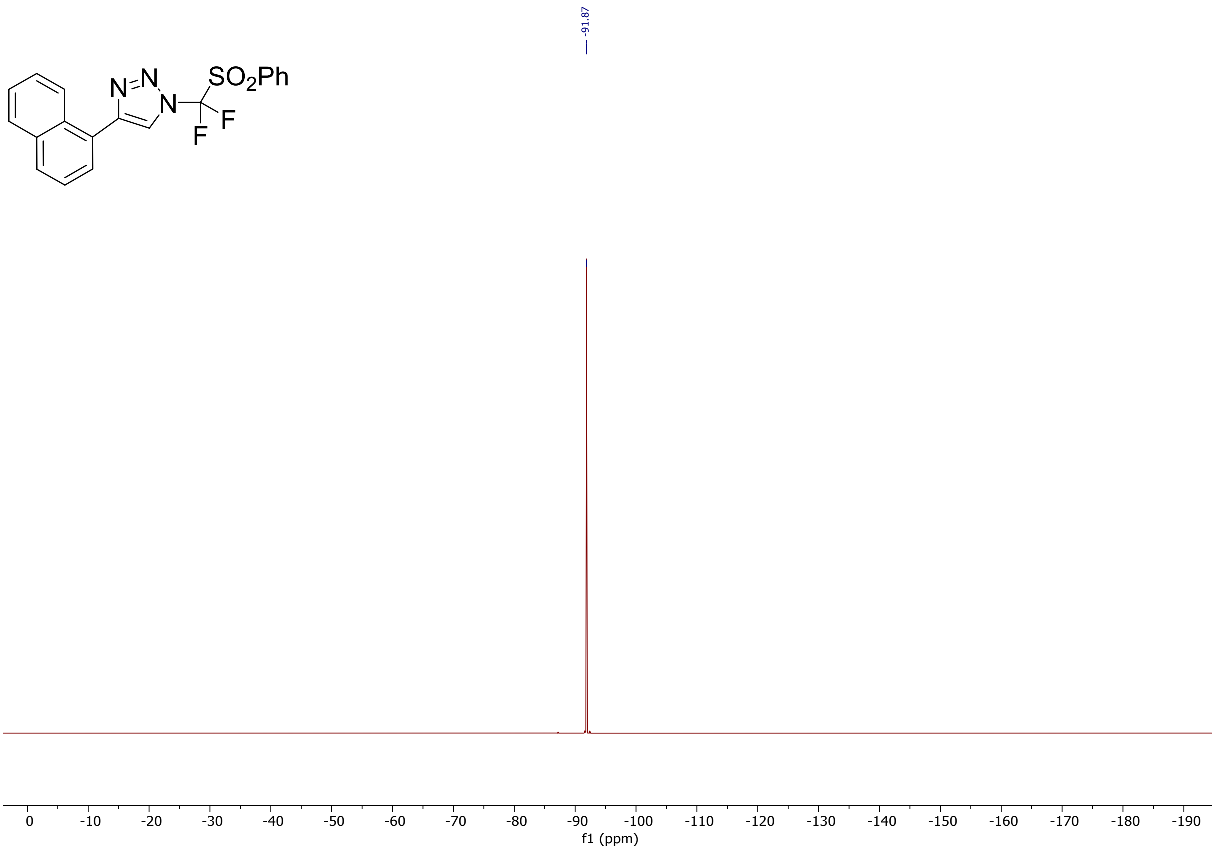

Figure S18. ^{1}H NMR spectrum of **2e** ($CDCl_3$, 400 MHz)

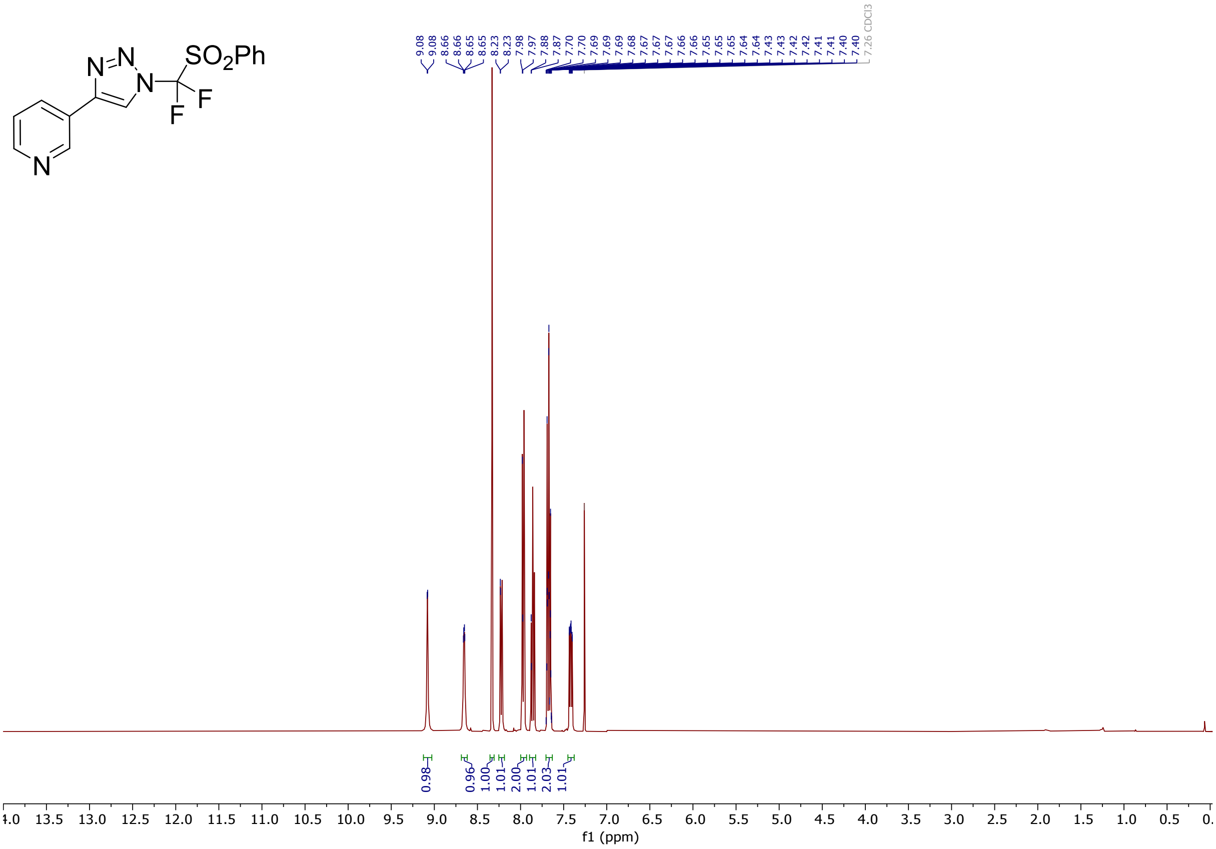

Figure S19. $^{13}C\{^{1}H\}$ NMR spectrum of **2e** ($CDCl_3$, 101 MHz)

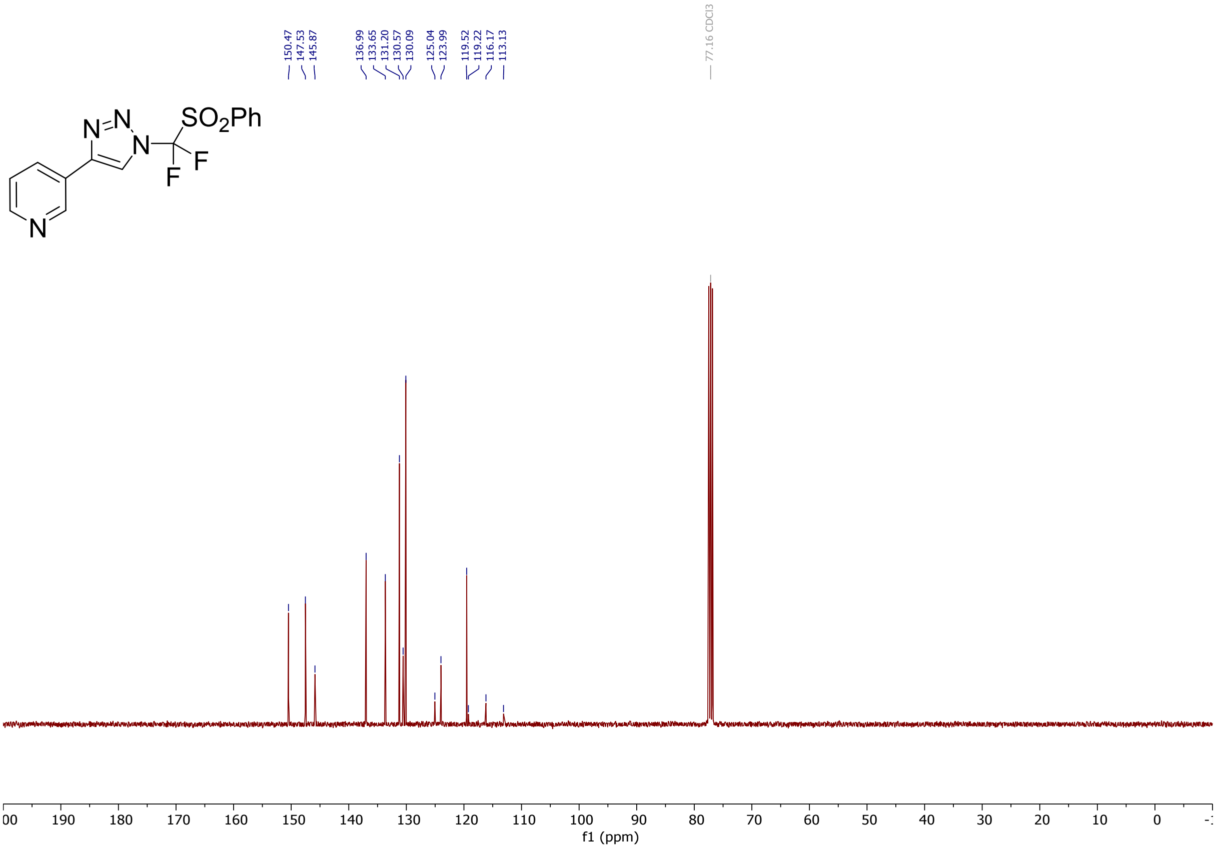

Figure S20. ^{19}F NMR spectrum of **2e** ($CDCl_3$, 377 MHz)

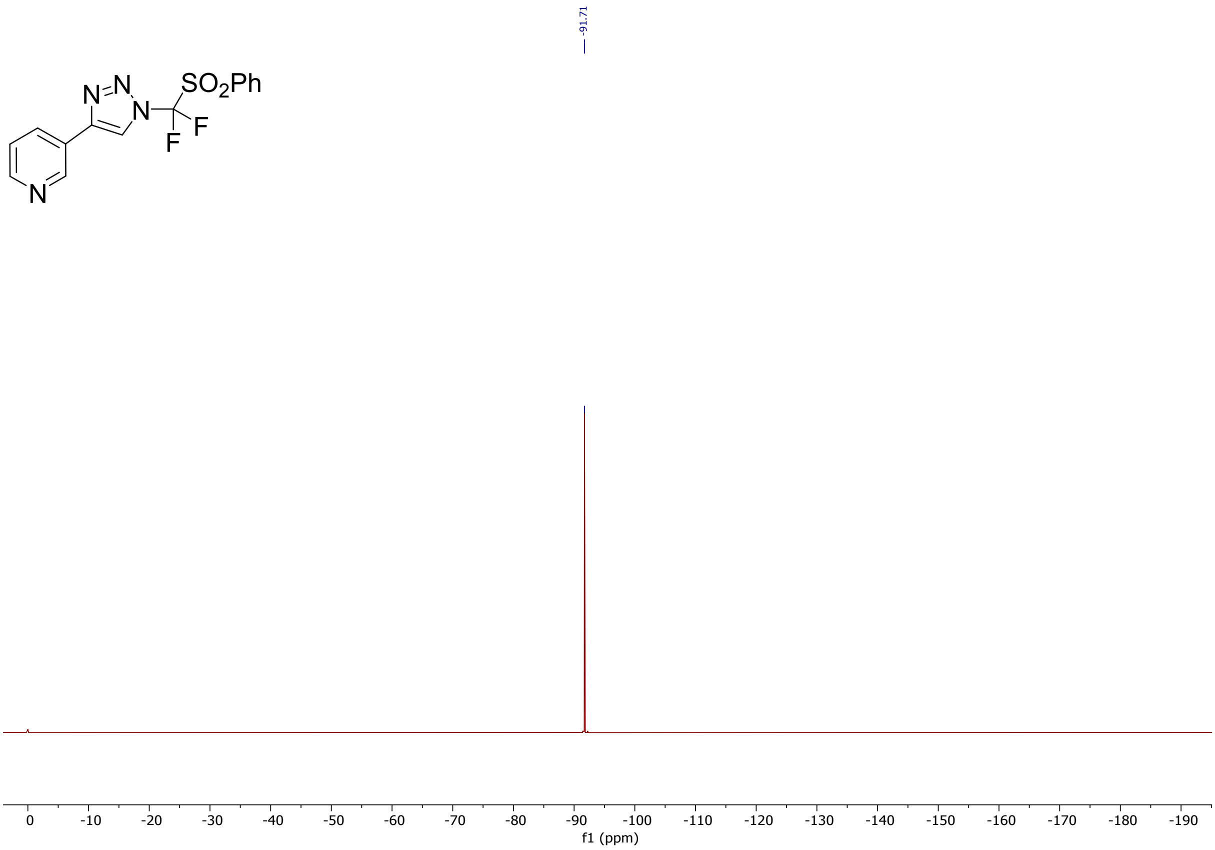

Figure S21. ^{1}H NMR spectrum of **2f** ($CDCl_3$, 400 MHz)

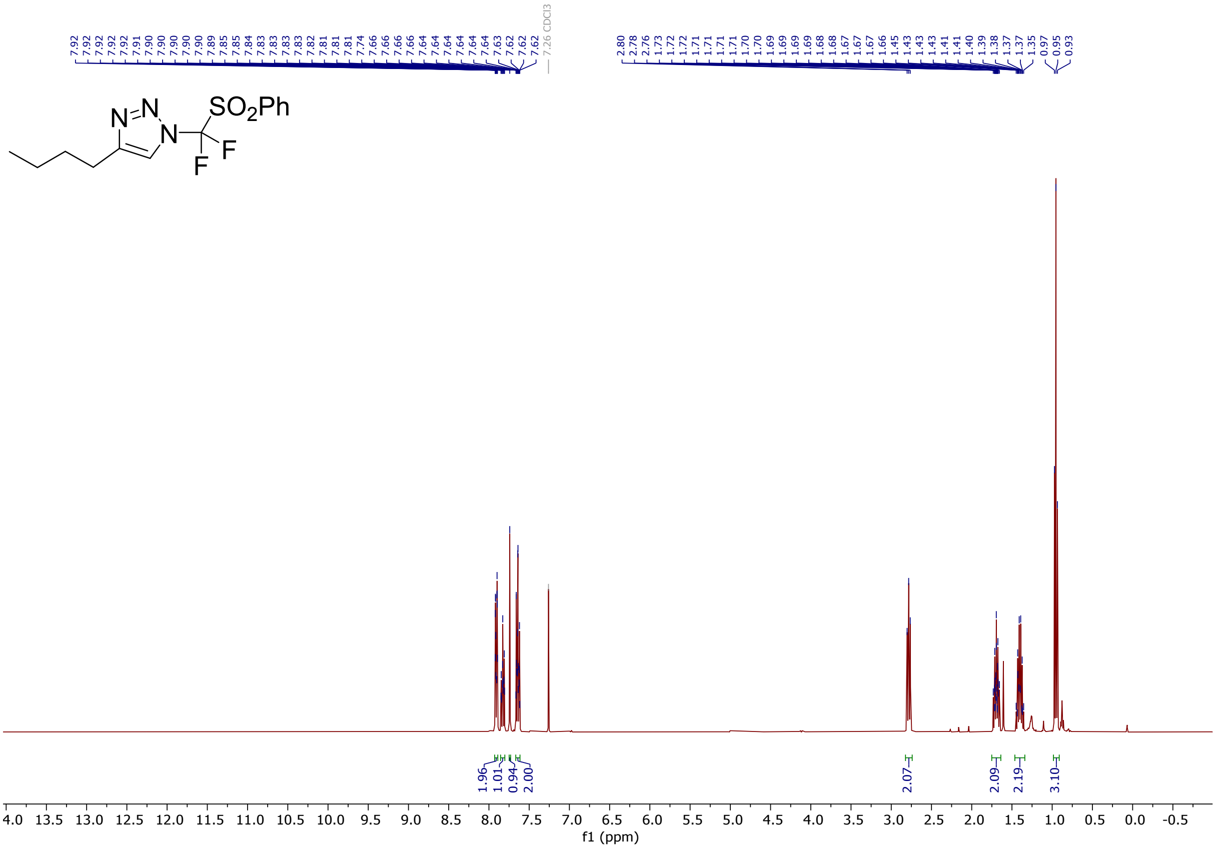

Figure S22. $^{13}C\{^1H\}$ NMR spectrum of **2f** ($CDCl_3$, 101 MHz)

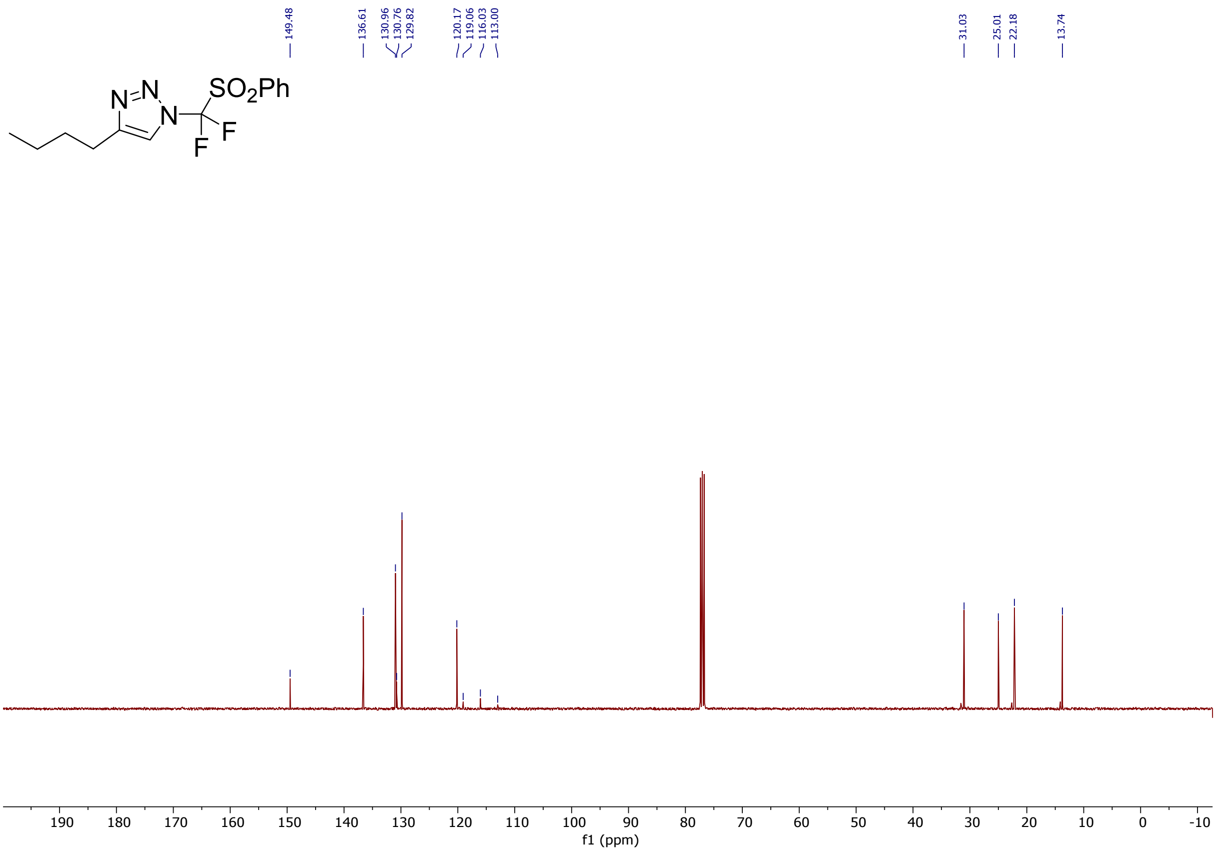

Figure S23. ^{19}F NMR spectrum of **2f** ($CDCl_3$, 377 MHz)

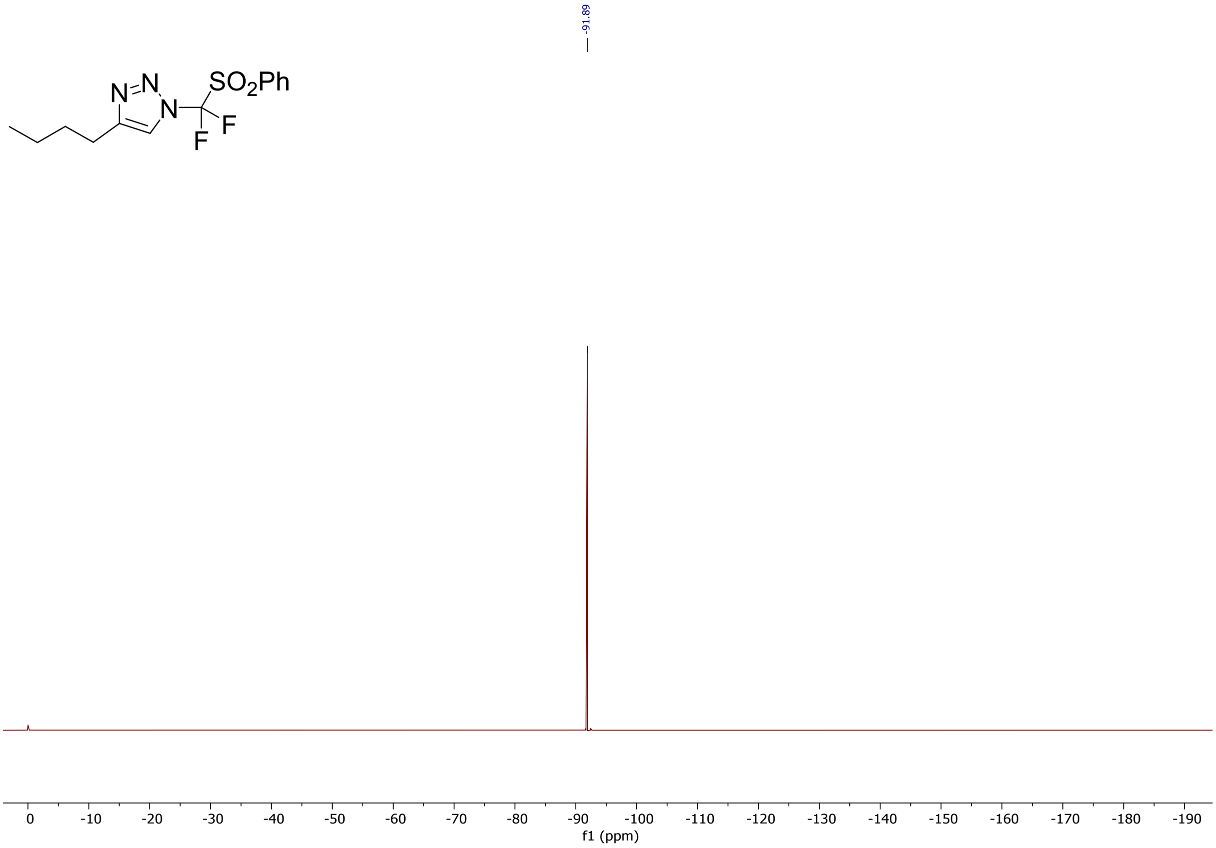

Figure S24. ^{1}H NMR spectrum of **2g** (DMSO-D$_6$, 400 MHz)

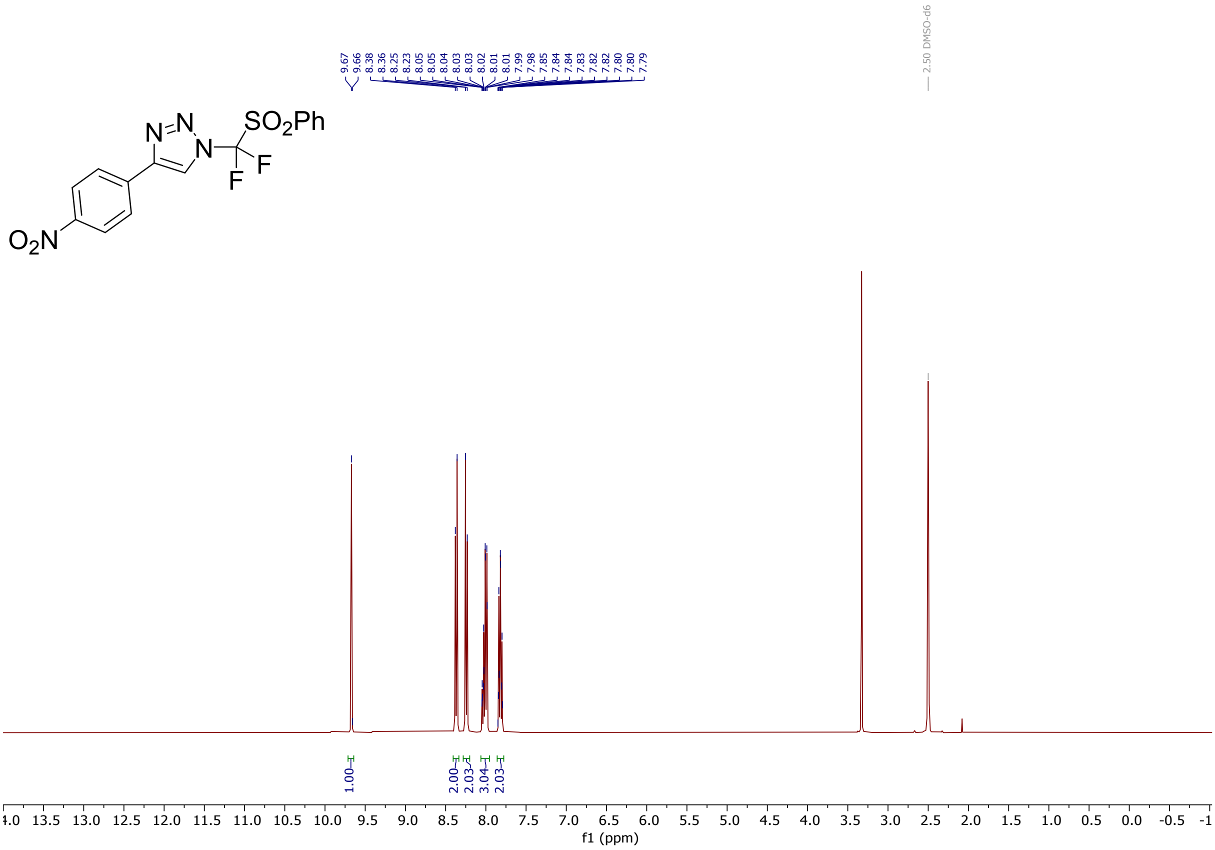

Figure S25. $^{13}C\{^{1}H\}$ NMR spectrum of **2g** (DMSO-D_6, 101 MHz)

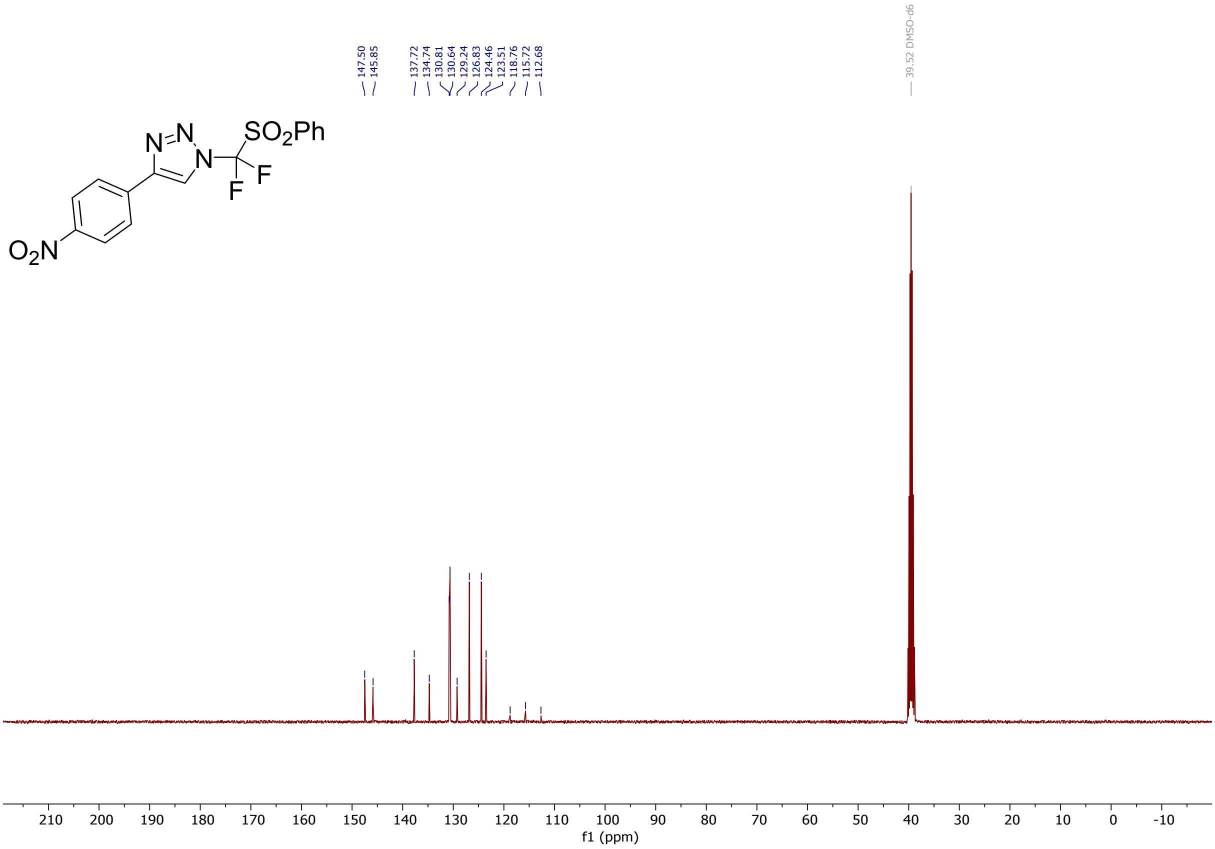

Figure S26. ^{19}F NMR spectrum of **2g** (DMSO-D_6, 377 MHz)

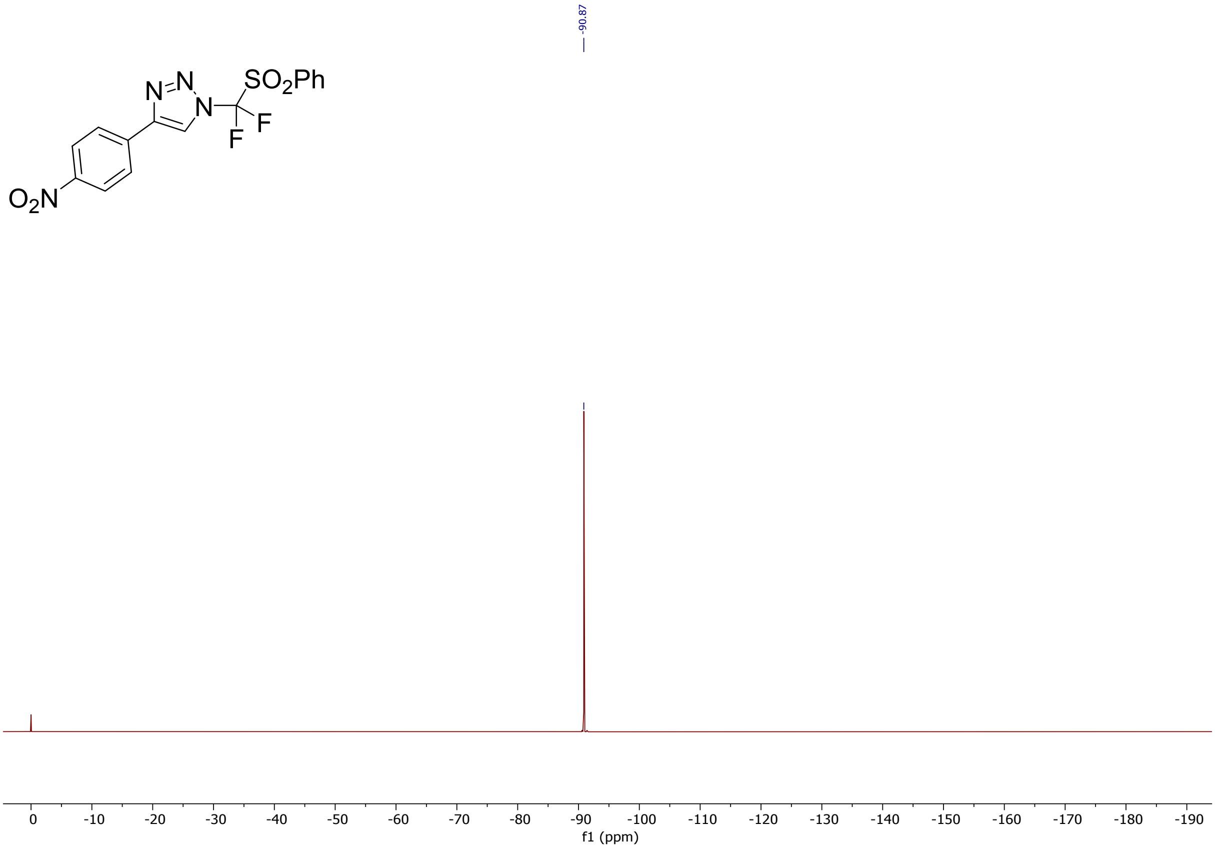

Figure S27. ^{1}H NMR spectrum of **2h** ($CDCl_3$, 400 MHz)

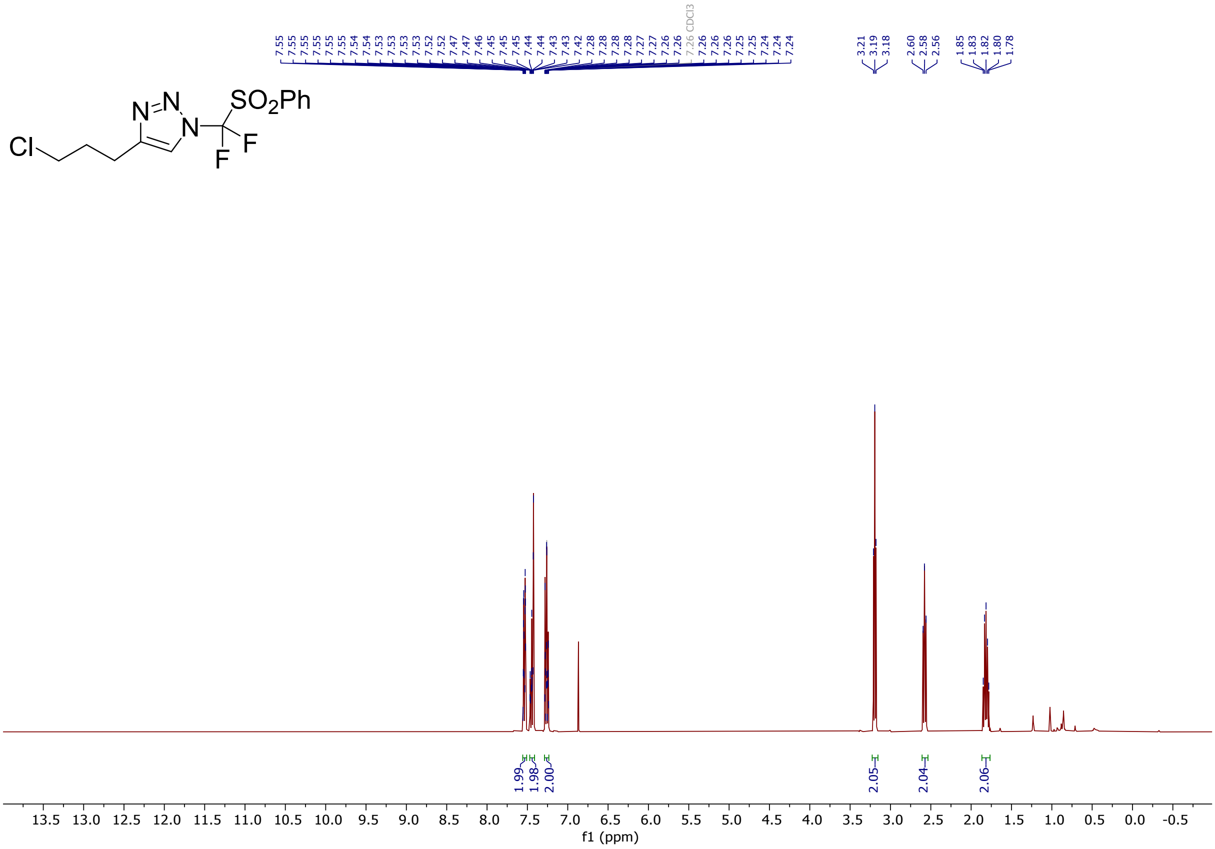

Figure S28. $^{13}C\{^{1}H\}$ NMR spectrum of **2h** ($CDCl_3$, 101 MHz)

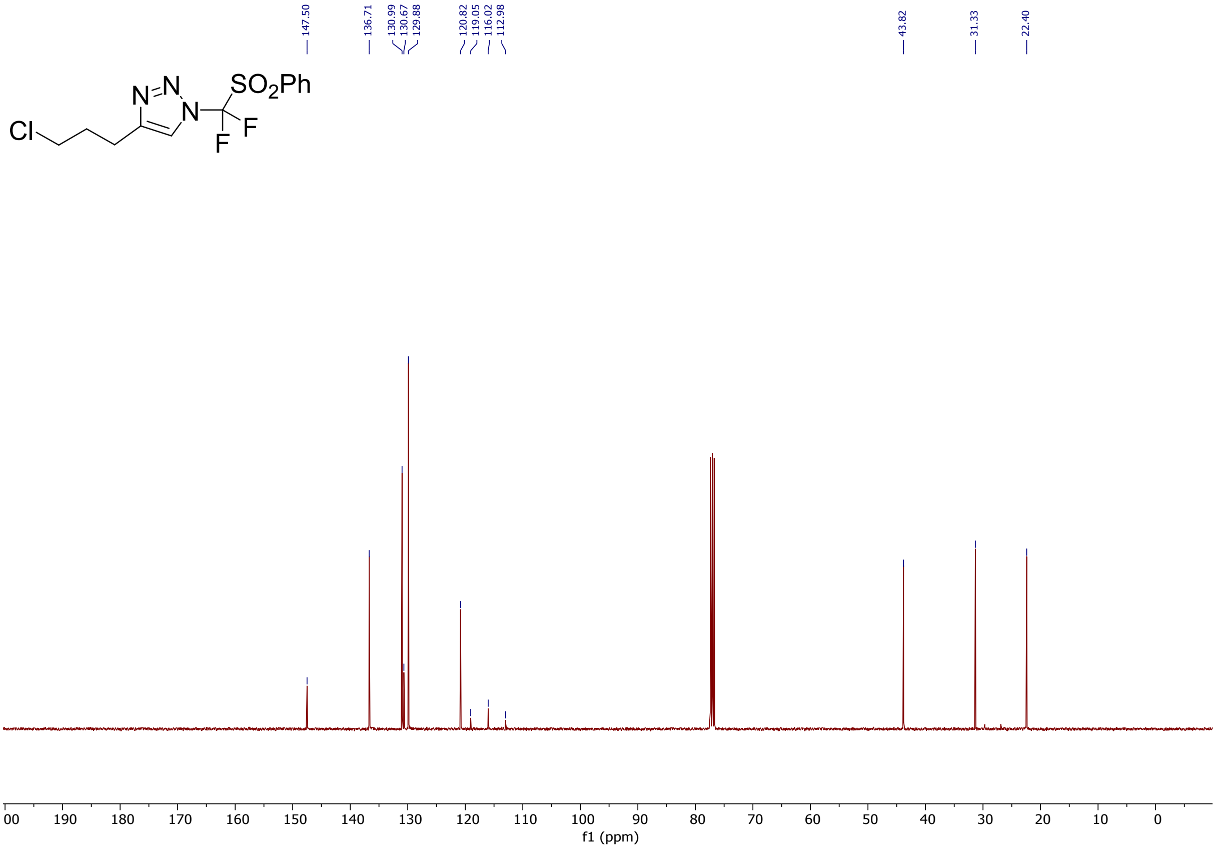

Figure S29. ^{19}F NMR spectrum of **2h** ($CDCl_3$, 377 MHz)

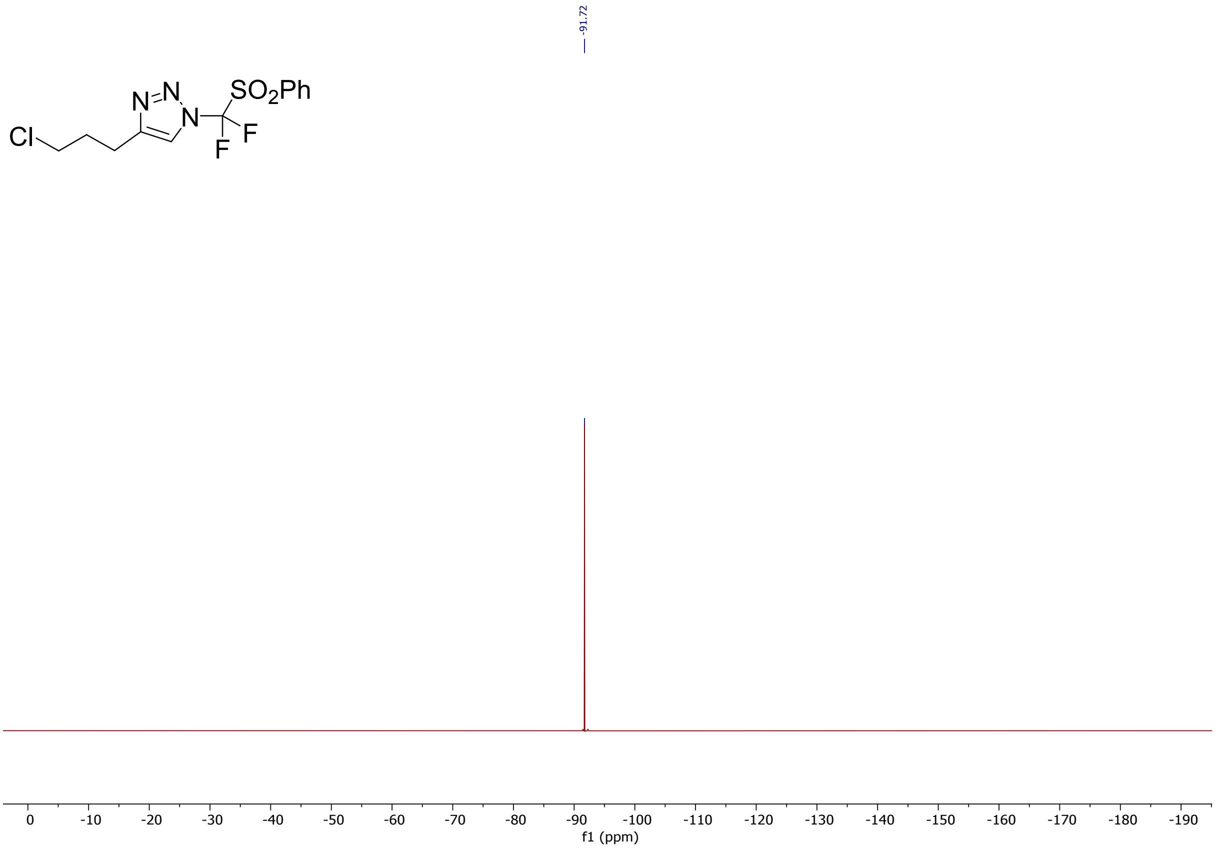

Figure S30. ^{1}H NMR spectrum of **2i** ($CDCl_3$, 400 MHz)

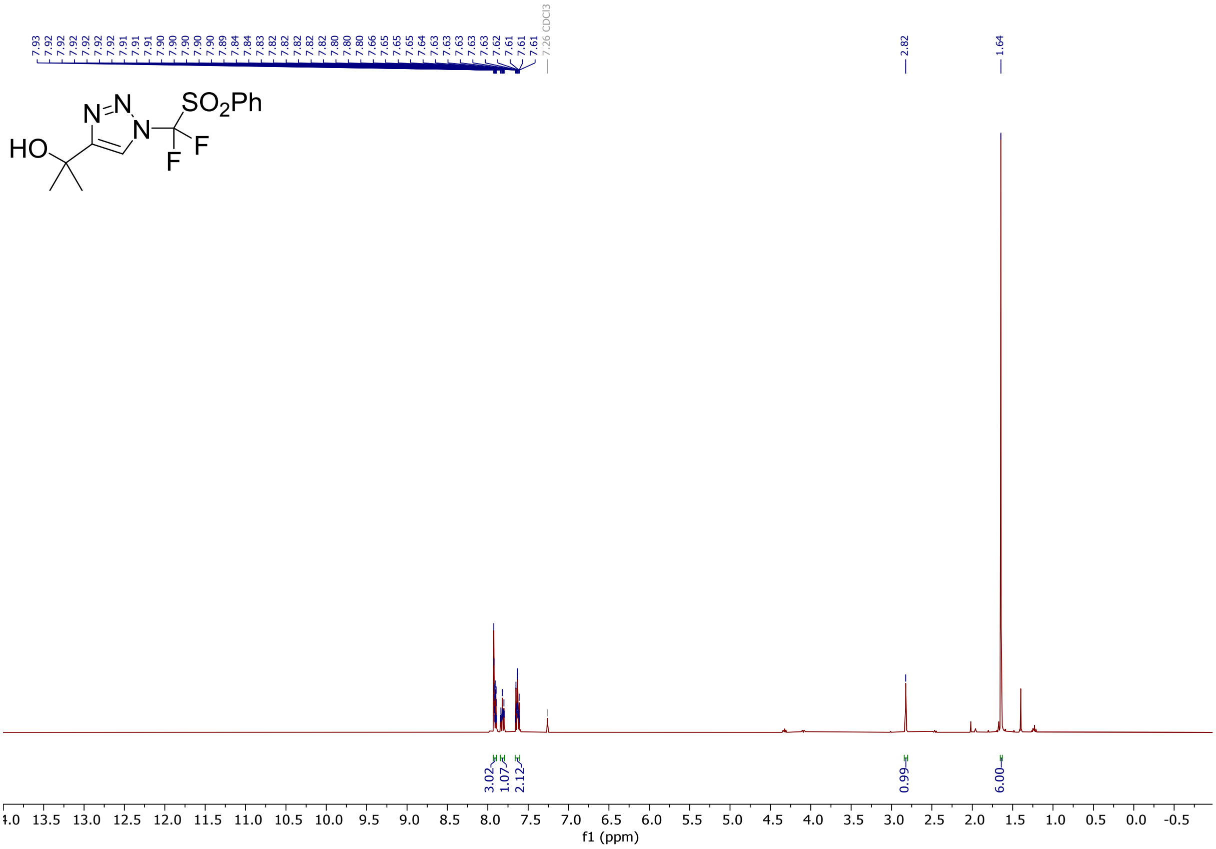

Figure S31. $^{13}C\{^{1}H\}$ NMR spectrum of **2i** ($CDCl_3$, 101 MHz)

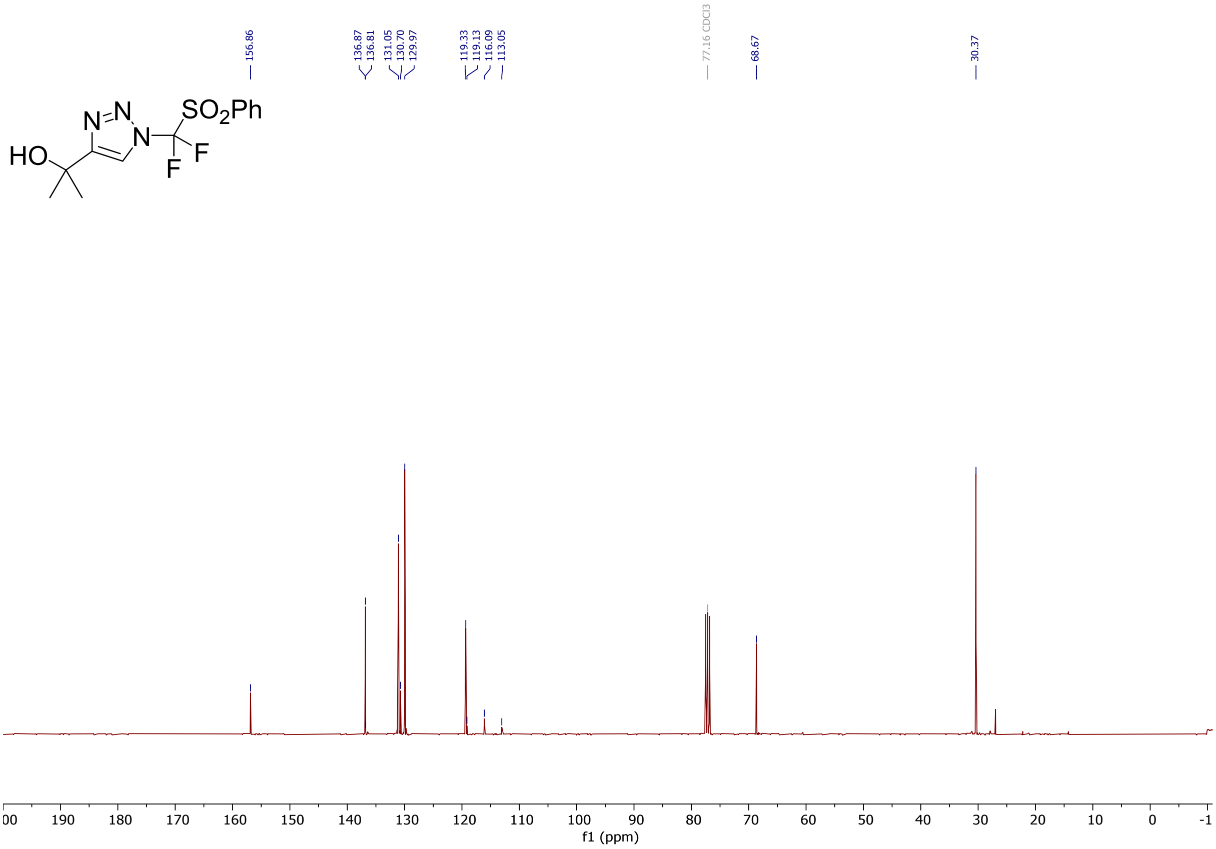

Figure S32. ^{19}F NMR spectrum of **2i** ($CDCl_3$, 377 MHz)

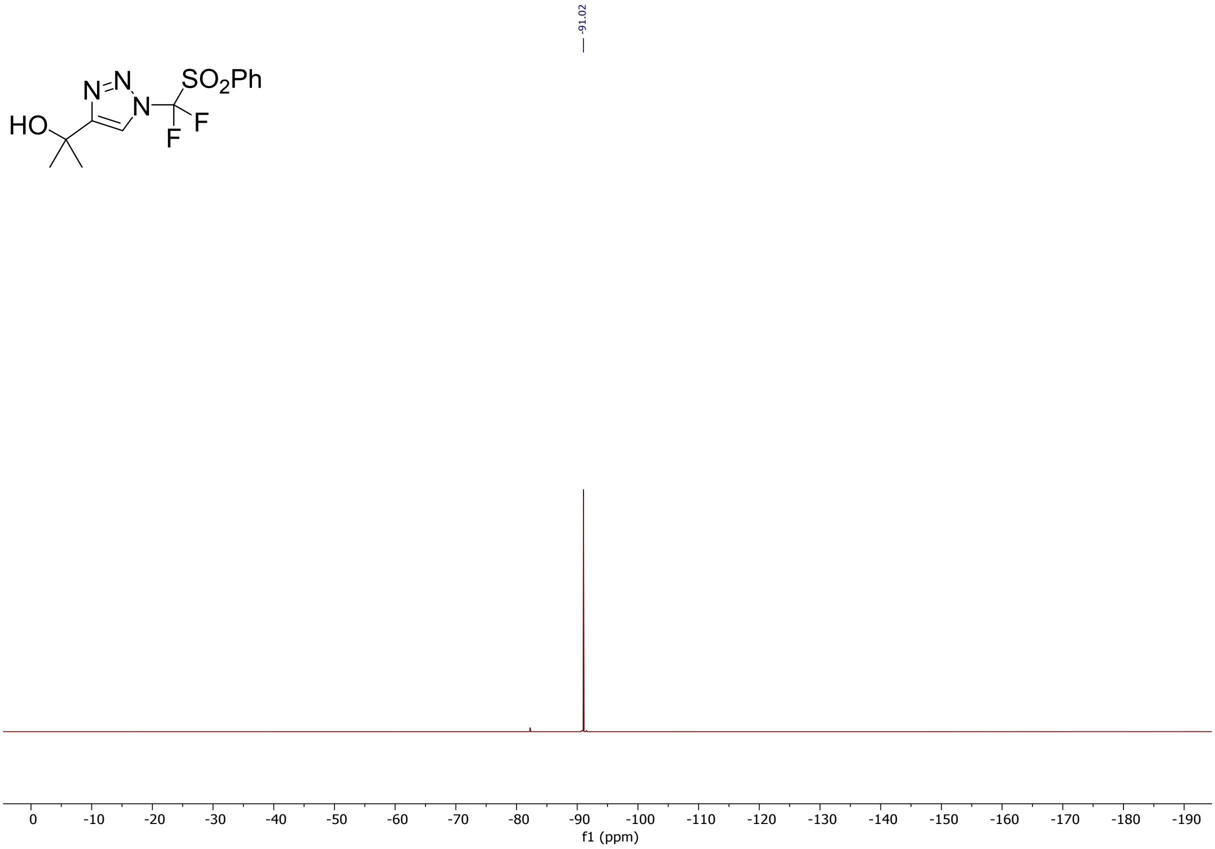

Figure S33. ^{1}H NMR spectrum of **2j** ($CDCl_3$, 400 MHz)

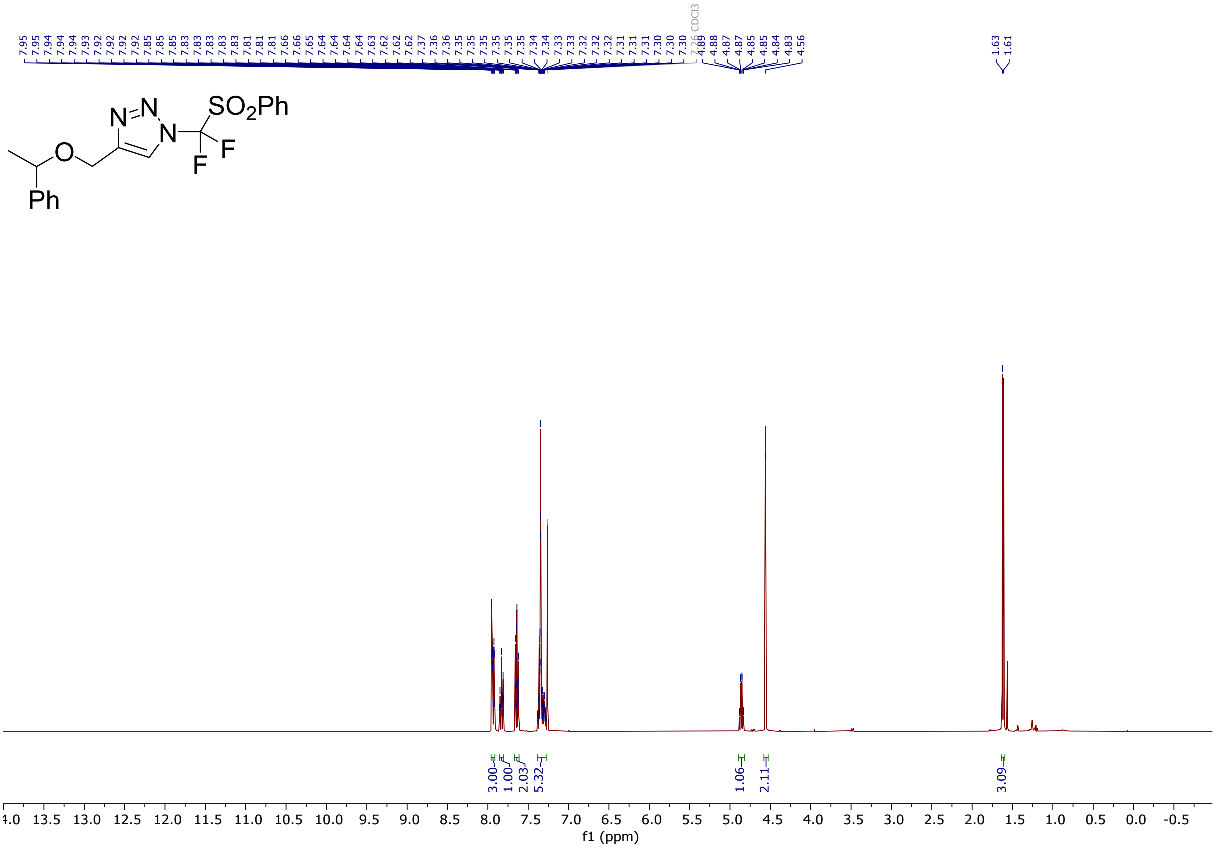

Figure S34. $^{13}C\{^{1}H\}$ NMR spectrum of **2j** ($CDCl_3$, 101 MHz)

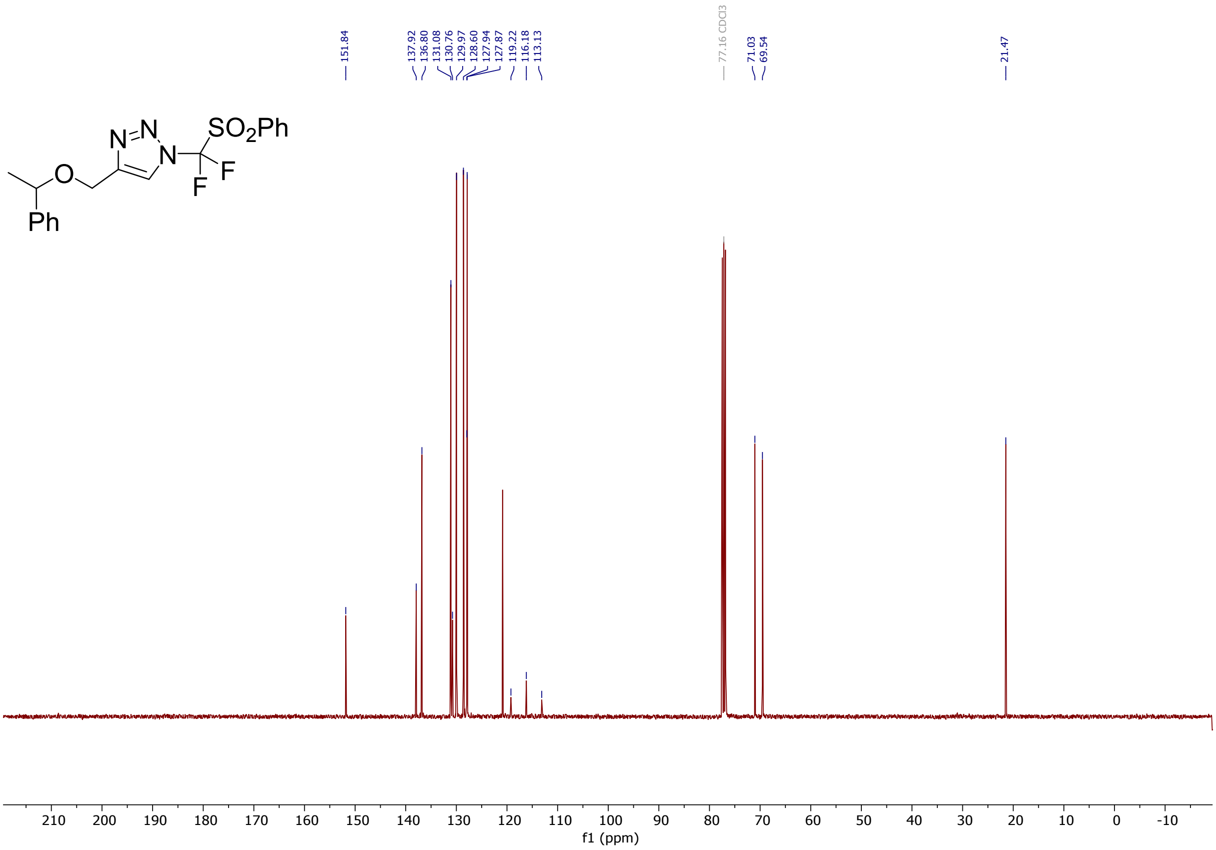

Figure S35. ^{19}F NMR spectrum of **2j** ($CDCl_3$, 377 MHz)

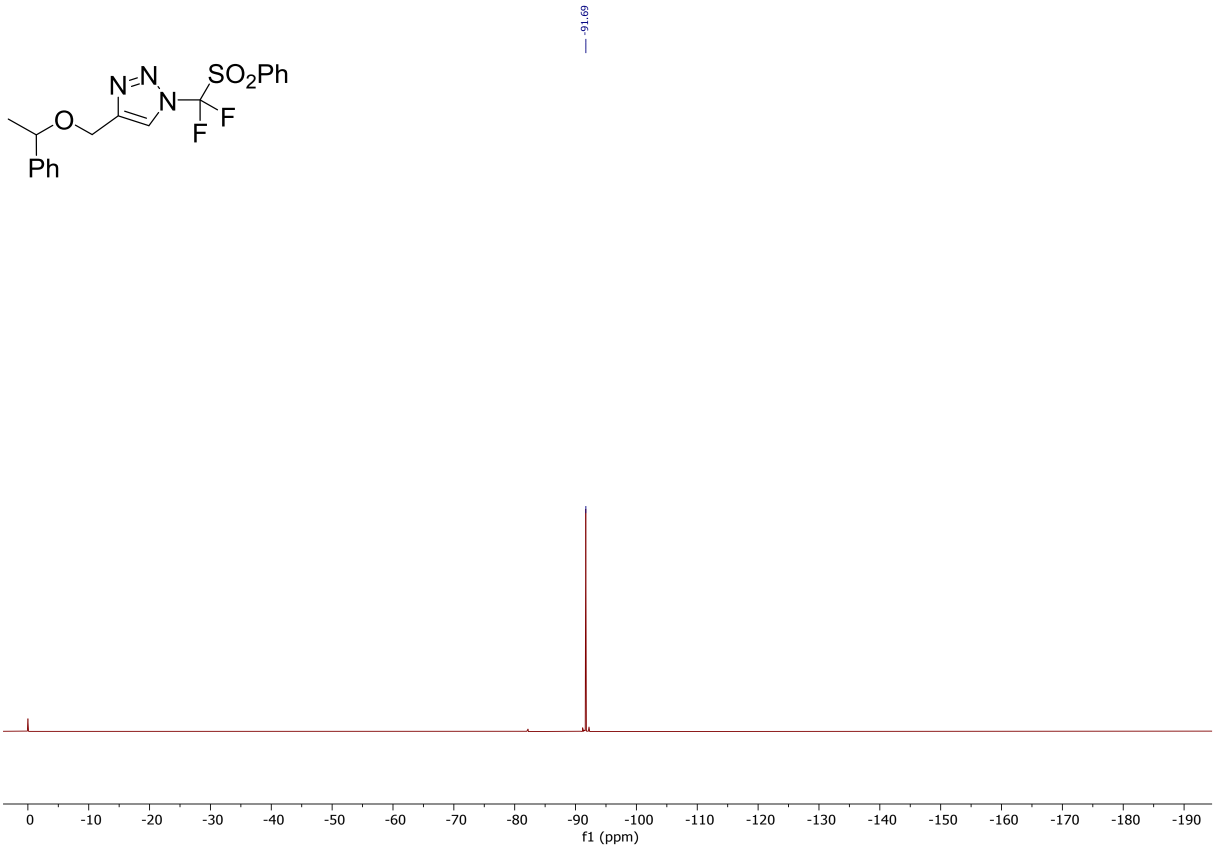

Figure S36. ^{1}H NMR spectrum of **2k** ($CDCl_3$, 400 MHz)

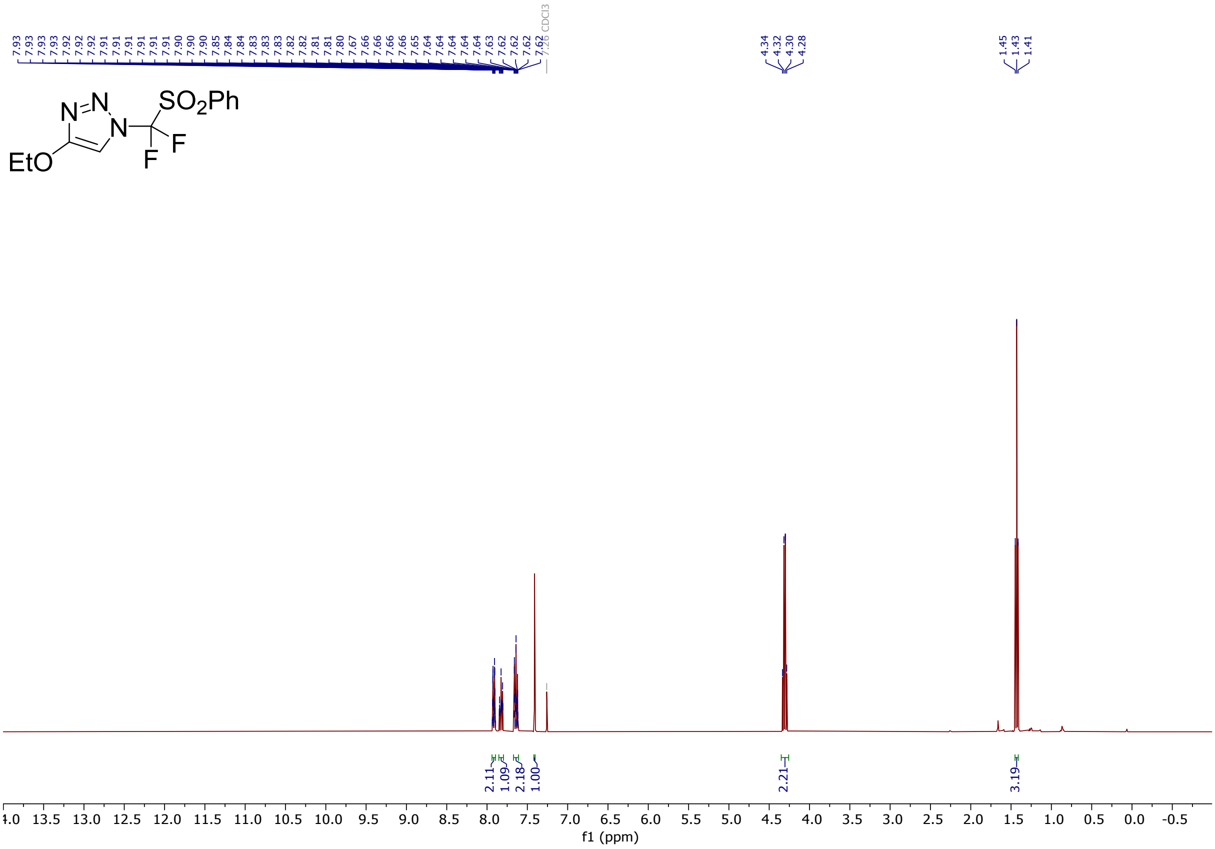

Figure S37. $^{13}C\{^{1}H\}$ NMR spectrum of **2k** ($CDCl_3$, 101 MHz)

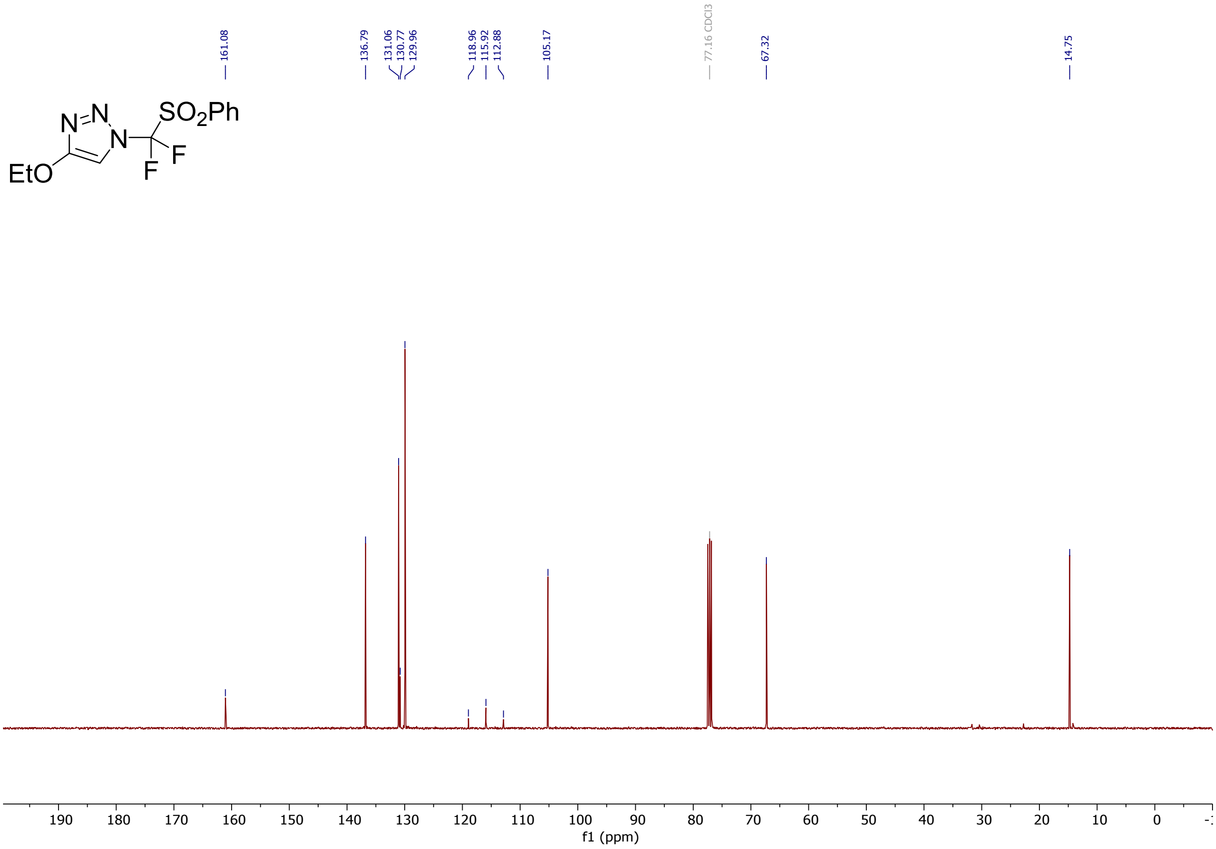

Figure S38. ^{19}F NMR spectrum of **2k** ($CDCl_3$, 377 MHz)

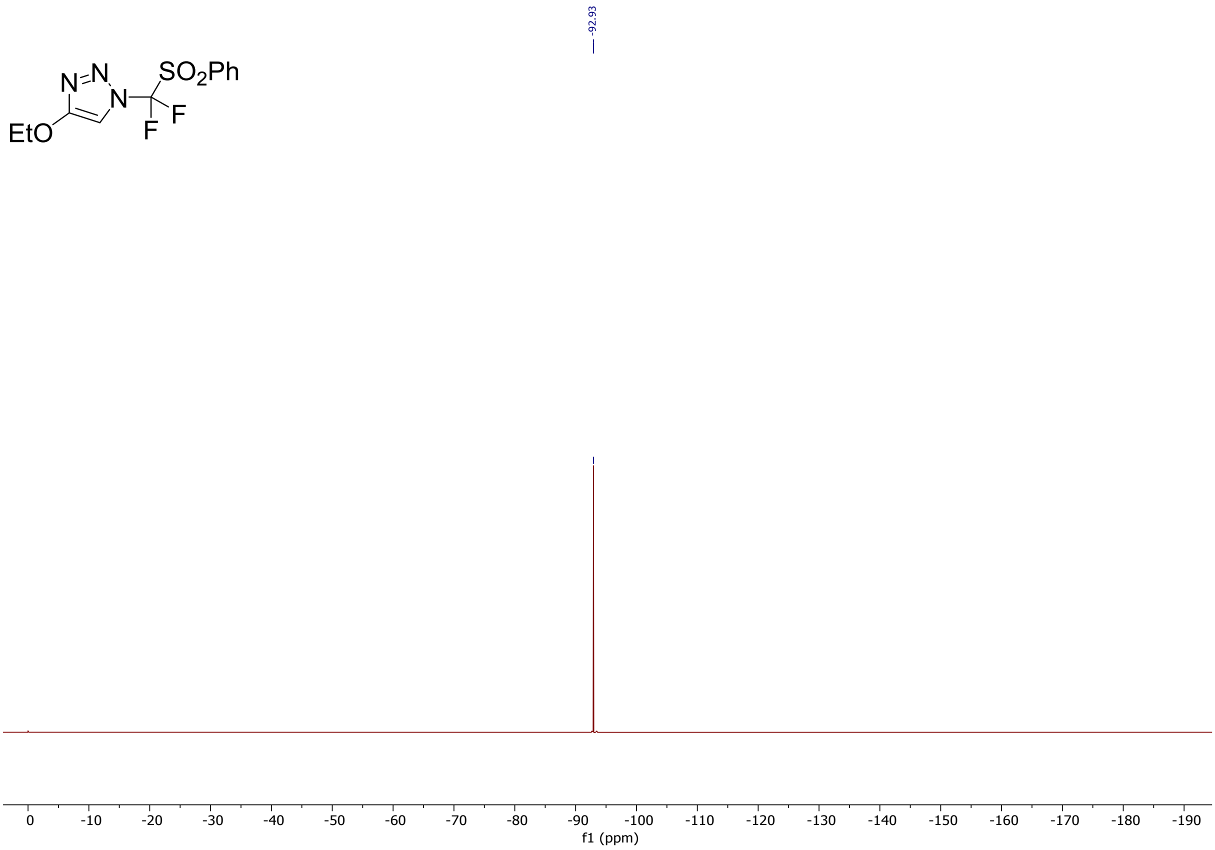

Figure S39. ^{1}H NMR spectrum of **2l** ($CDCl_3$, 400 MHz)

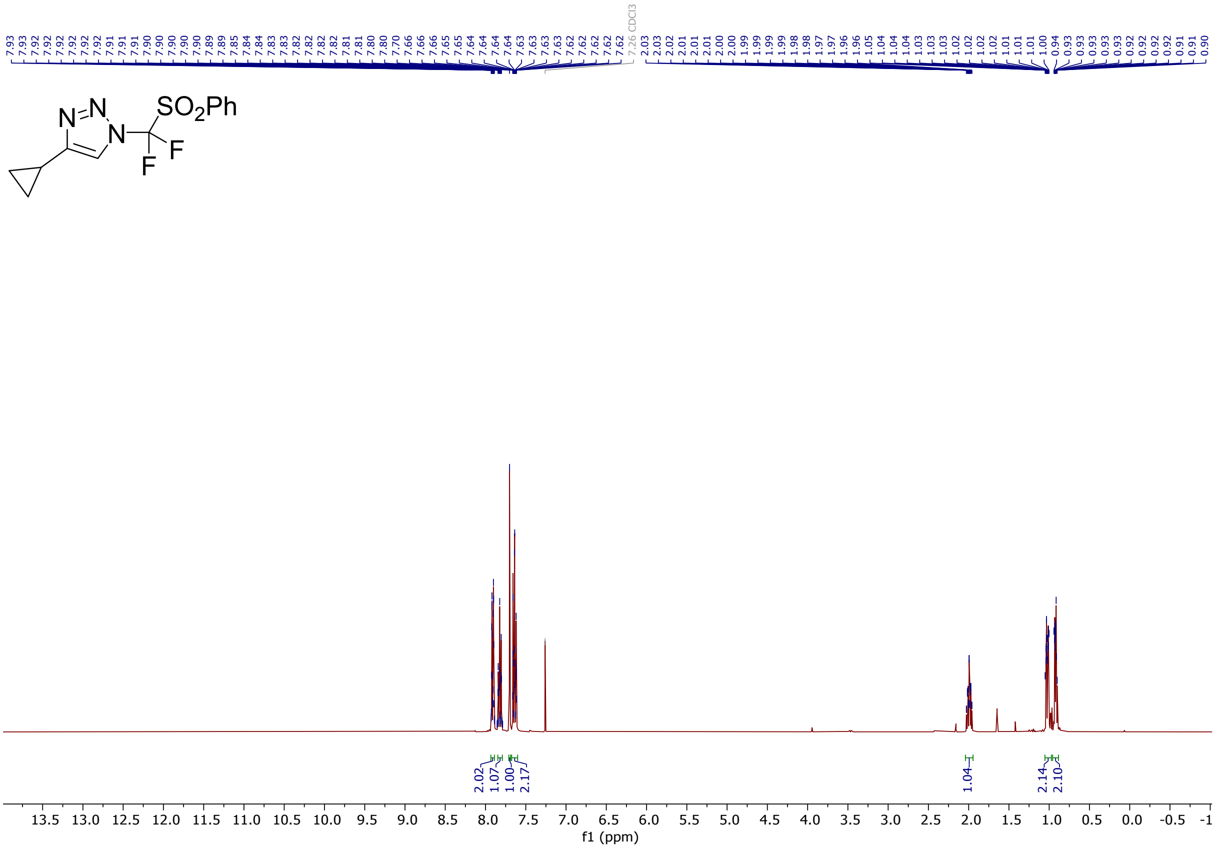

Figure S40. ^{13}C{^{1}H} NMR spectrum of **2l** ($CDCl_3$, 101 MHz)

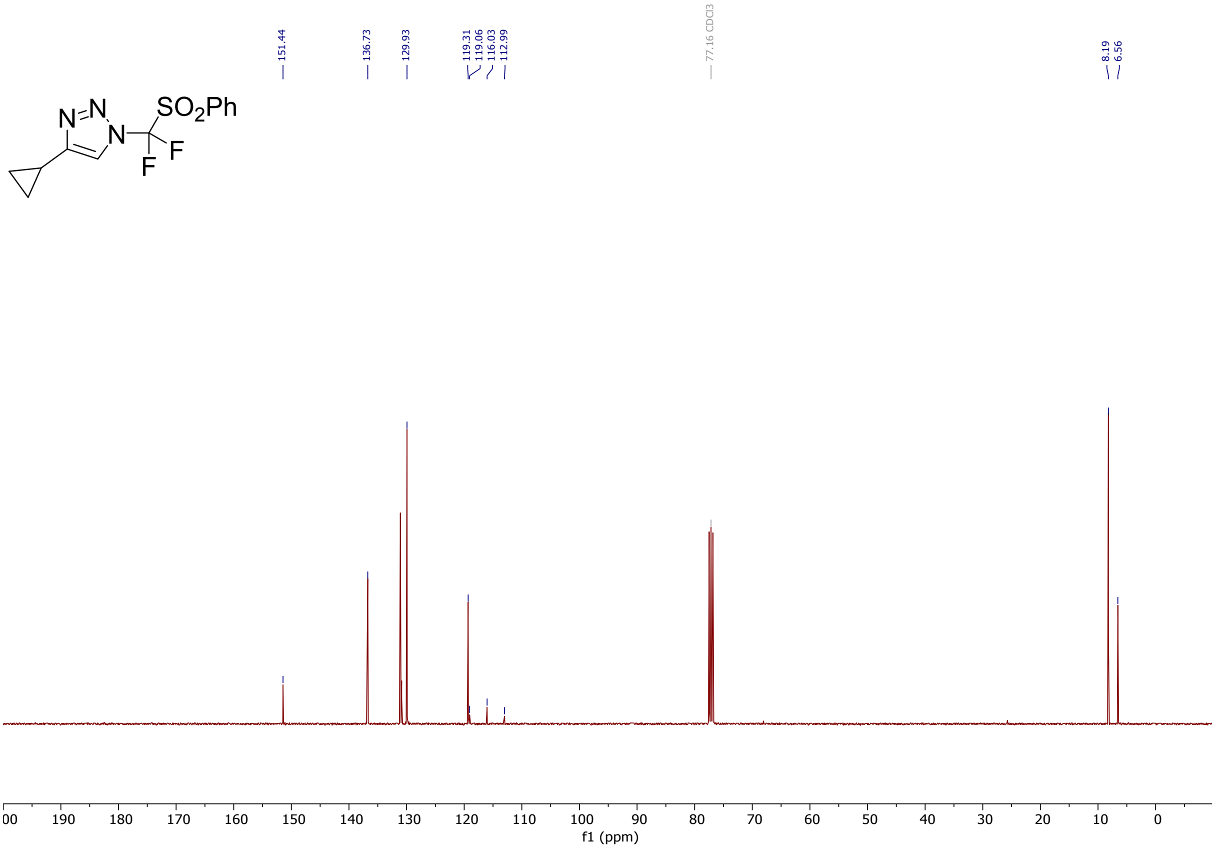

Figure S41. ^{19}F NMR spectrum of **2l** ($CDCl_3$, 377 MHz)

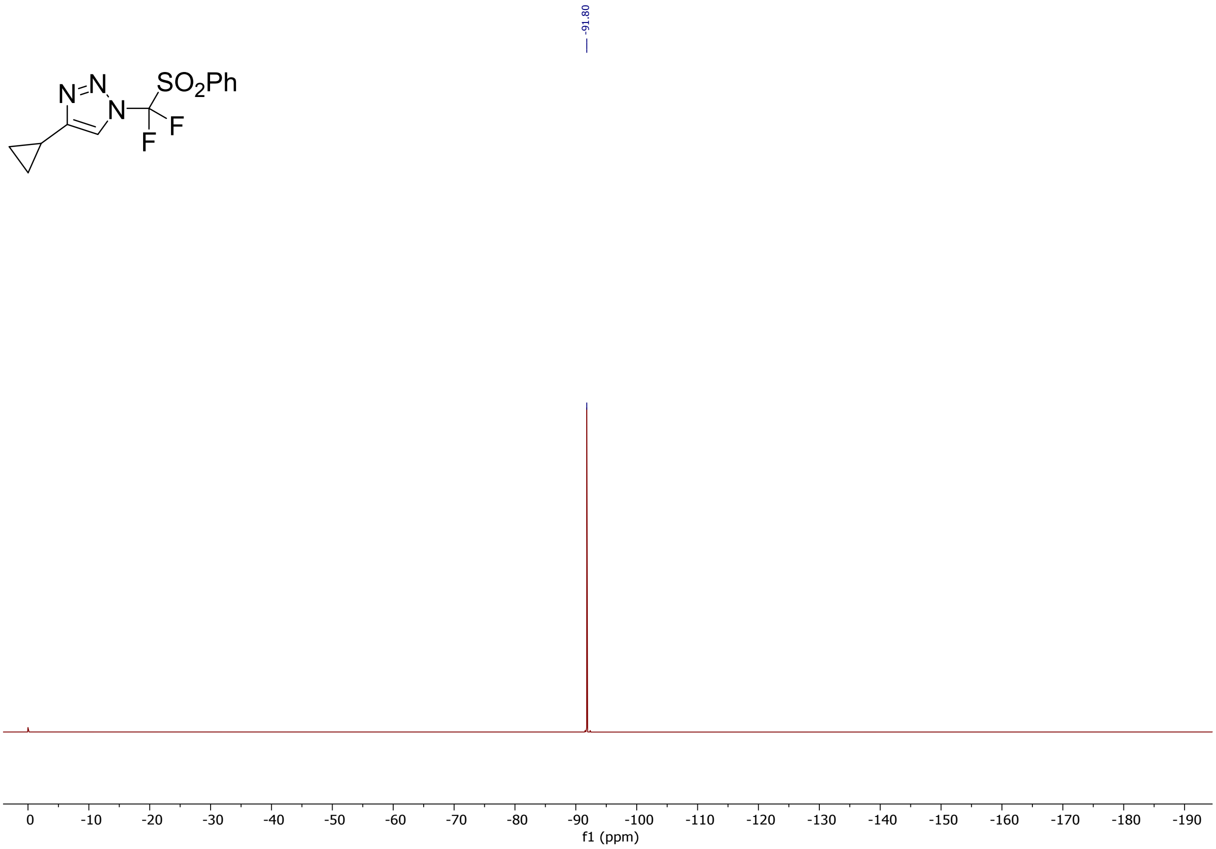

Figure S42. ^{1}H NMR spectrum of **2m** ($CDCl_3$, 400 MHz)

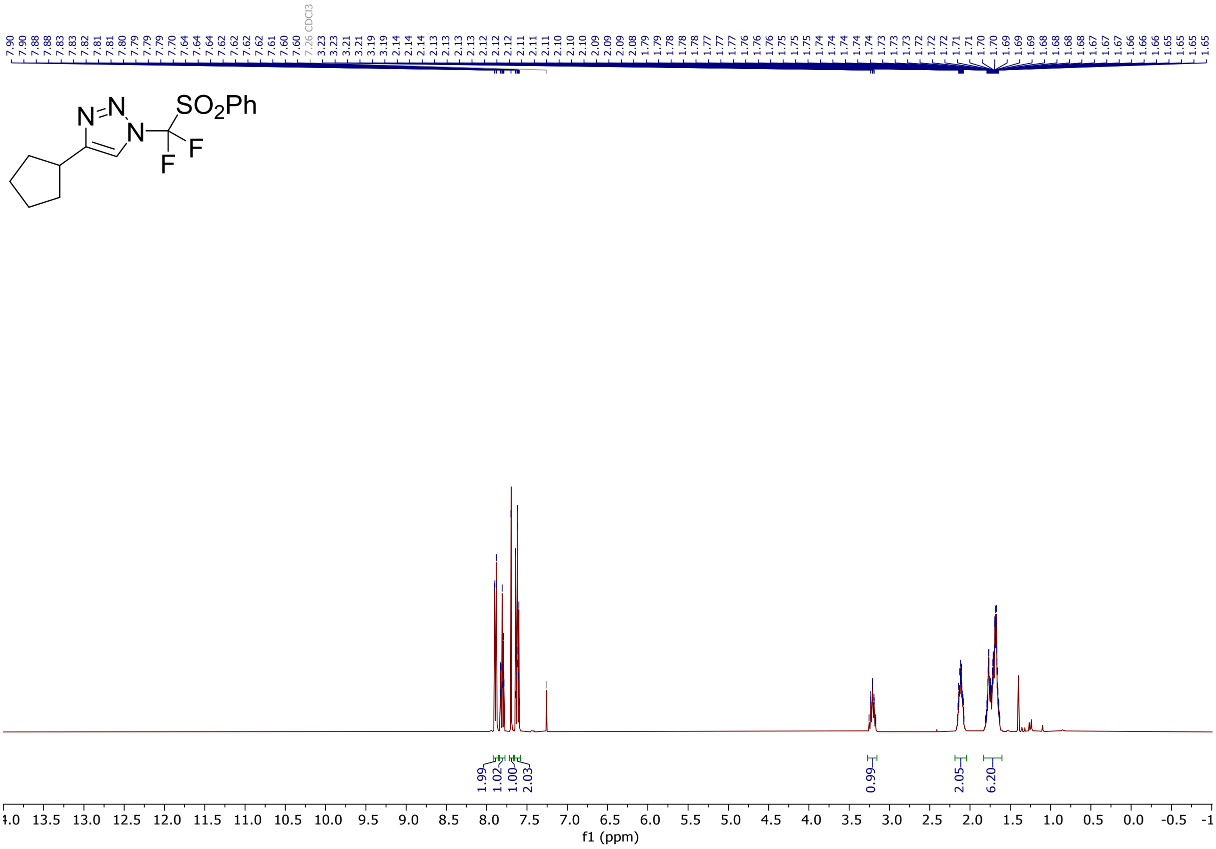

Figure S43. $^{13}C\{^{1}H\}$ NMR spectrum of **2m** ($CDCl_3$, 101 MHz)

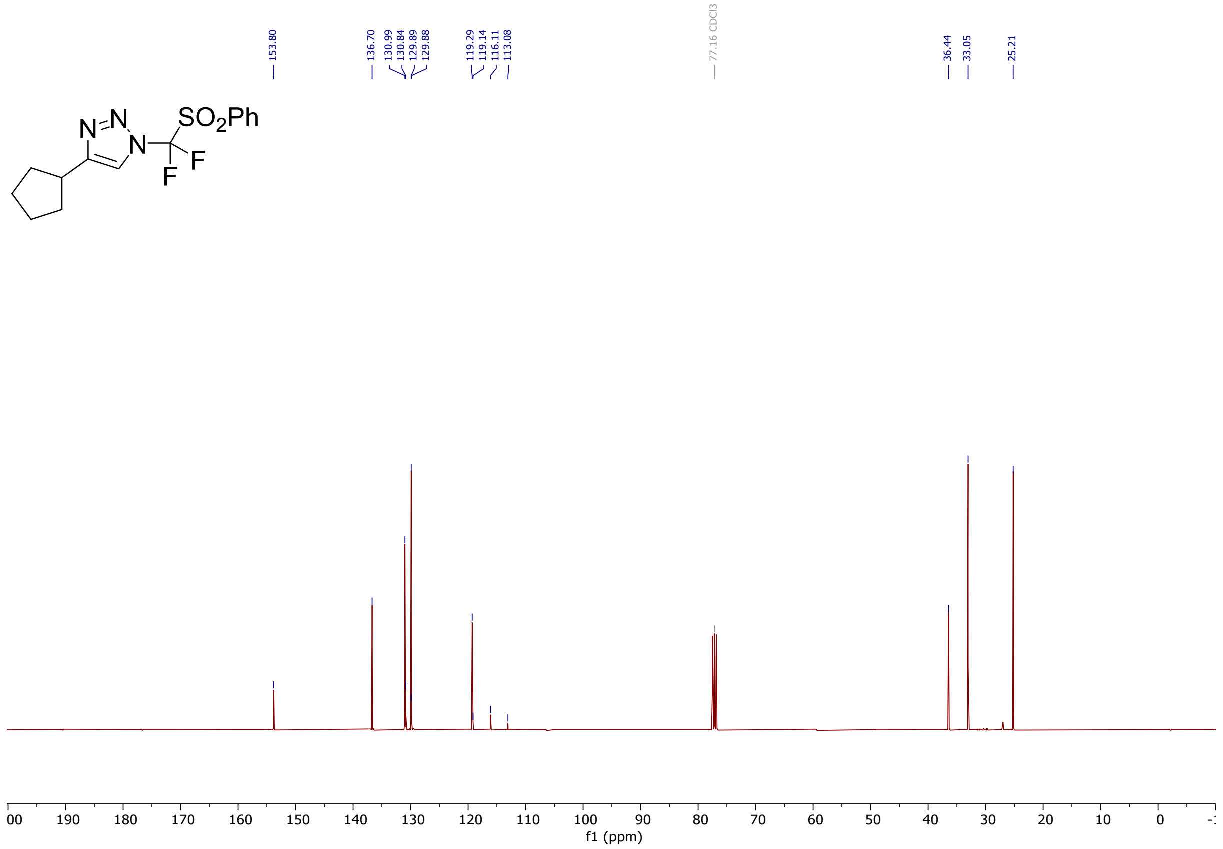

Figure S44. ^{19}F NMR spectrum of **2m** ($CDCl_3$, 377 MHz)

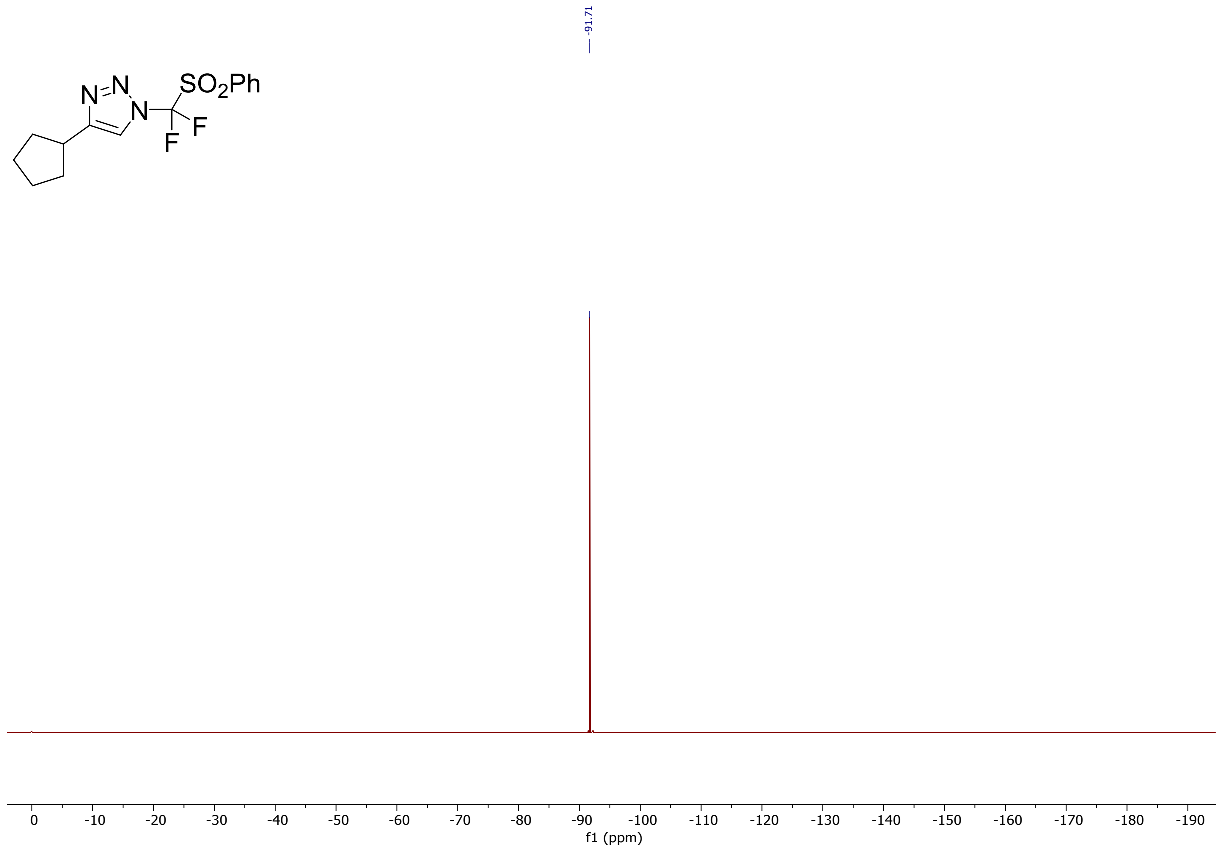

Figure S45. ^{1}H NMR spectrum of **2n** ($CDCl_3$, 400 MHz)

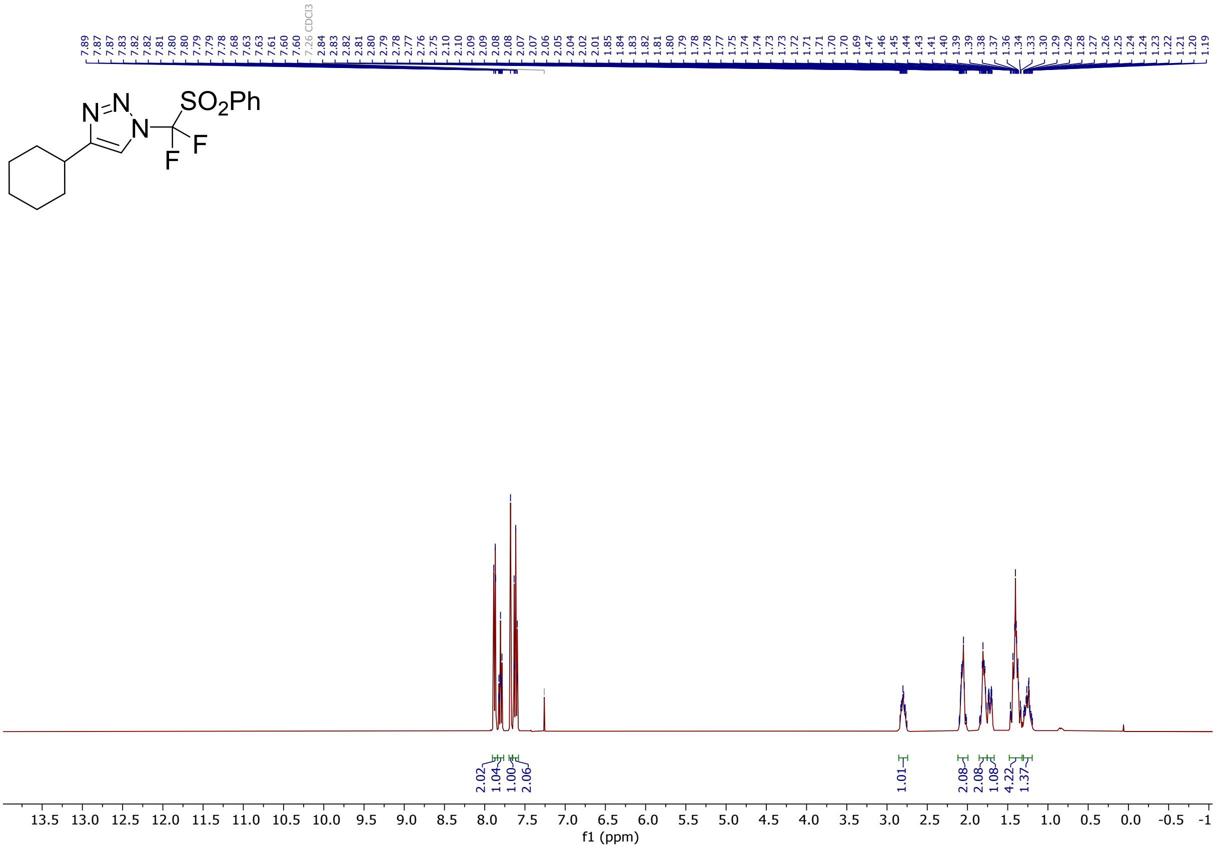

Figure S46. $^{13}C\{^{1}H\}$ NMR spectrum of **2n** ($CDCl_3$, 101 MHz)

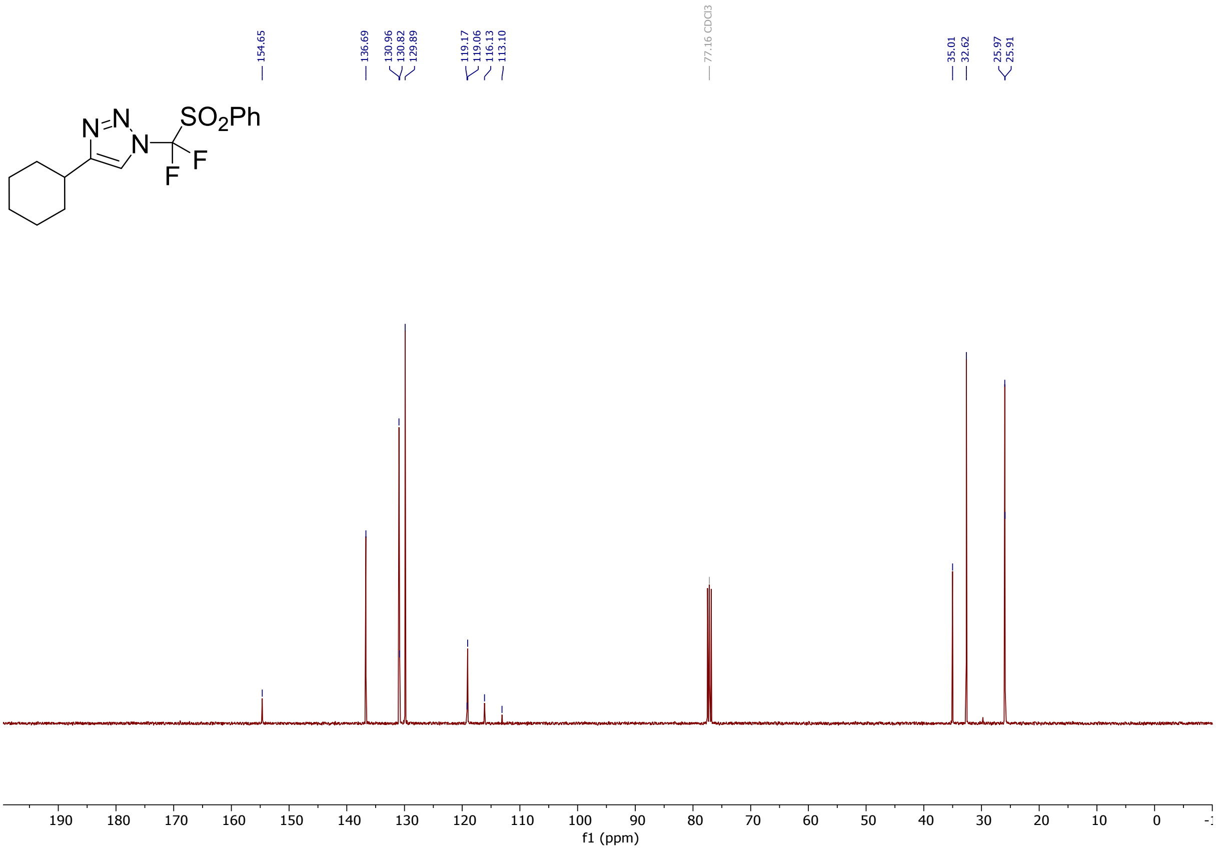

Figure S47. ^{19}F NMR spectrum of **2n** ($CDCl_3$, 377 MHz)

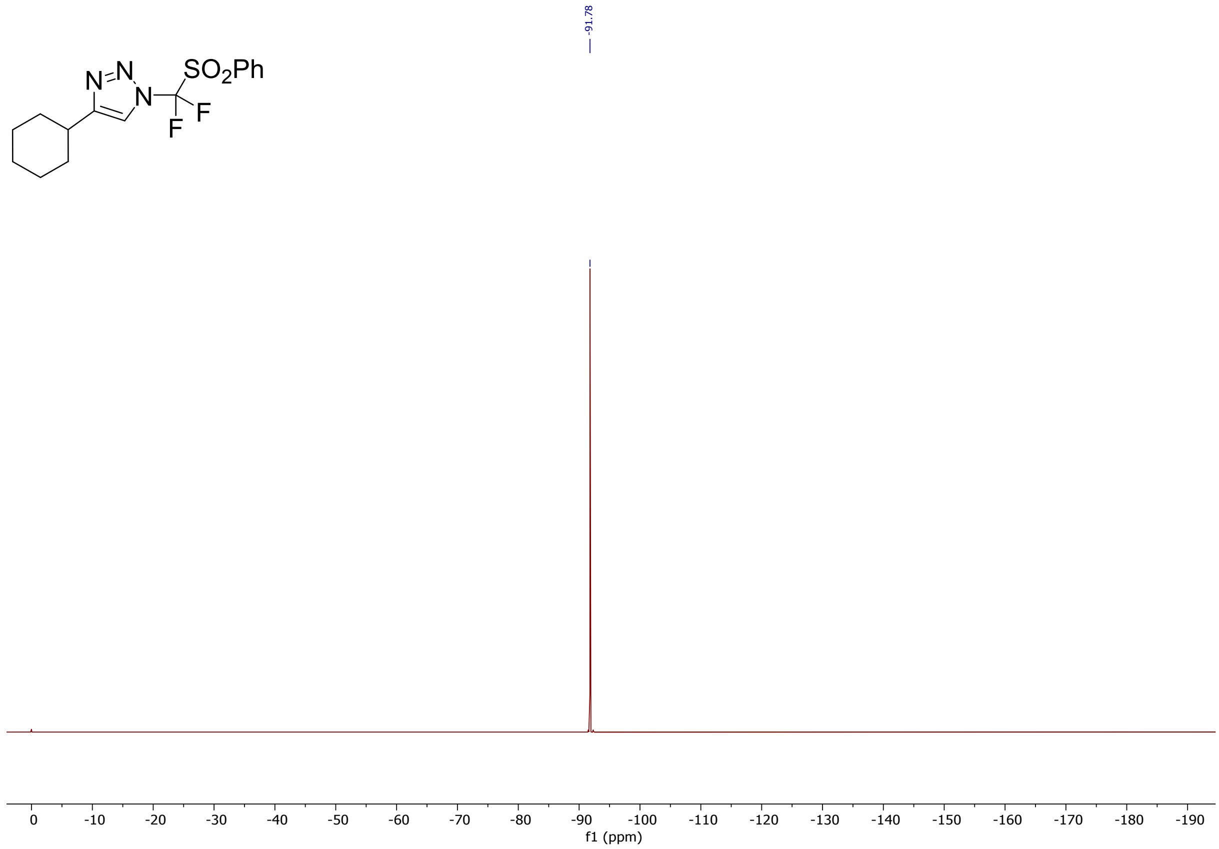

Figure S48. ^{1}H NMR spectrum of **2o** ($CDCl_3$, 400 MHz)

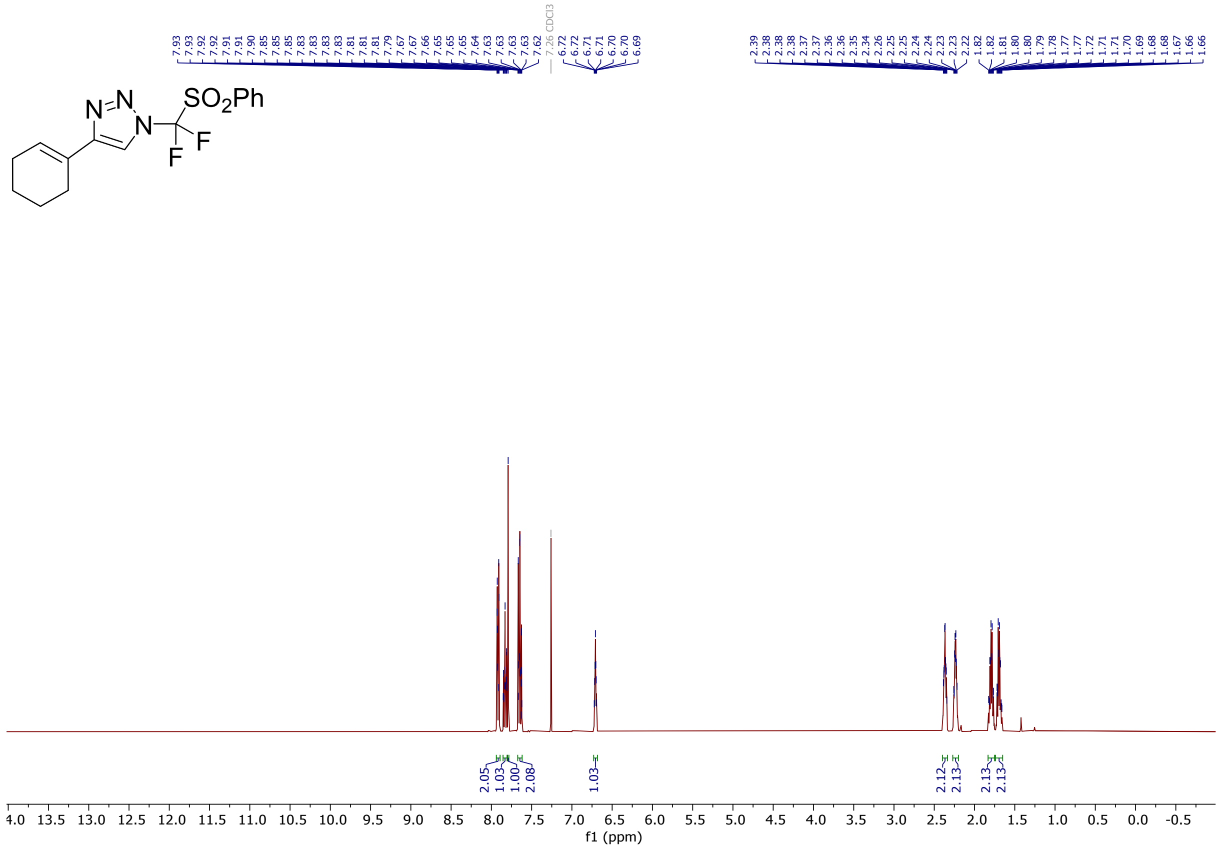

Figure S49. $^{13}C\{^1H\}$ NMR spectrum of **2o** ($CDCl_3$, 101 MHz)

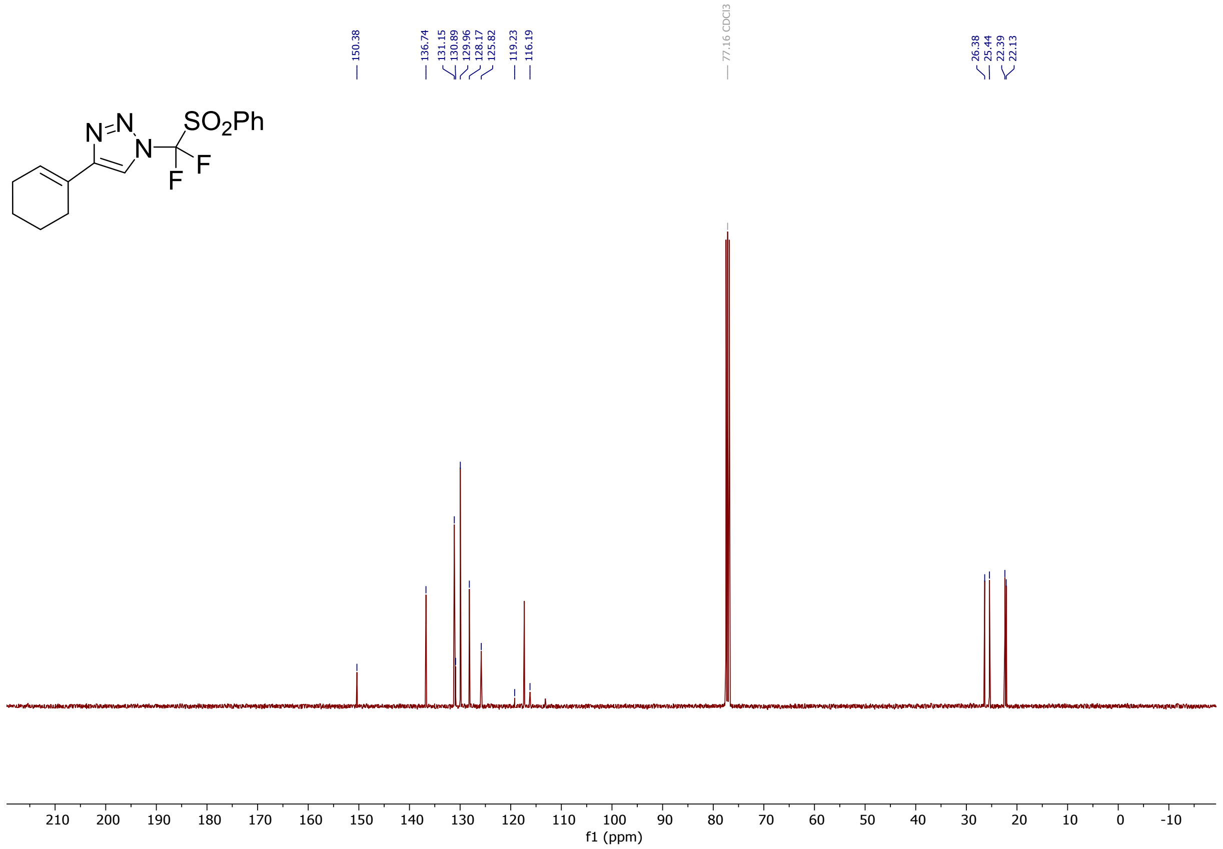

Figure S50. ^{19}F NMR spectrum of **2o** ($CDCl_3$, 377 MHz)

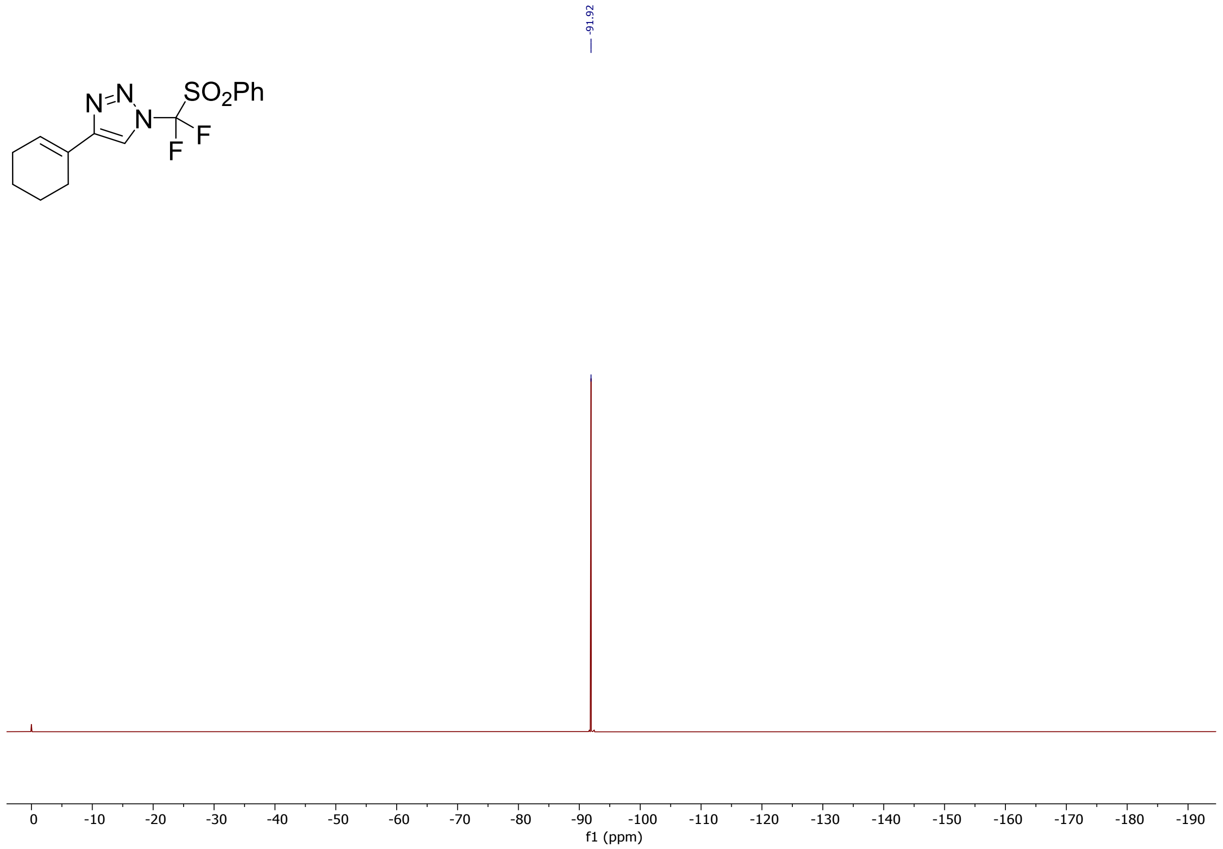

Figure S51. ^{1}H NMR spectrum of **2p** ($CDCl_3$, 400 MHz)

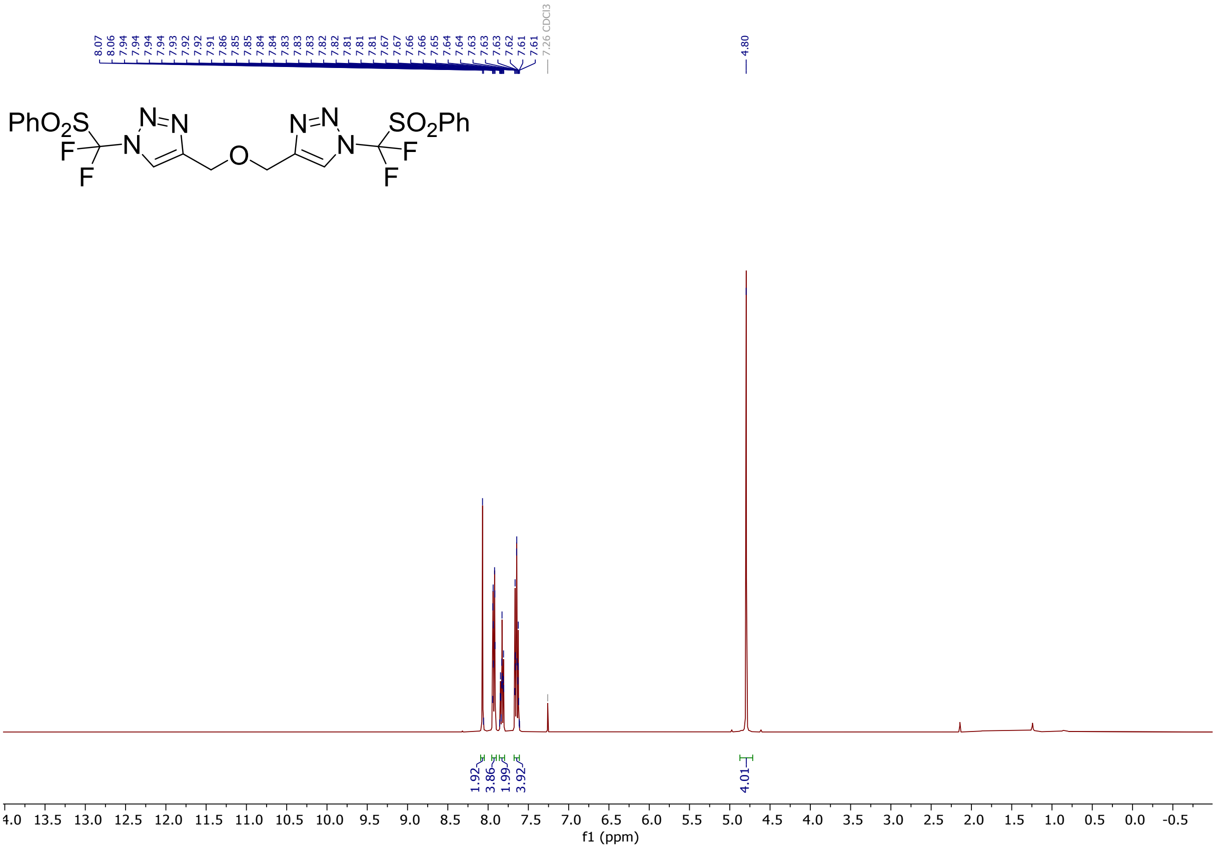

Figure S52. $^{13}C\{^{1}H\}$ NMR spectrum of **2p** ($CDCl_3$, 101 MHz)

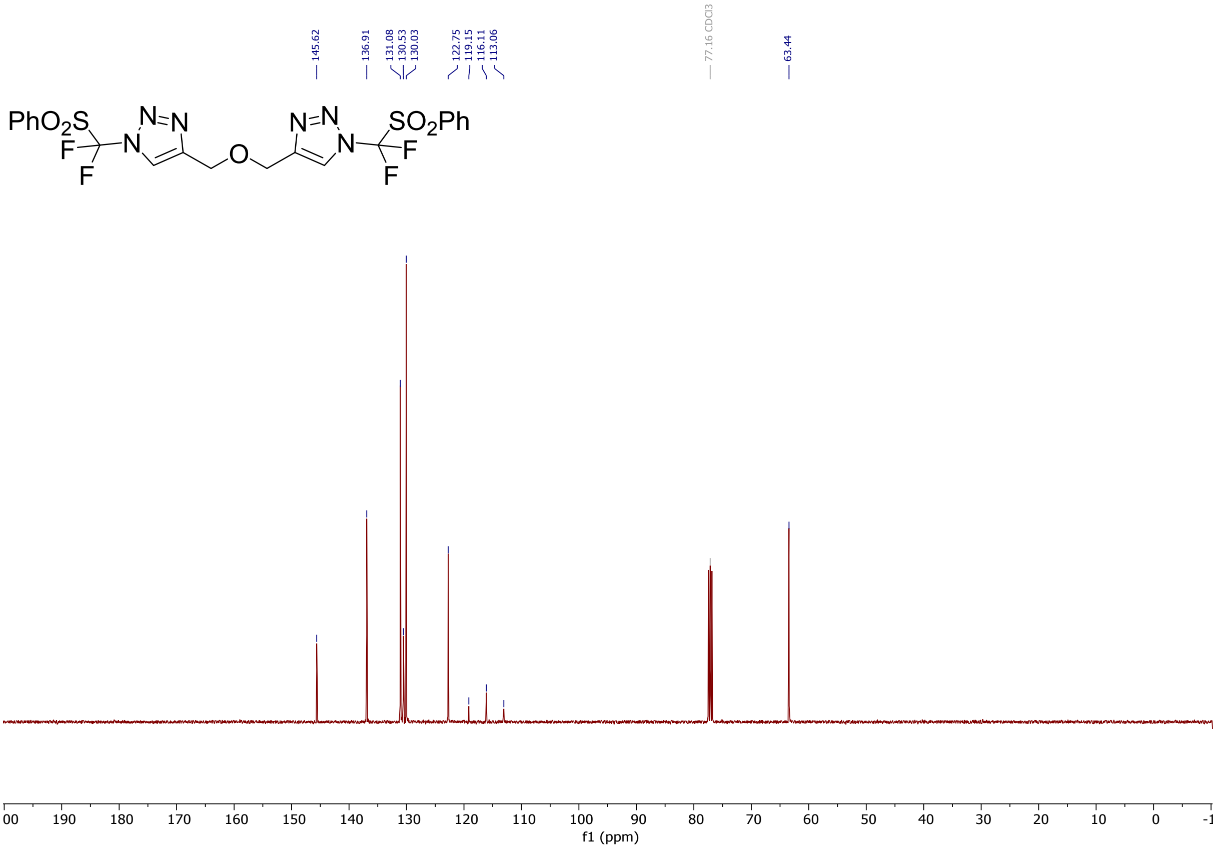

Figure S53. ^{19}F NMR spectrum of **2p** ($CDCl_3$, 377 MHz)

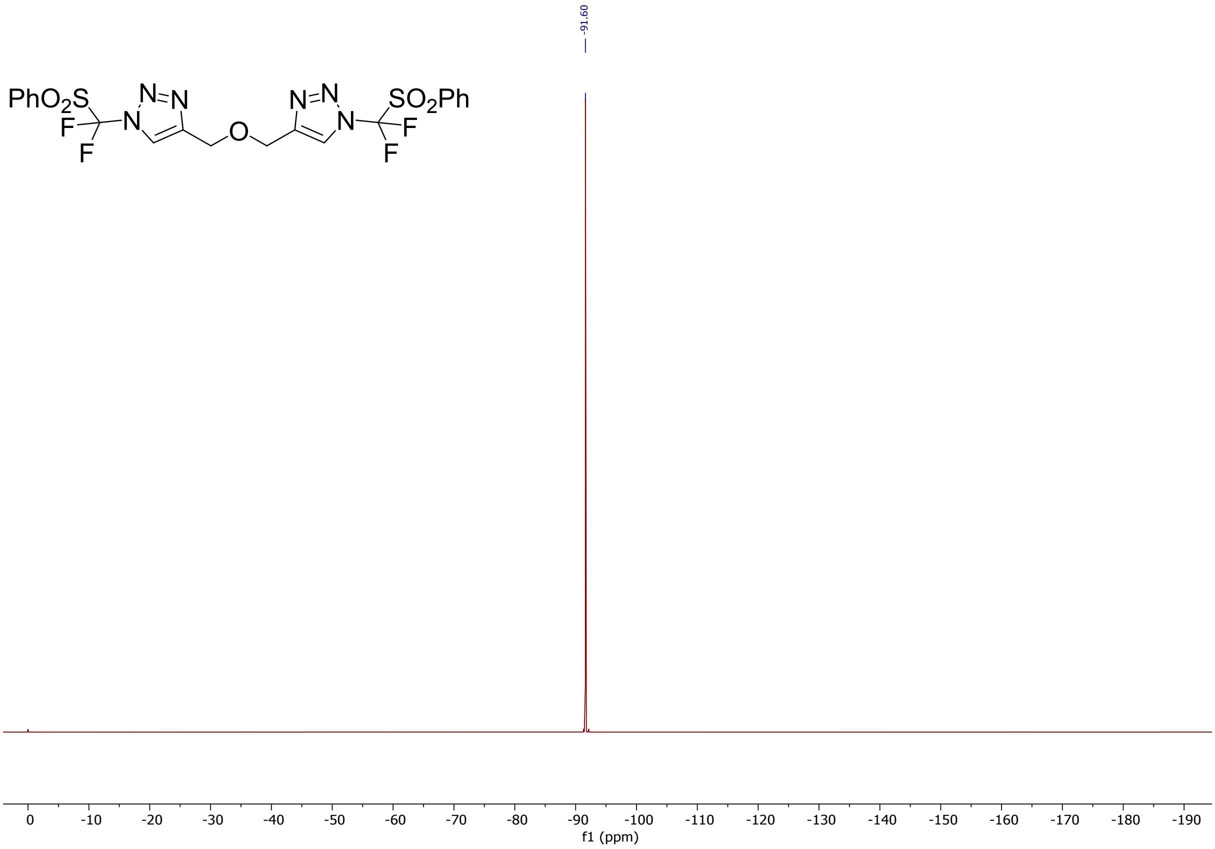

Figure S54. ^{1}H NMR spectrum of **3a** ($CDCl_3$, 400 MHz)

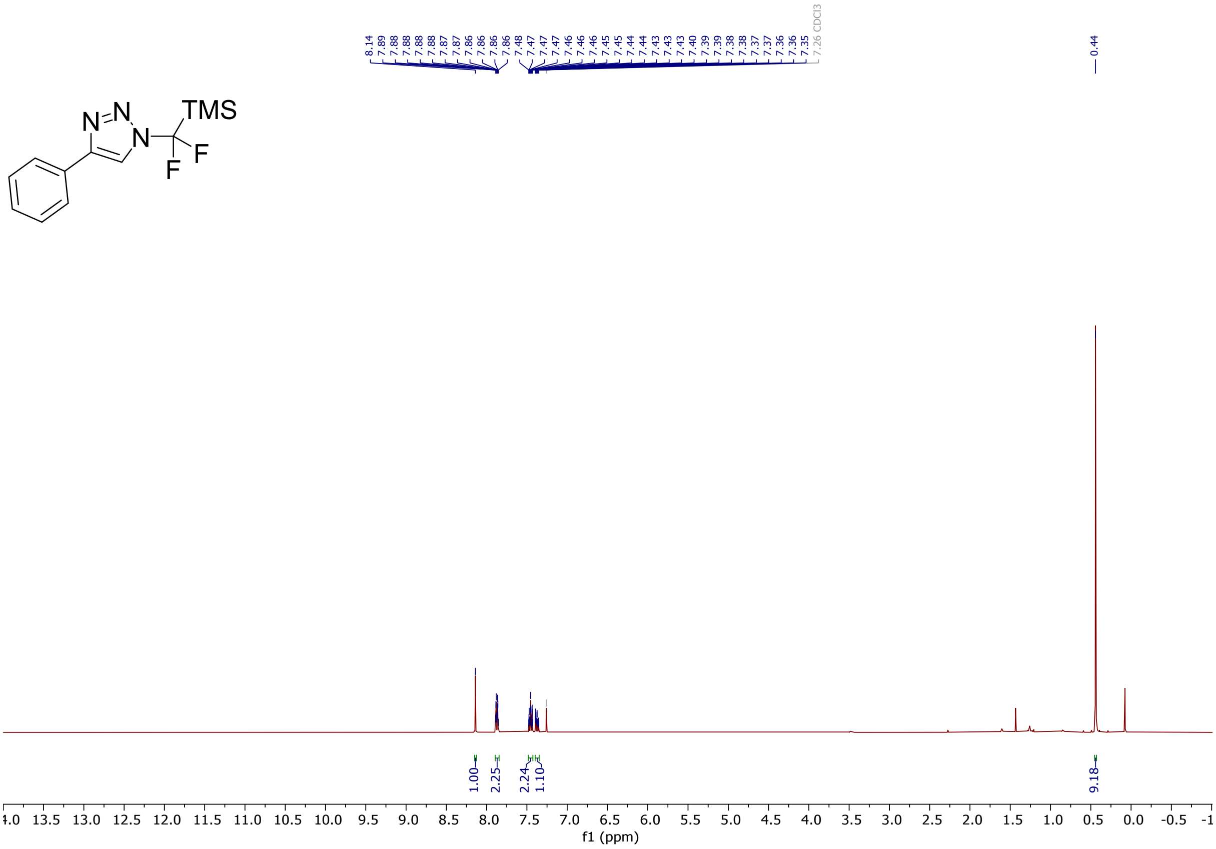

Figure S55. $^{13}C\{^{1}H\}$ NMR spectrum of **3a** ($CDCl_3$, 101 MHz)

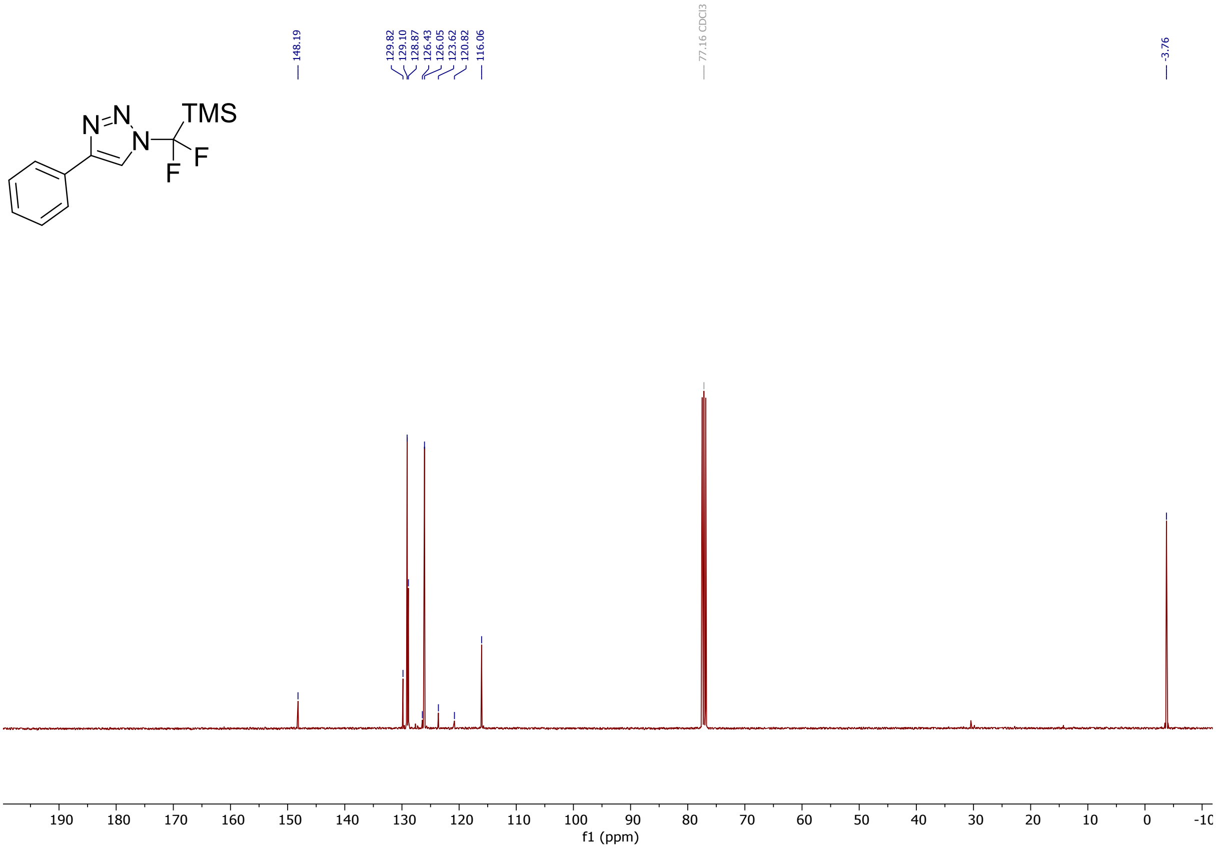

Figure S56. ^{19}F NMR spectrum of **3a** ($CDCl_3$, 377 MHz)

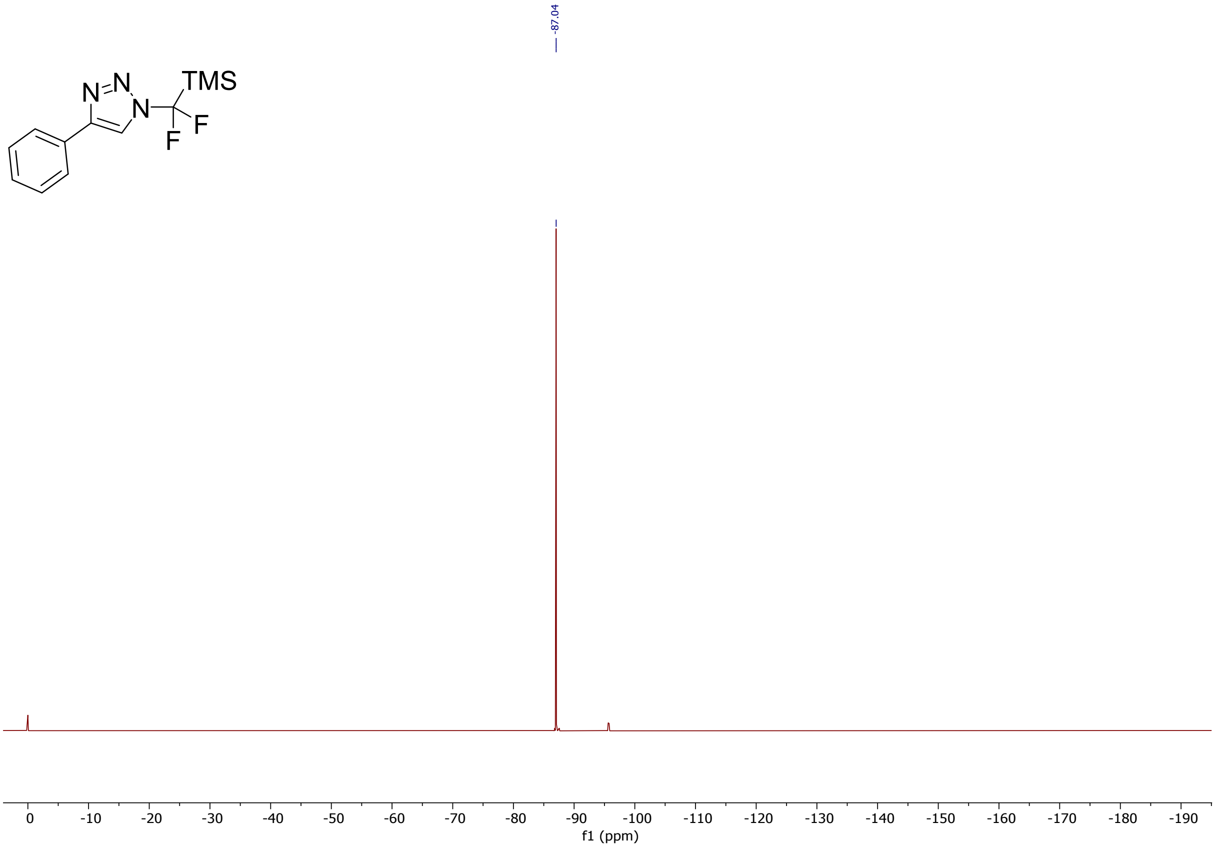

Figure S57. ^{1}H NMR spectrum of **3b** ($CDCl_3$, 400 MHz)

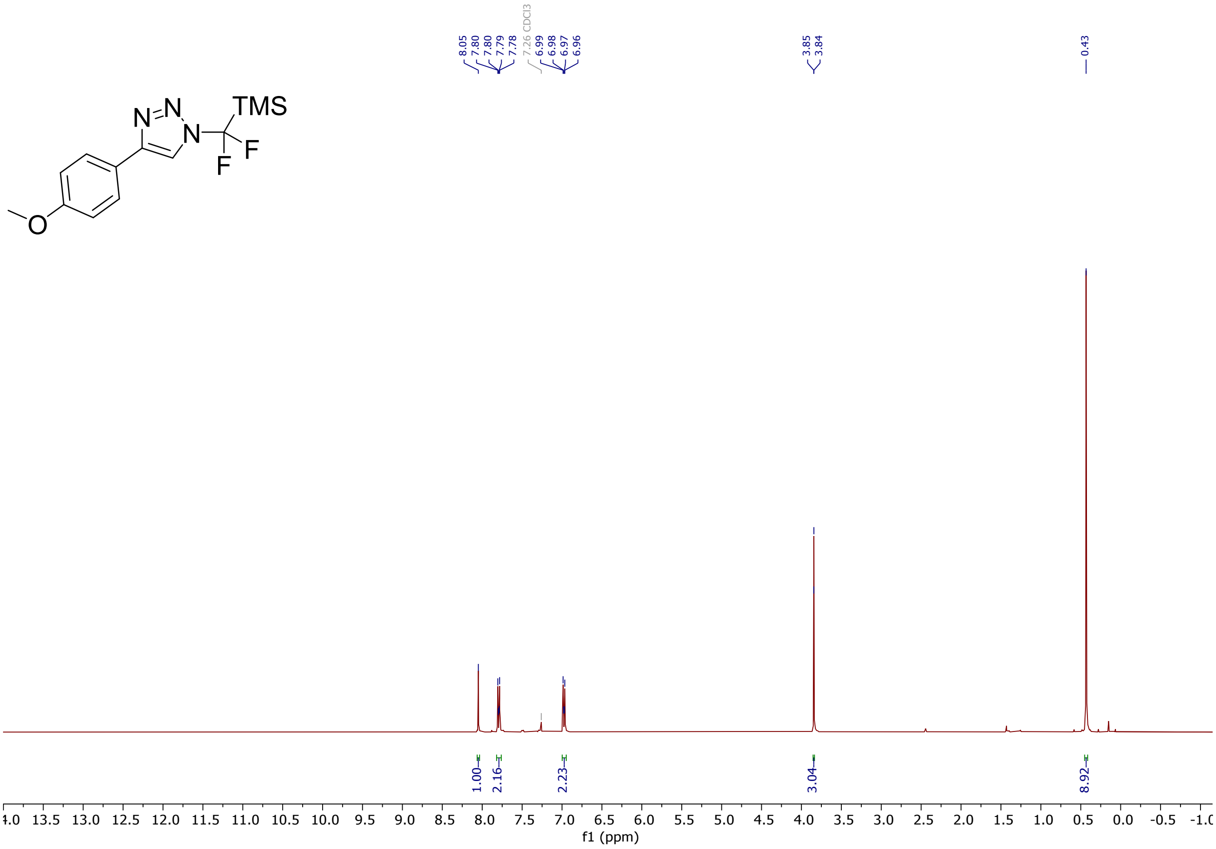

Figure S58. ^{13}C{^{1}H} NMR spectrum of **3b** ($CDCl_3$, 101 MHz)

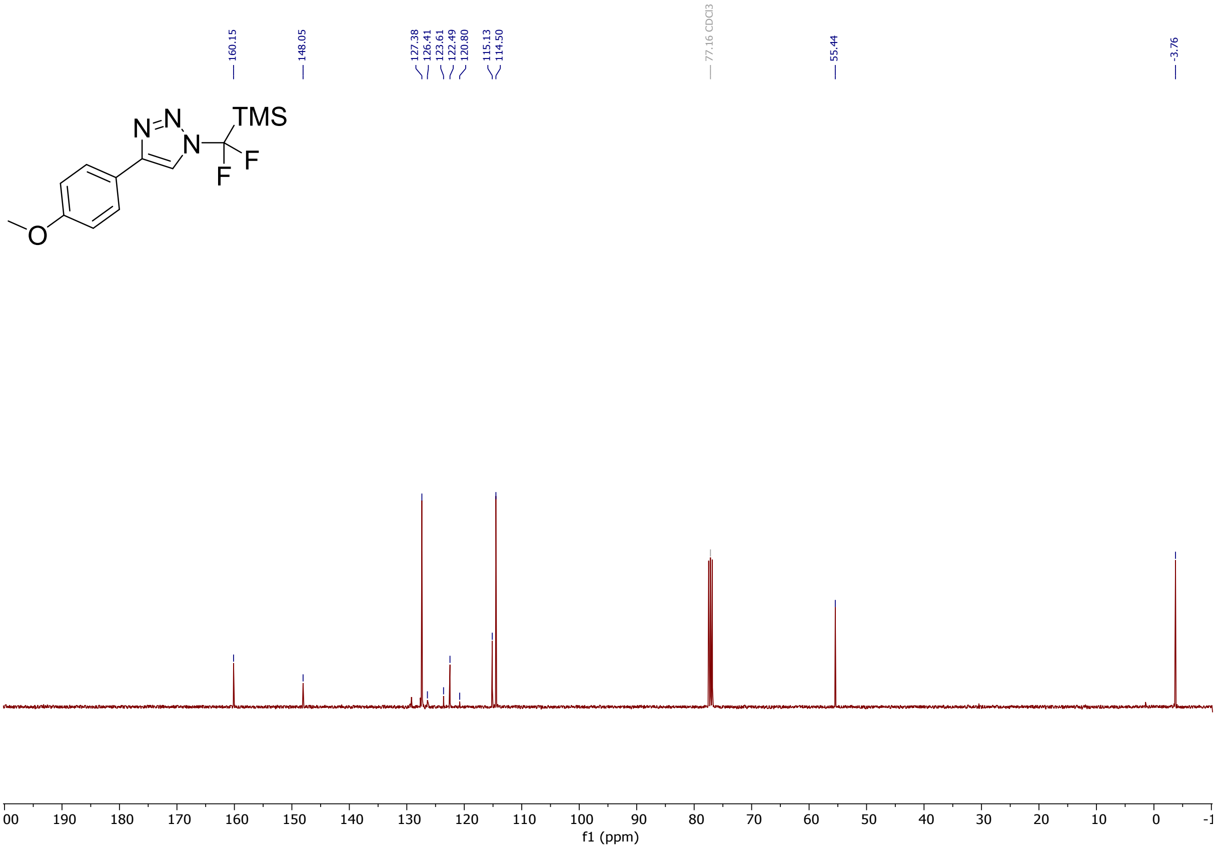

Figure S59. ^{19}F NMR spectrum of **3b** ($CDCl_3$, 377 MHz)

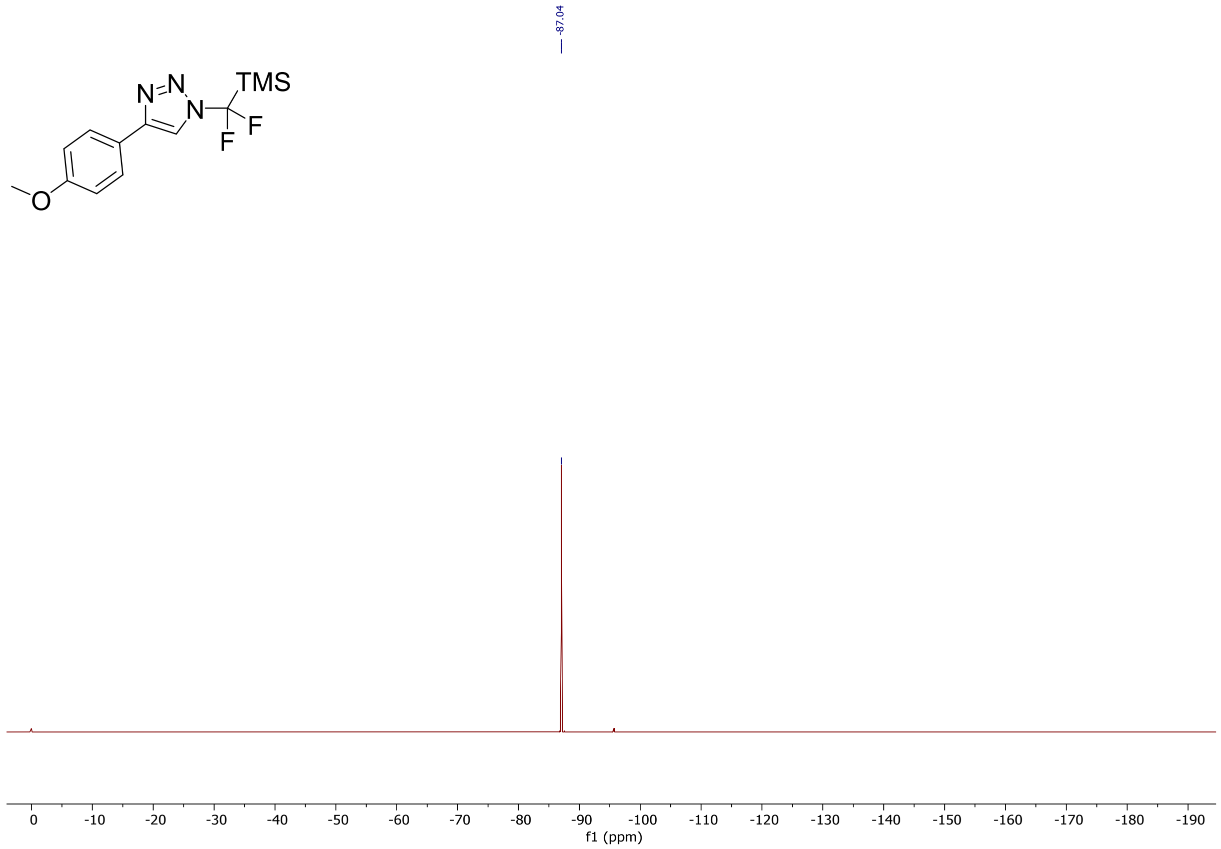

Figure S60. ^{1}H NMR spectrum of **3c** ($CDCl_3$, 400 MHz)

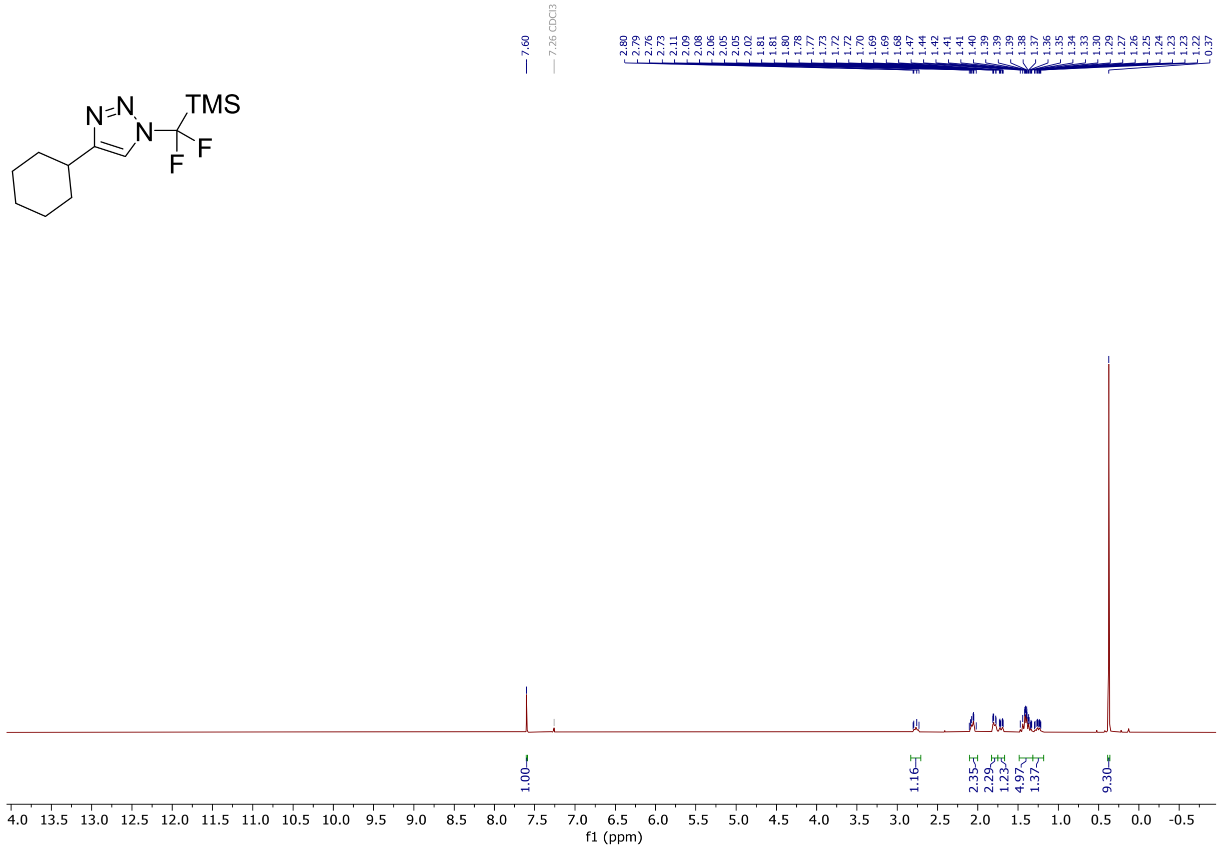

Figure S61. $^{13}C\{^{1}H\}$ NMR spectrum of **3c** ($CDCl_3$, 101 MHz)

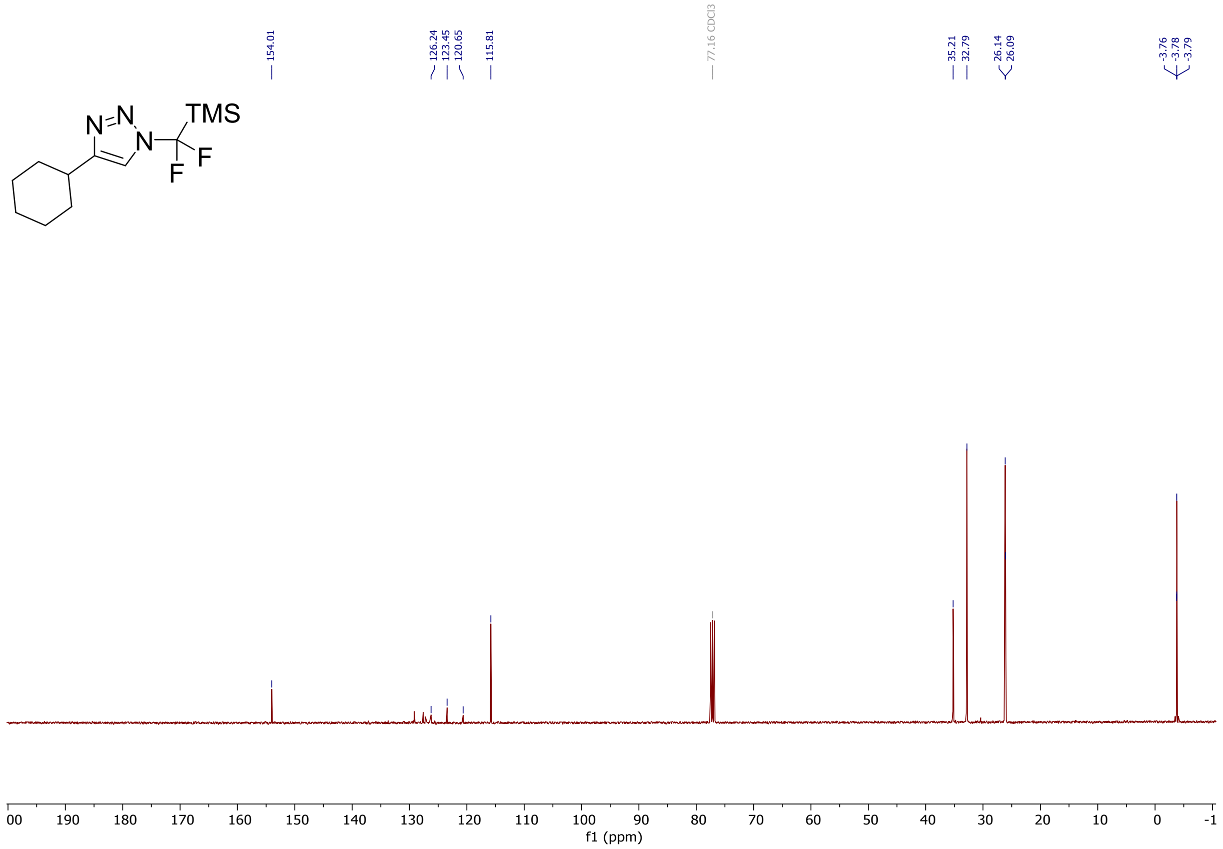

Figure S62. ^{19}F NMR spectrum of **3c** ($CDCl_3$, 377 MHz)

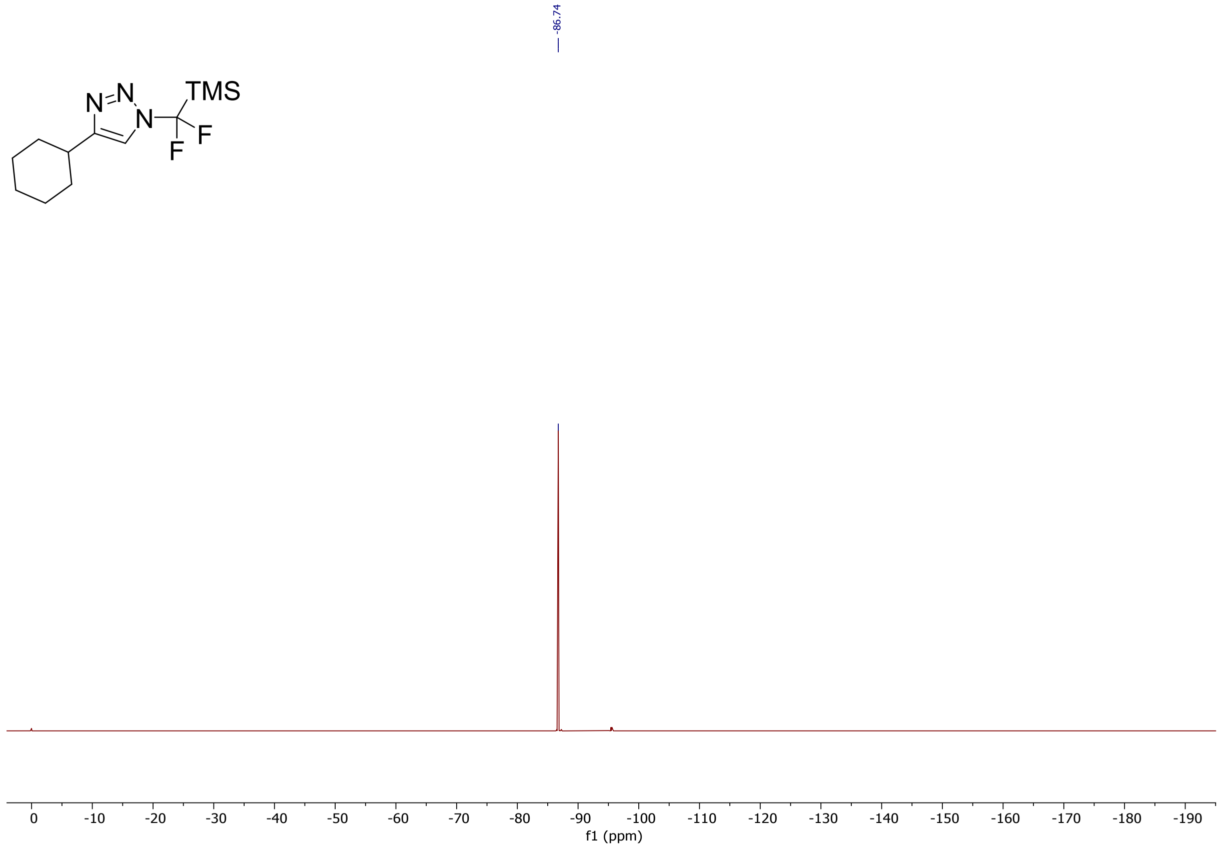

Figure S63. ^{1}H NMR spectrum of **4** ($CDCl_3$, 400 MHz)

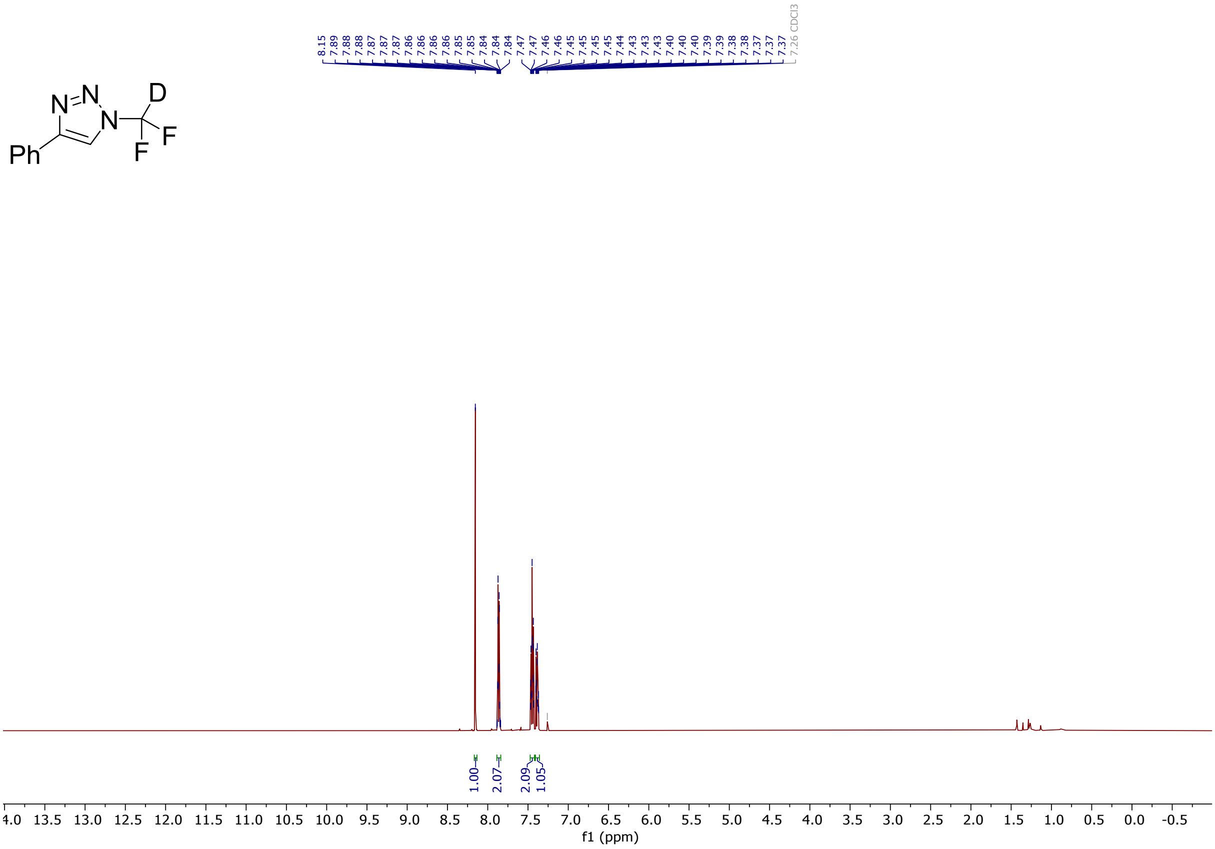

Figure S64. $^{13}C\{^{1}H\}$ NMR spectrum of **4** ($CDCl_3$, 101 MHz)

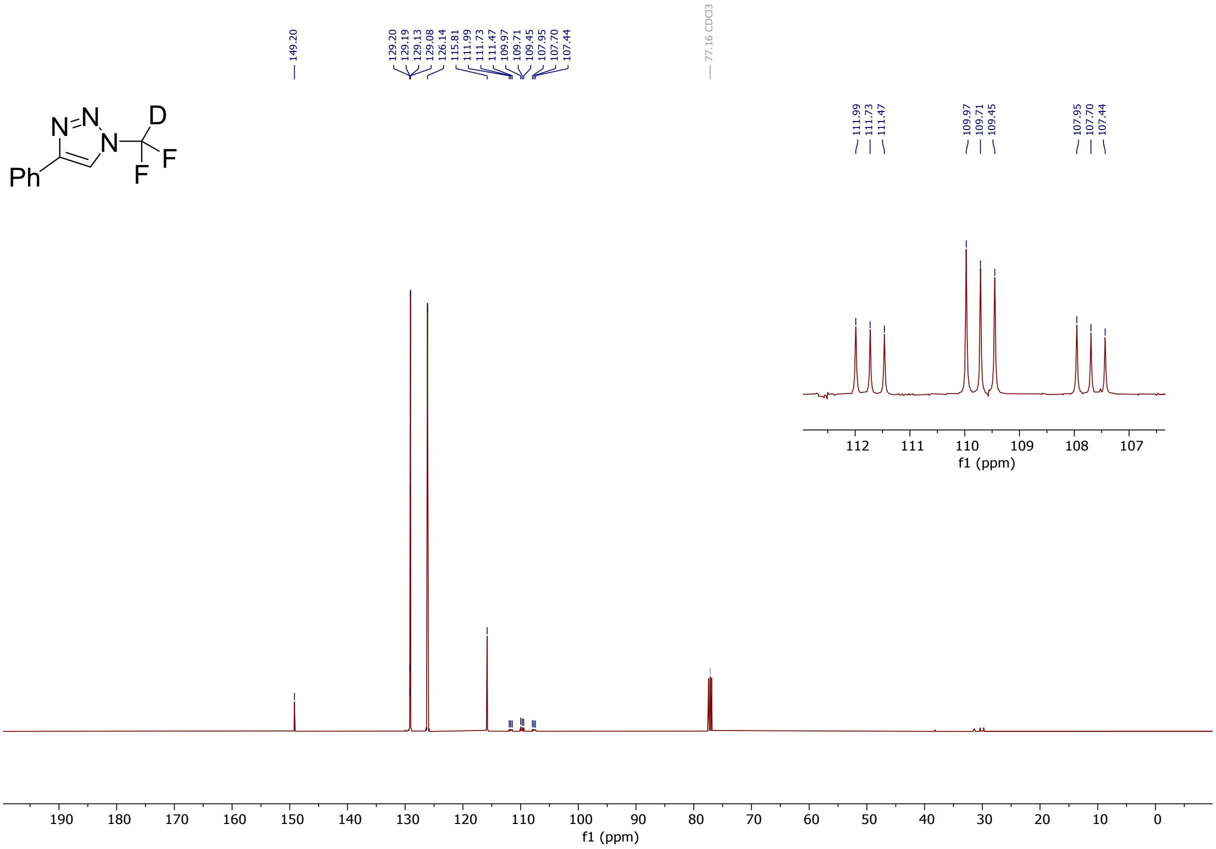

Figure S65. ^{19}F NMR spectrum of **4** ($CDCl_3$, 377 MHz)

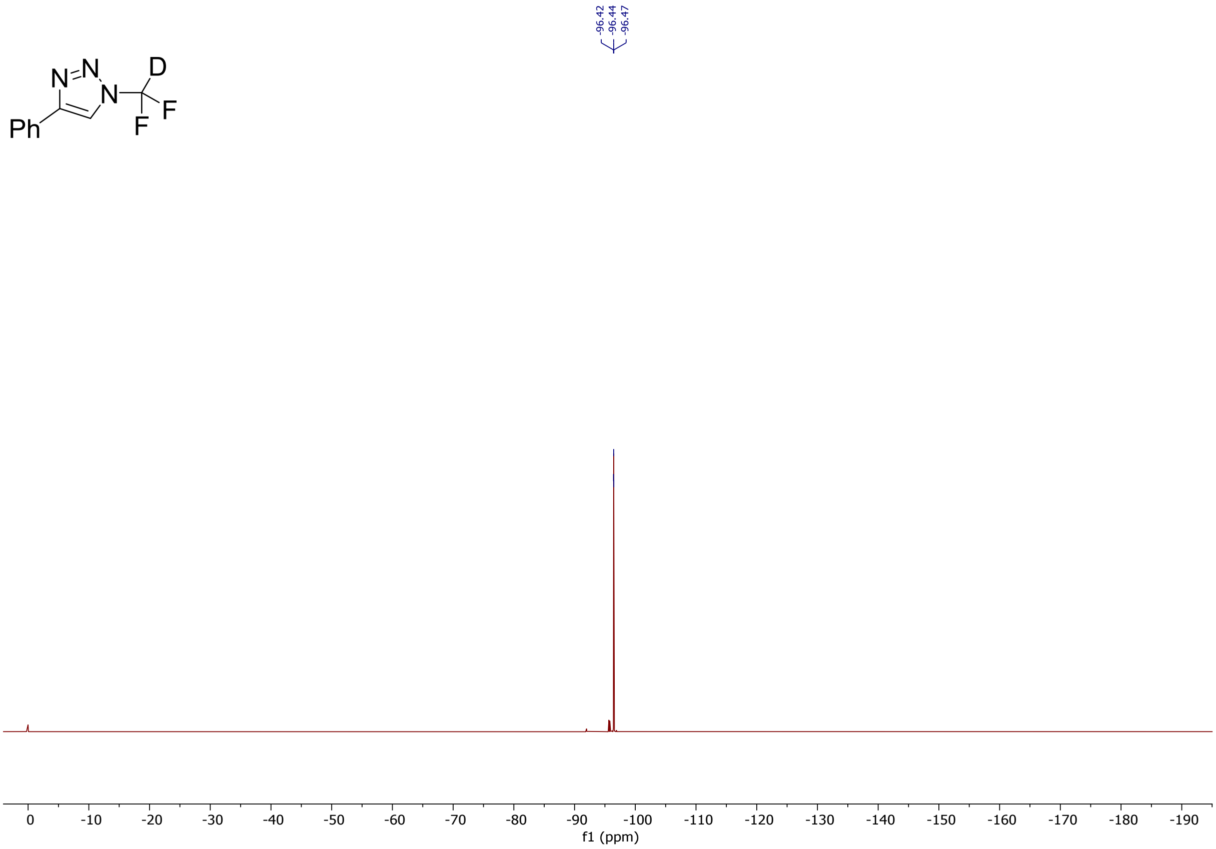

Figure S66. ^{1}H NMR spectrum of **5** ($CDCl_3$, 400 MHz)

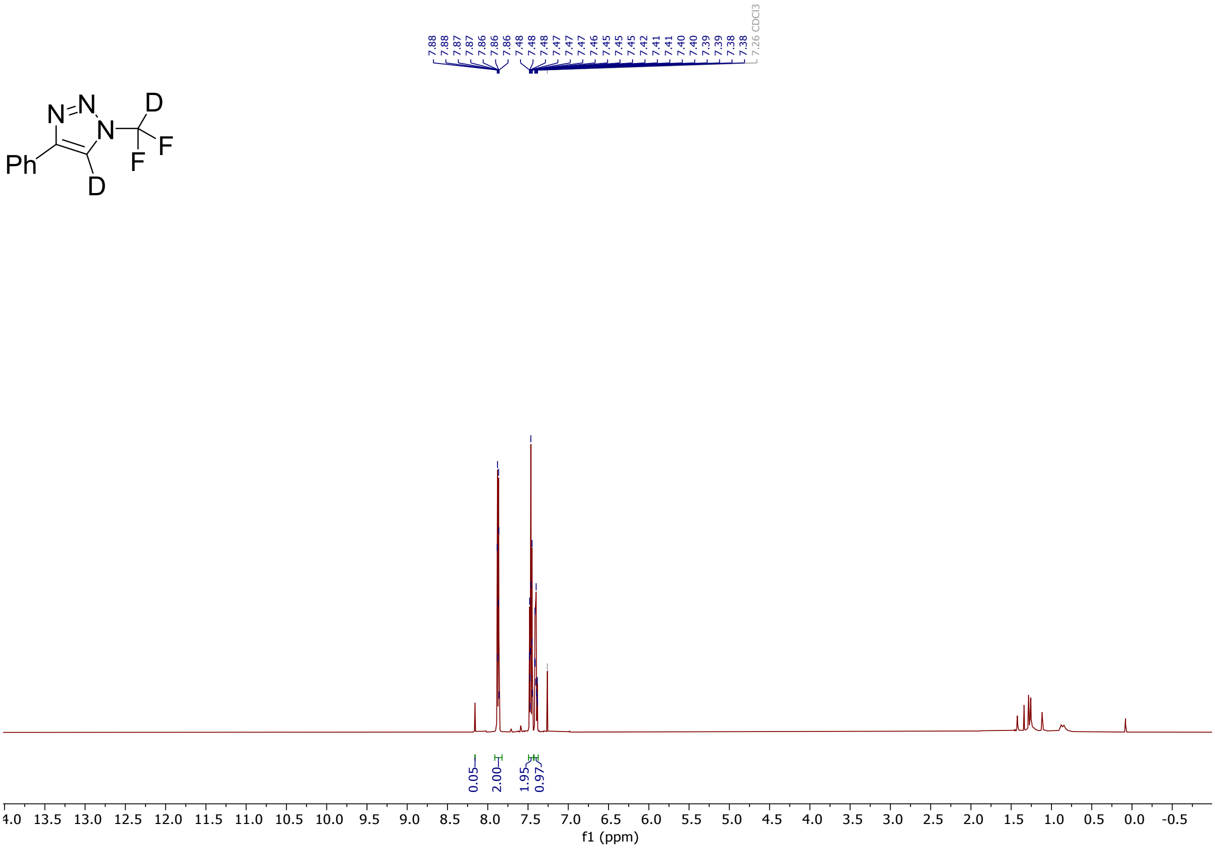

Figure S67. $^{13}C\{^{1}H\}$ NMR spectrum of **5** ($CDCl_3$, 101 MHz)

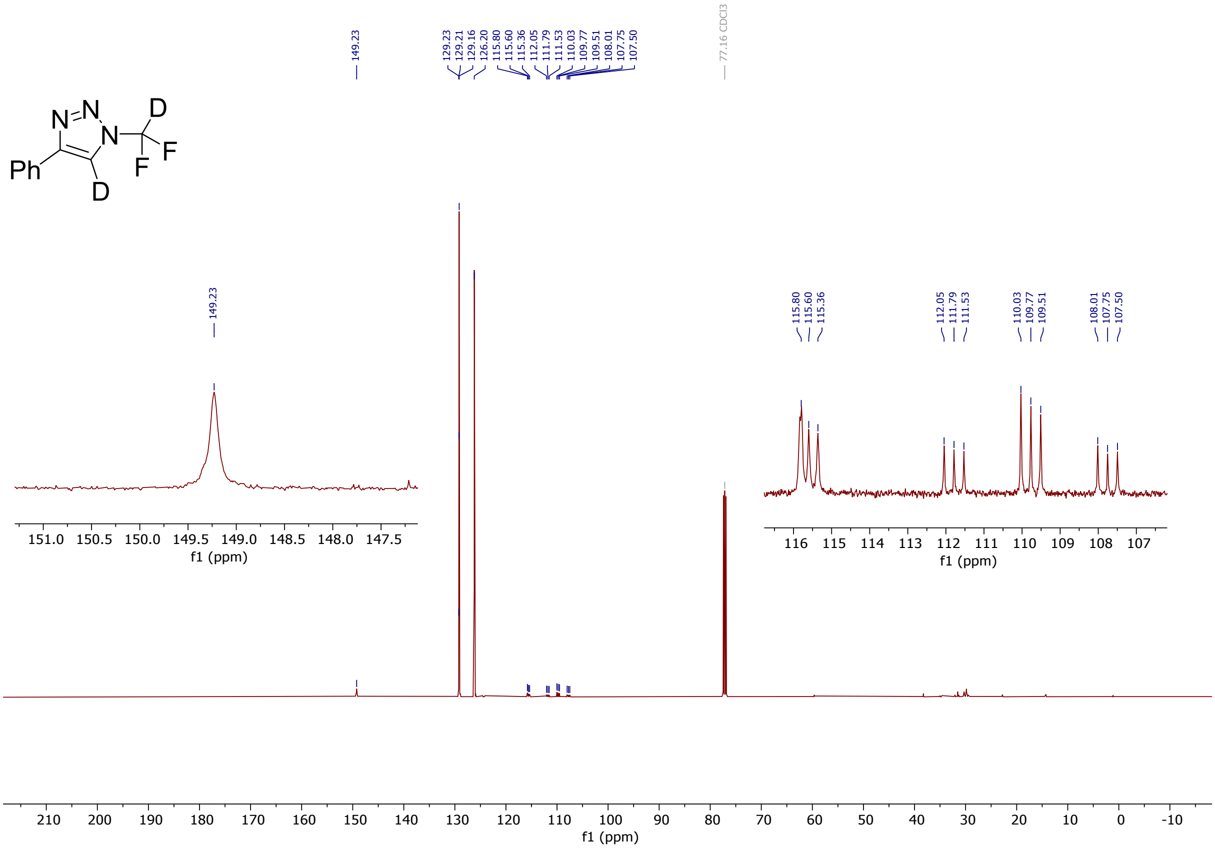

Figure S68. ^{19}F NMR spectrum of **5** ($CDCl_3$, 377 MHz)

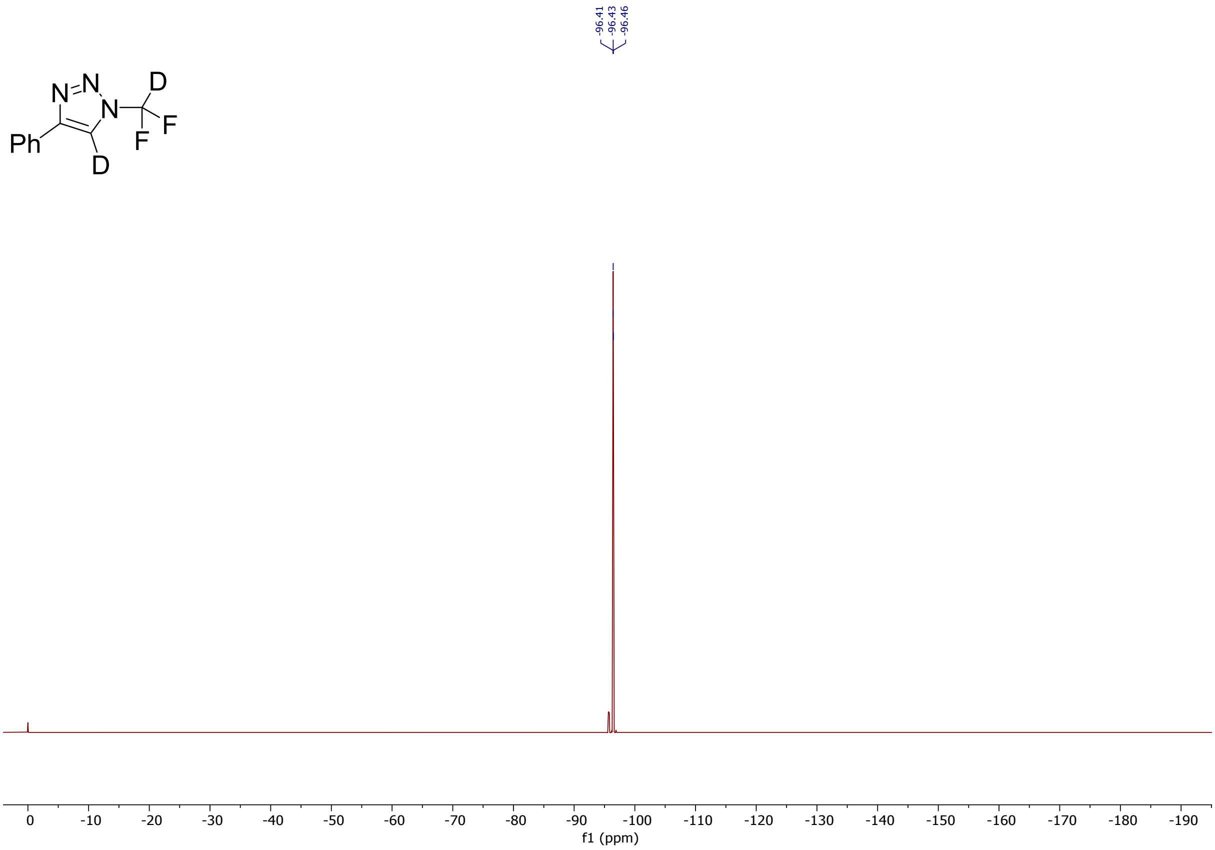

Figure S69. ^{1}H NMR spectrum of **6** ($CDCl_3$, 400 MHz)

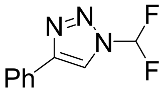

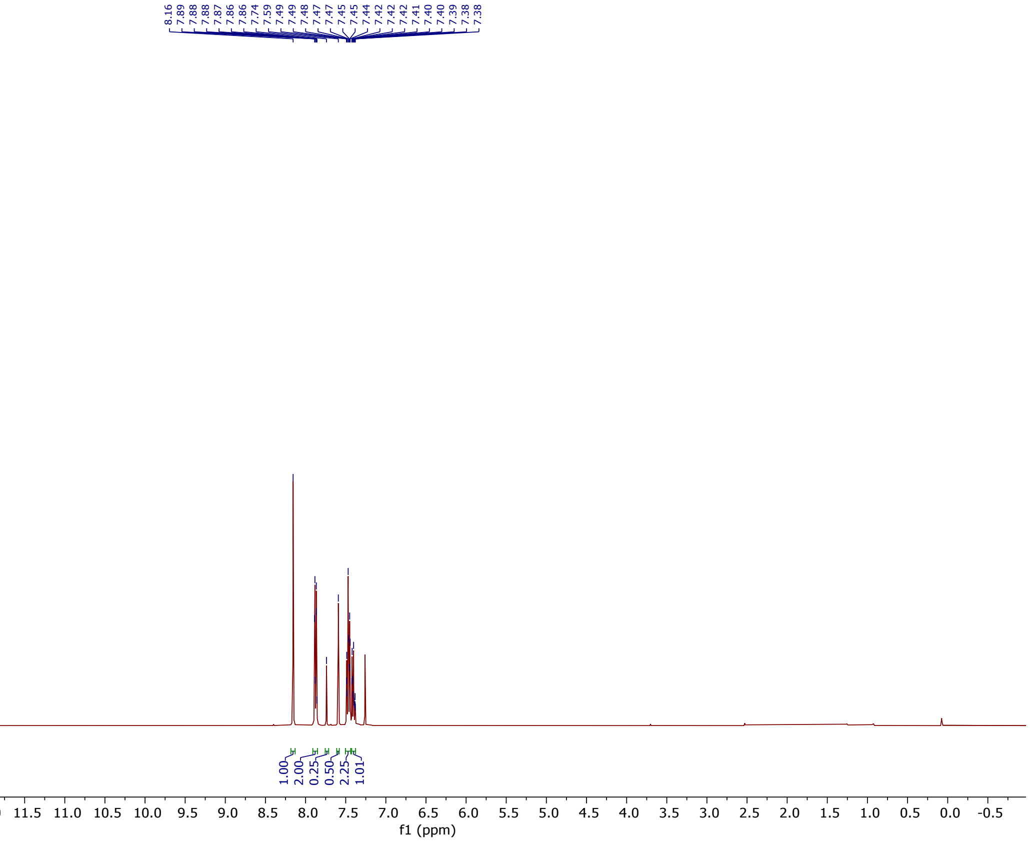

Figure S70. ^{1}H NMR spectrum of **7** ($CDCl_3$, 400 MHz)

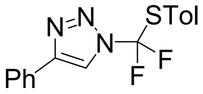

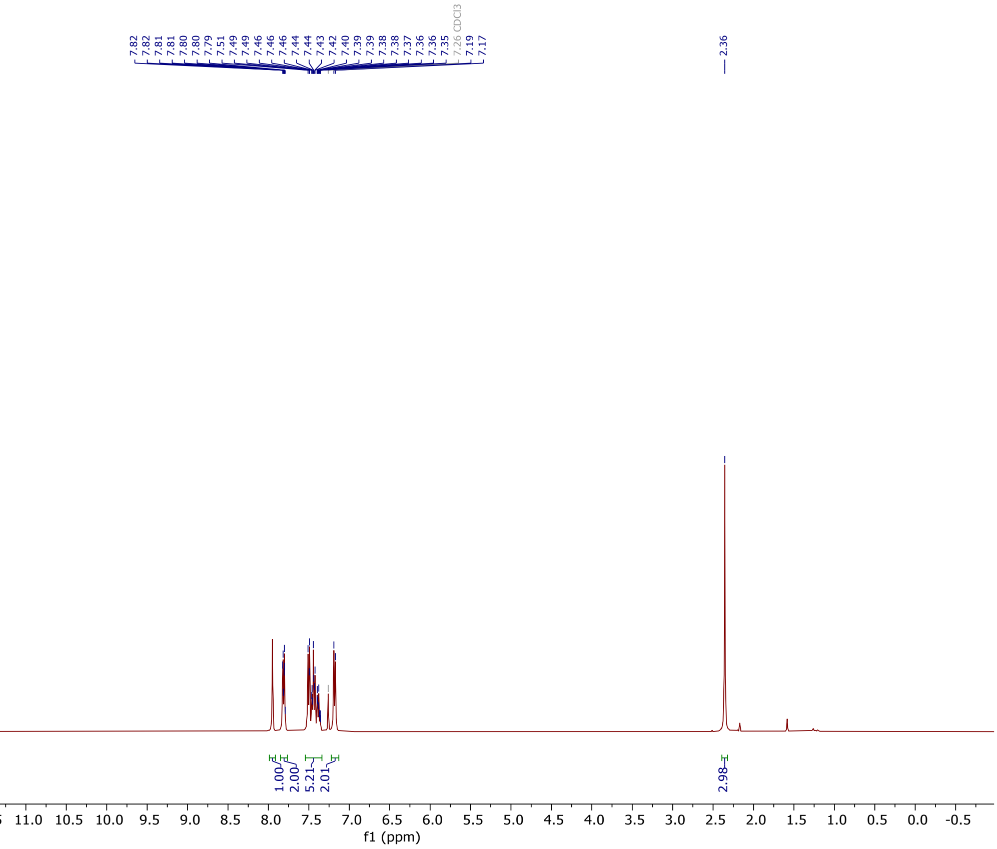

Figure S71. $^{13}C\{^{1}H\}$ NMR spectrum of **7** ($CDCl_3$, 101 MHz)

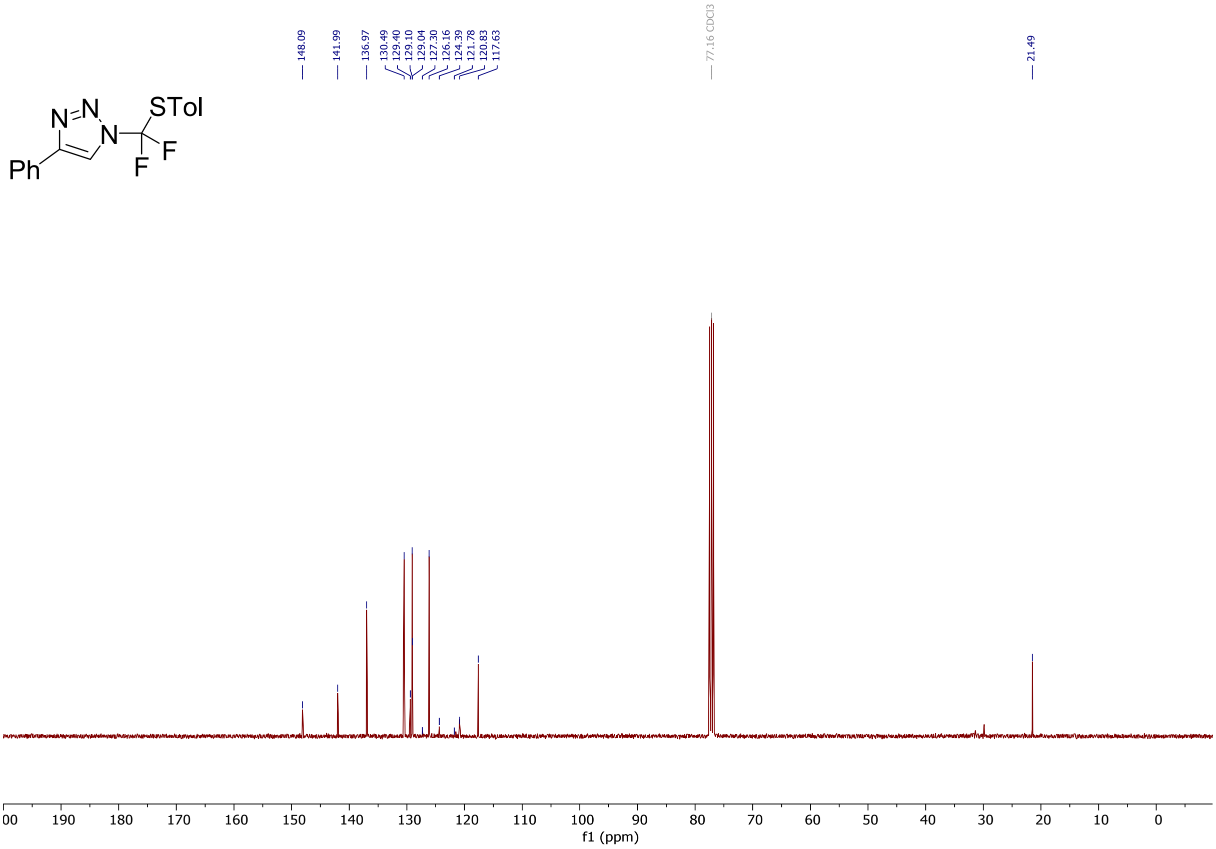

Figure S72. ^{19}F NMR spectrum of **7** ($CDCl_3$, 377 MHz)

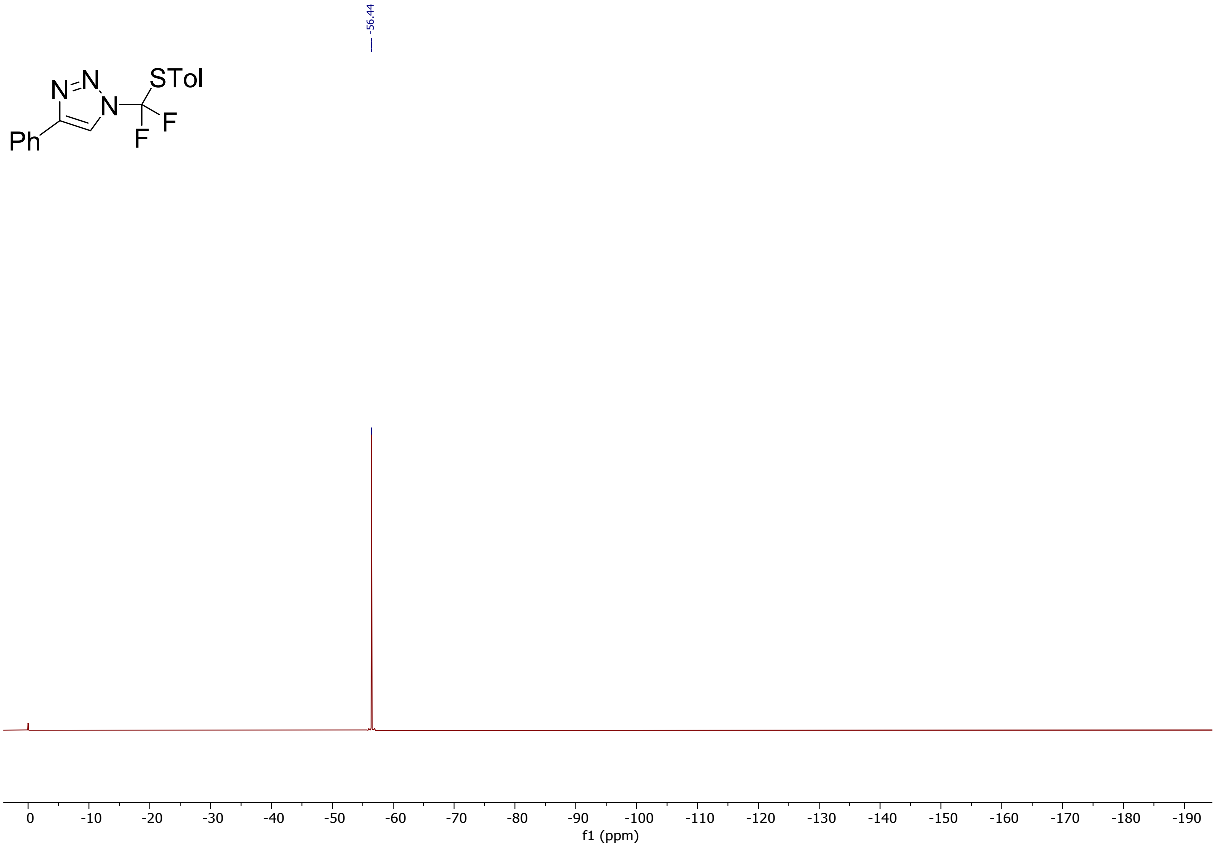

Figure S73. ^1H NMR spectrum of **8** (D_2O, 400 MHz)

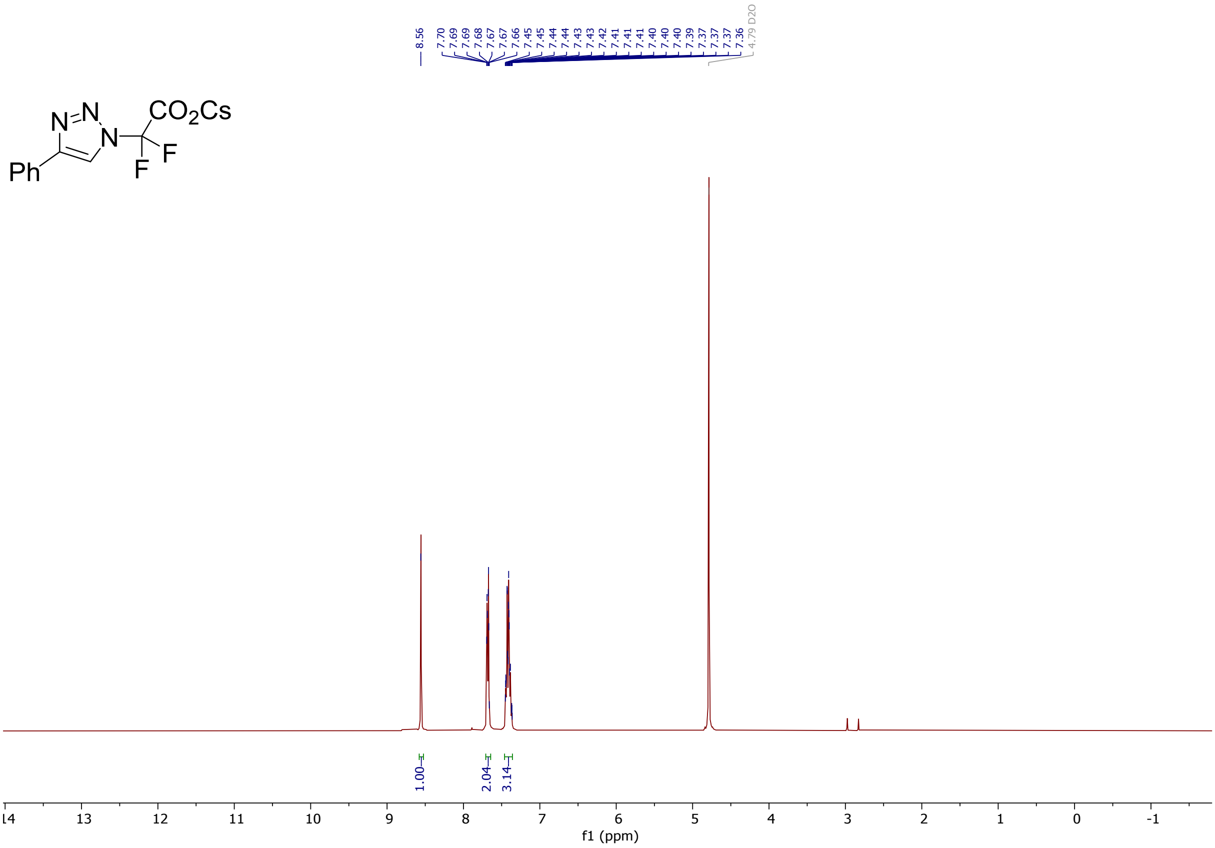

Figure S74. $^{13}C\{^1H\}$ NMR spectrum of **8** (D_2O, 101 MHz)

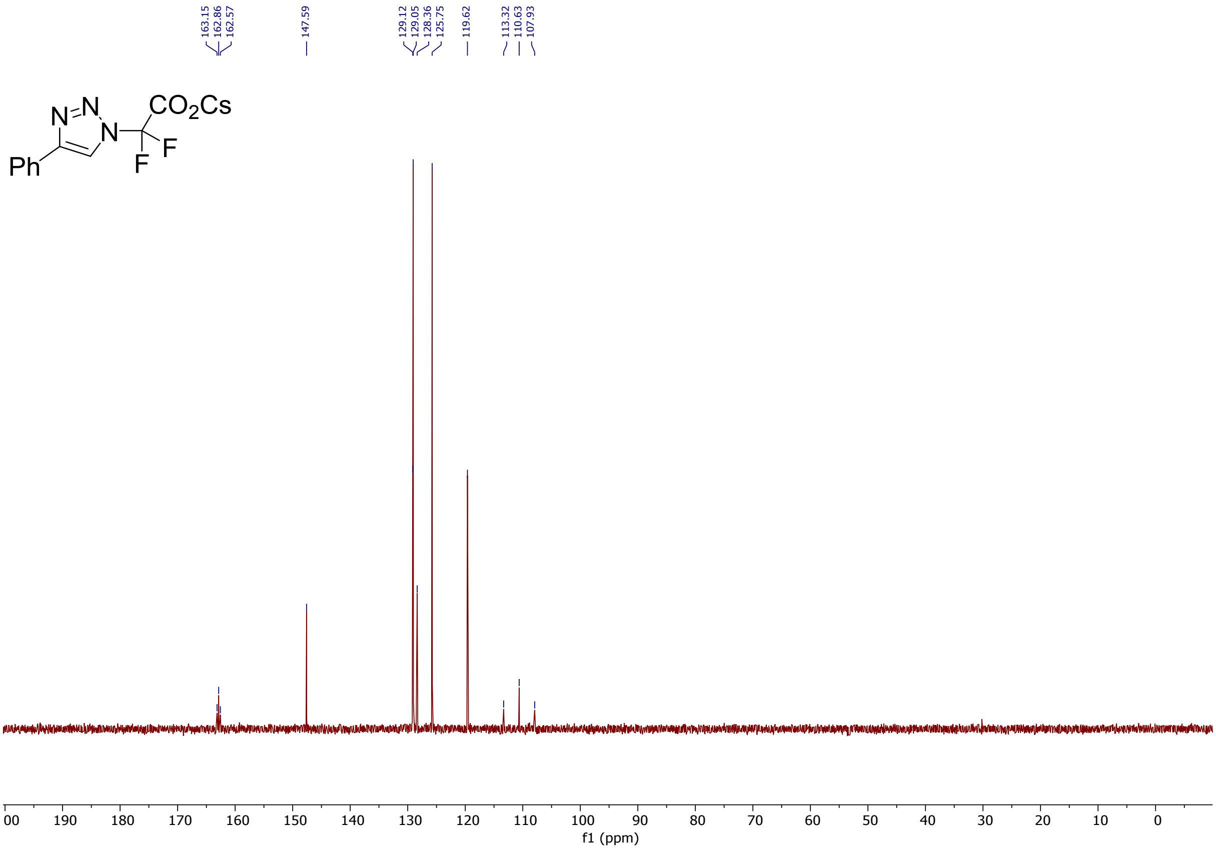

Figure S75. ^{19}F NMR spectrum of **8** (D_2O, 377 MHz)

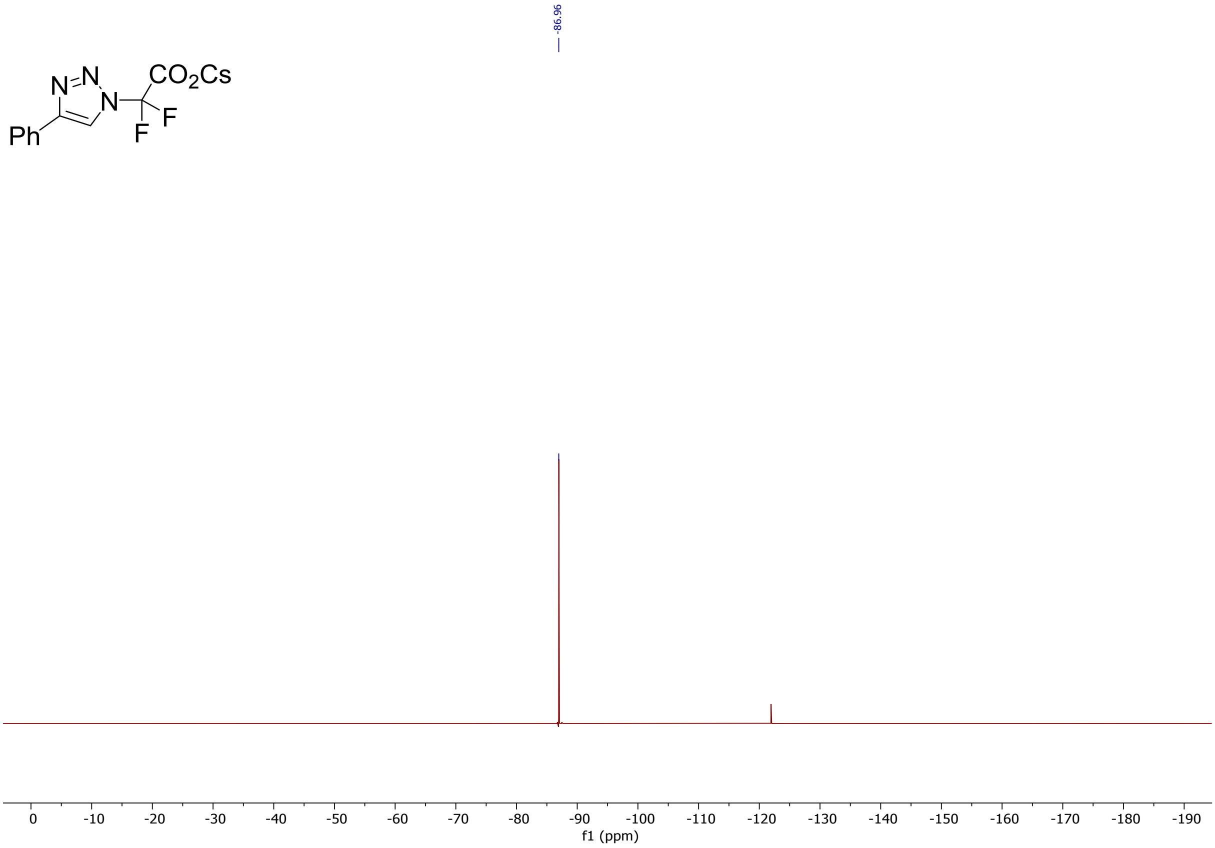

Figure S76. ^{1}H NMR spectrum of **9** (D_2O, 400 MHz)

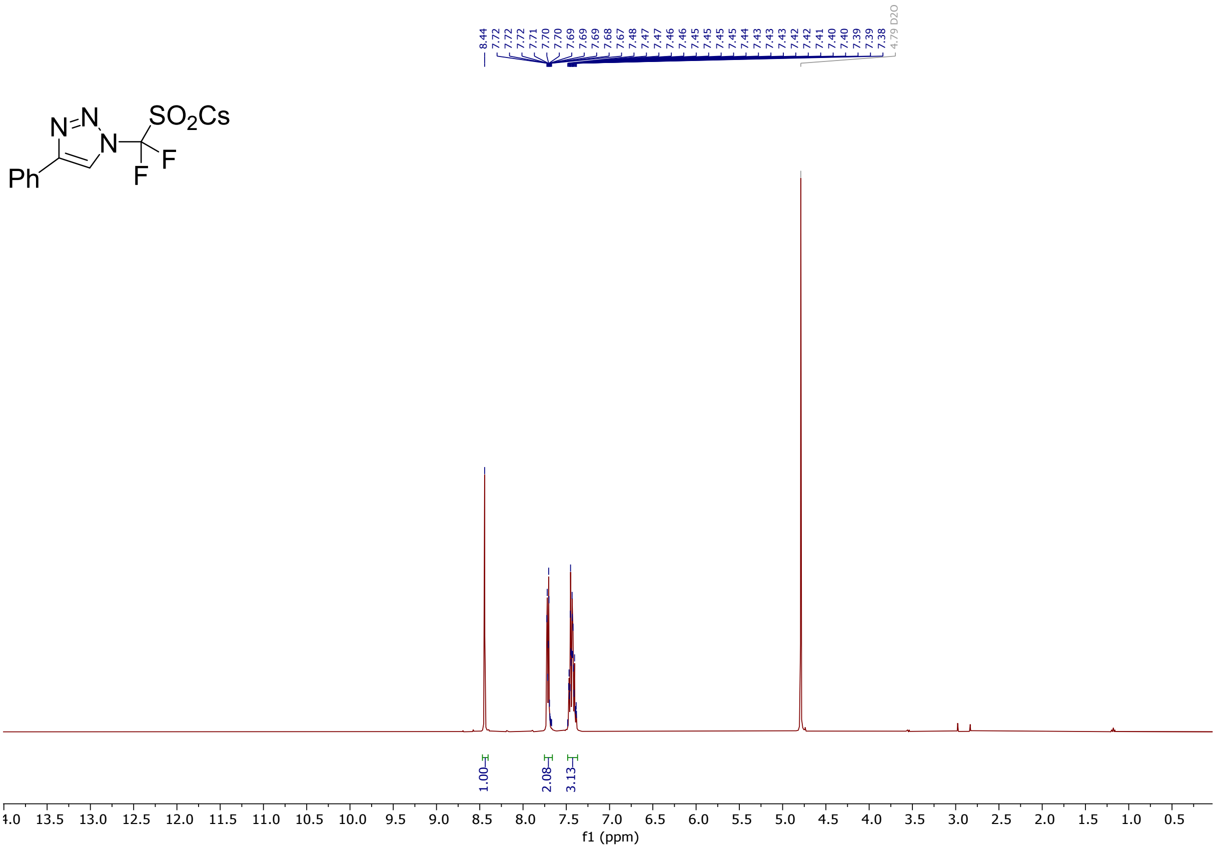

Figure S77. $^{13}C\{^1H\}$ NMR spectrum of **9** (D_2O, 101 MHz)

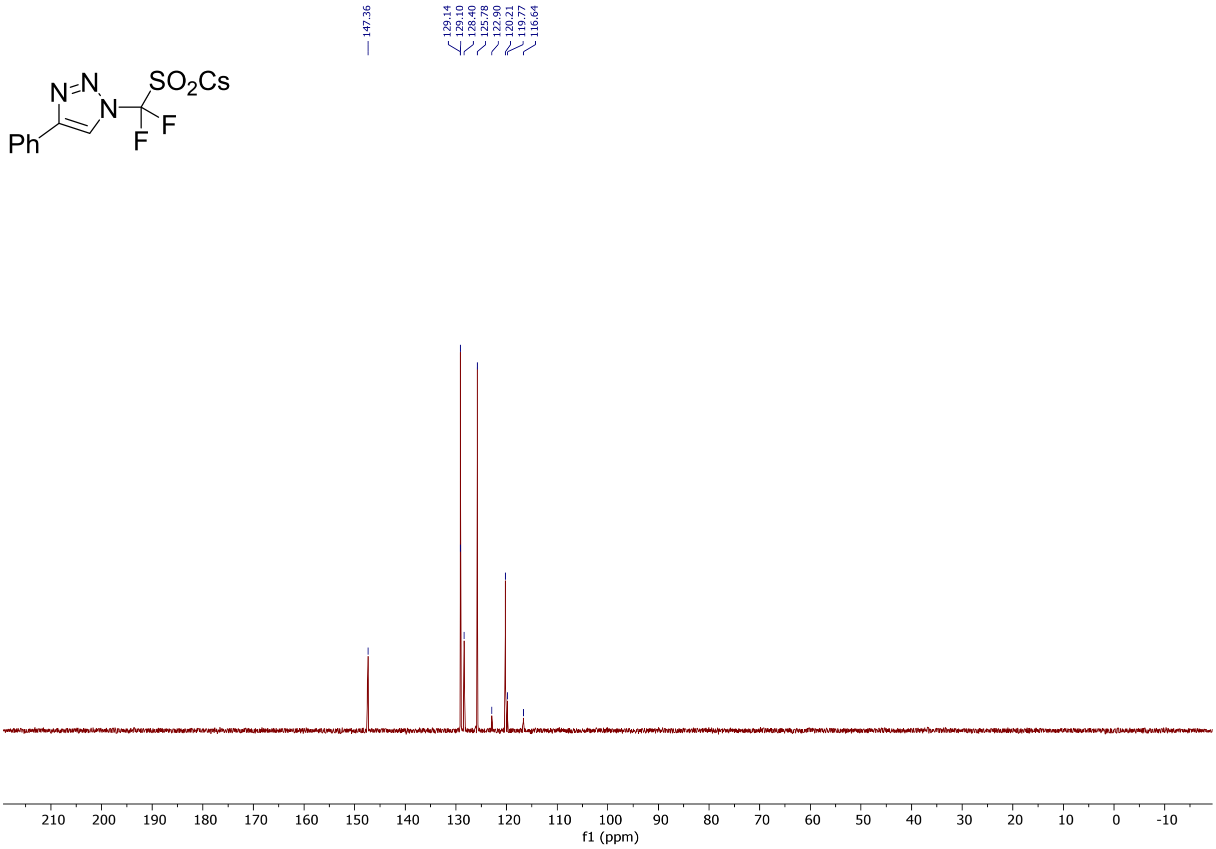

Figure S78. ^{19}F NMR spectrum of **9** (D_2O, 377 MHz)

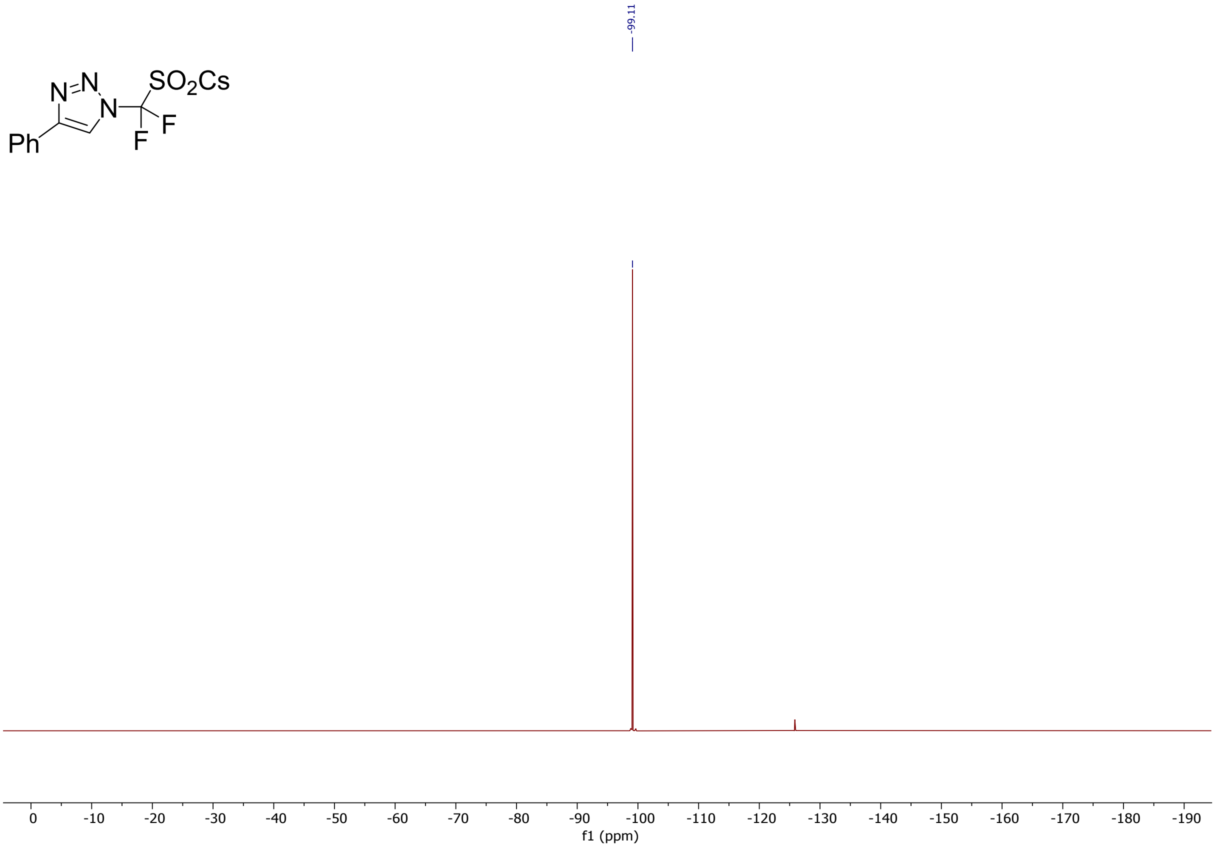

Figure S79. ^{1}H NMR spectrum of **10** ($CDCl_3$, 400 MHz)

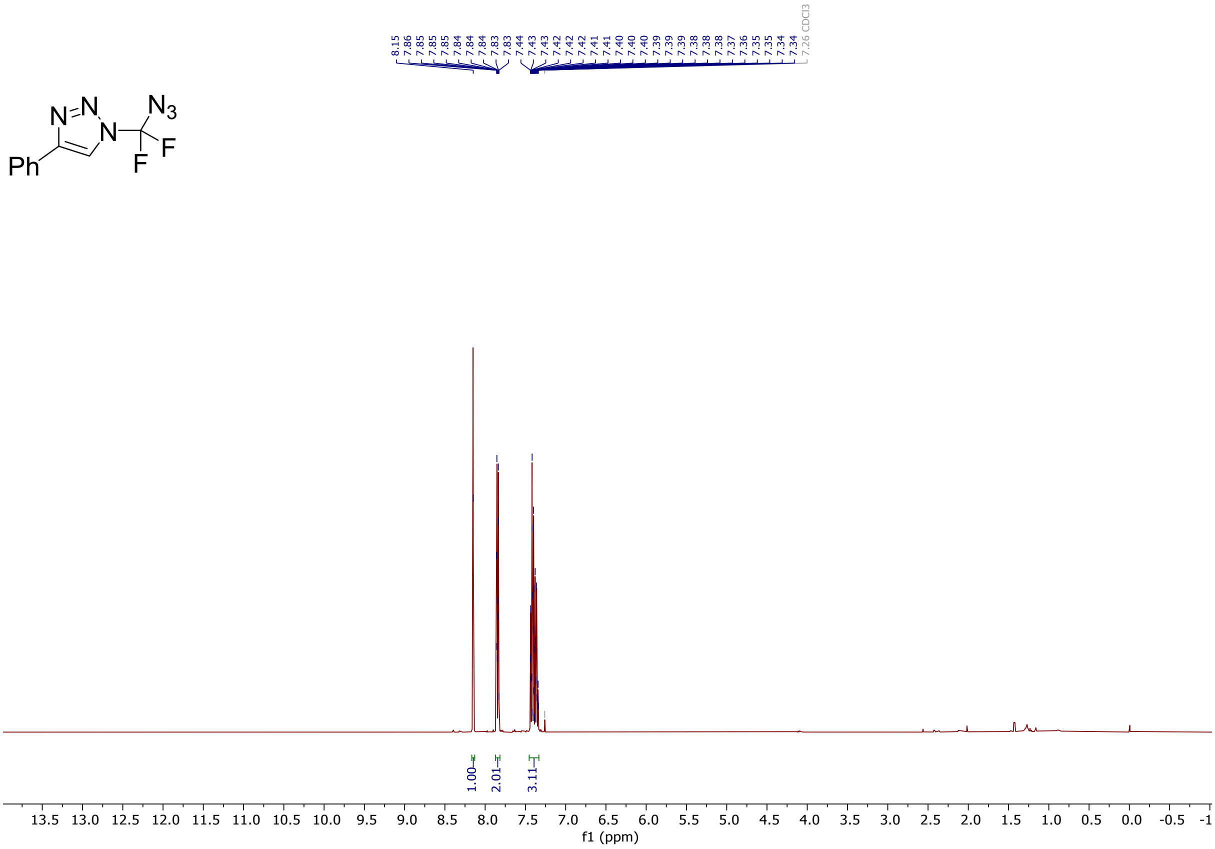

Figure S80. $^{13}C\{^{1}H\}$ NMR spectrum of **10** ($CDCl_3$, 101 MHz)

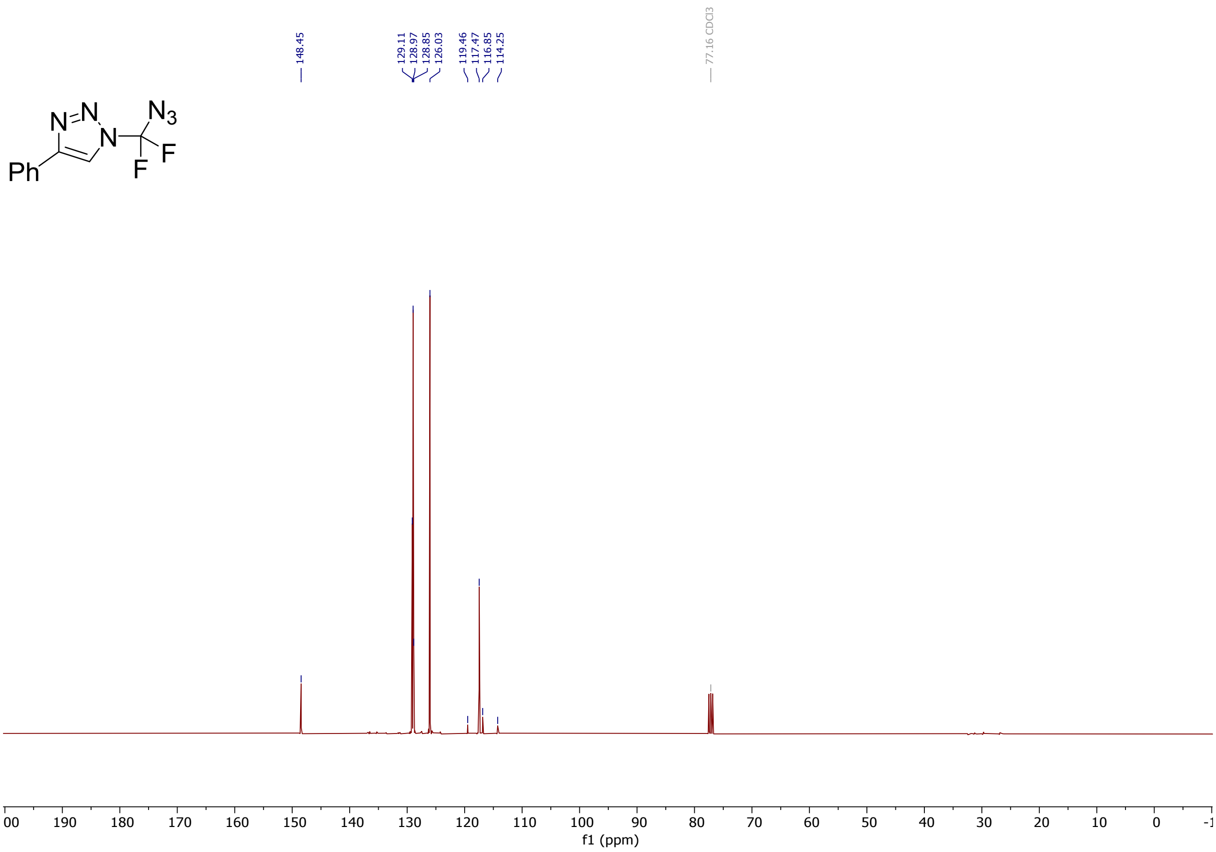

Figure S81. ^{19}F NMR spectrum of **10** ($CDCl_3$, 377 MHz)

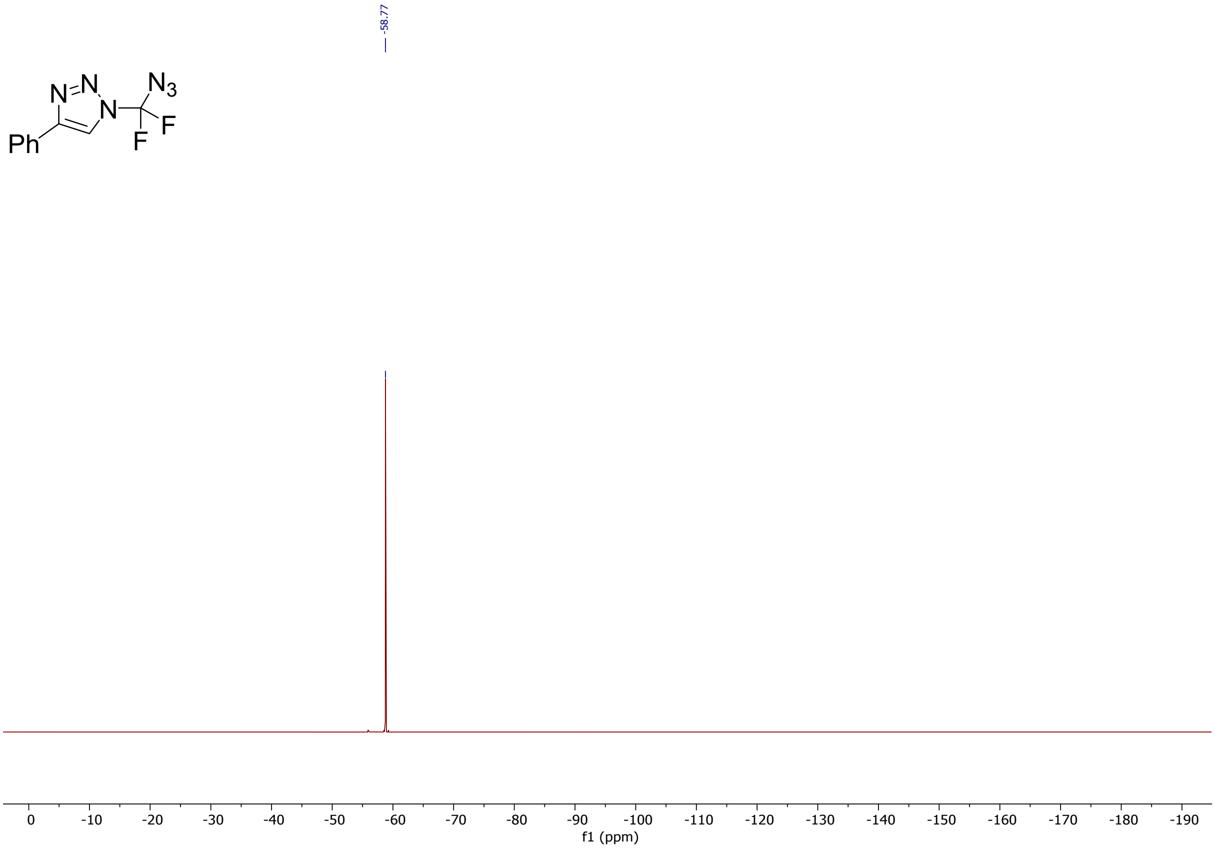

Figure S82. ^{1}H NMR spectrum of **11** ($CDCl_3$, 400 MHz)

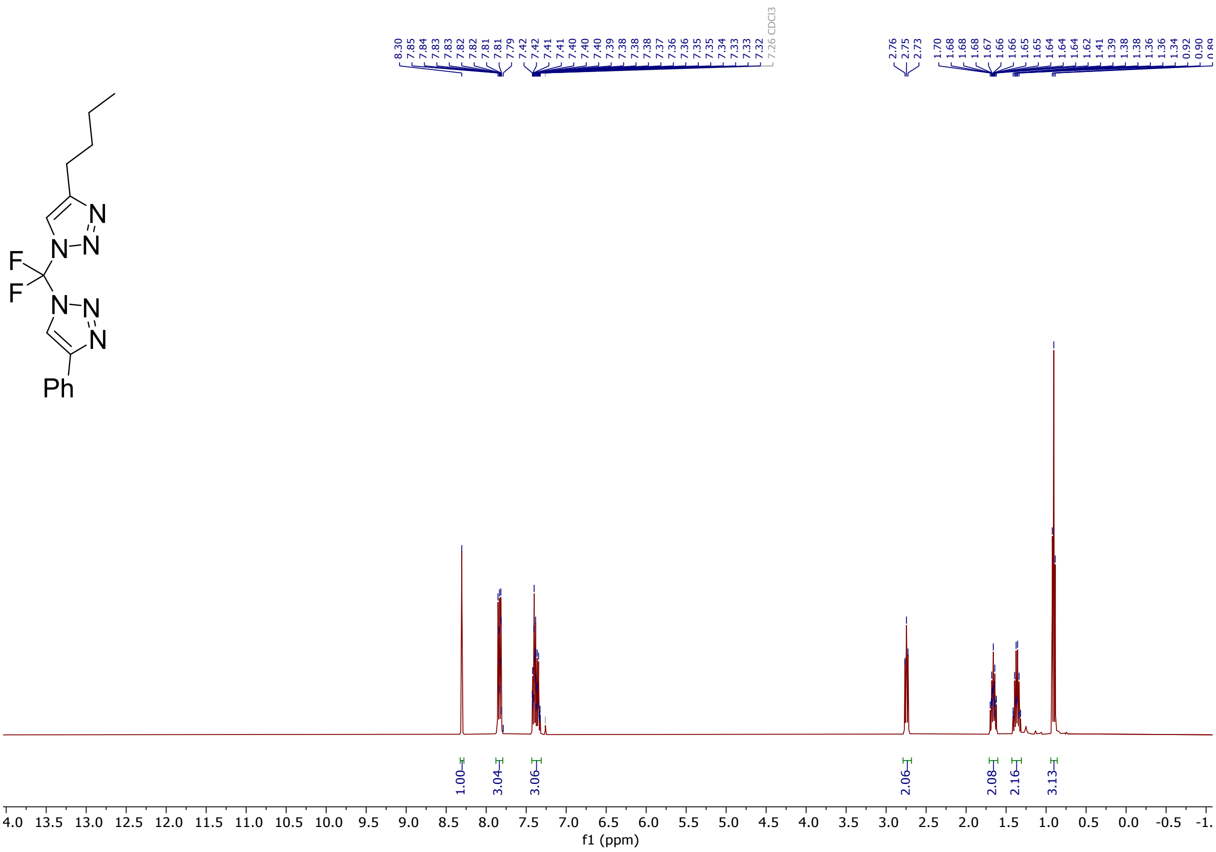

Figure S83. $^{13}C\{^1H\}$ NMR spectrum of **11** ($CDCl_3$, 101 MHz)

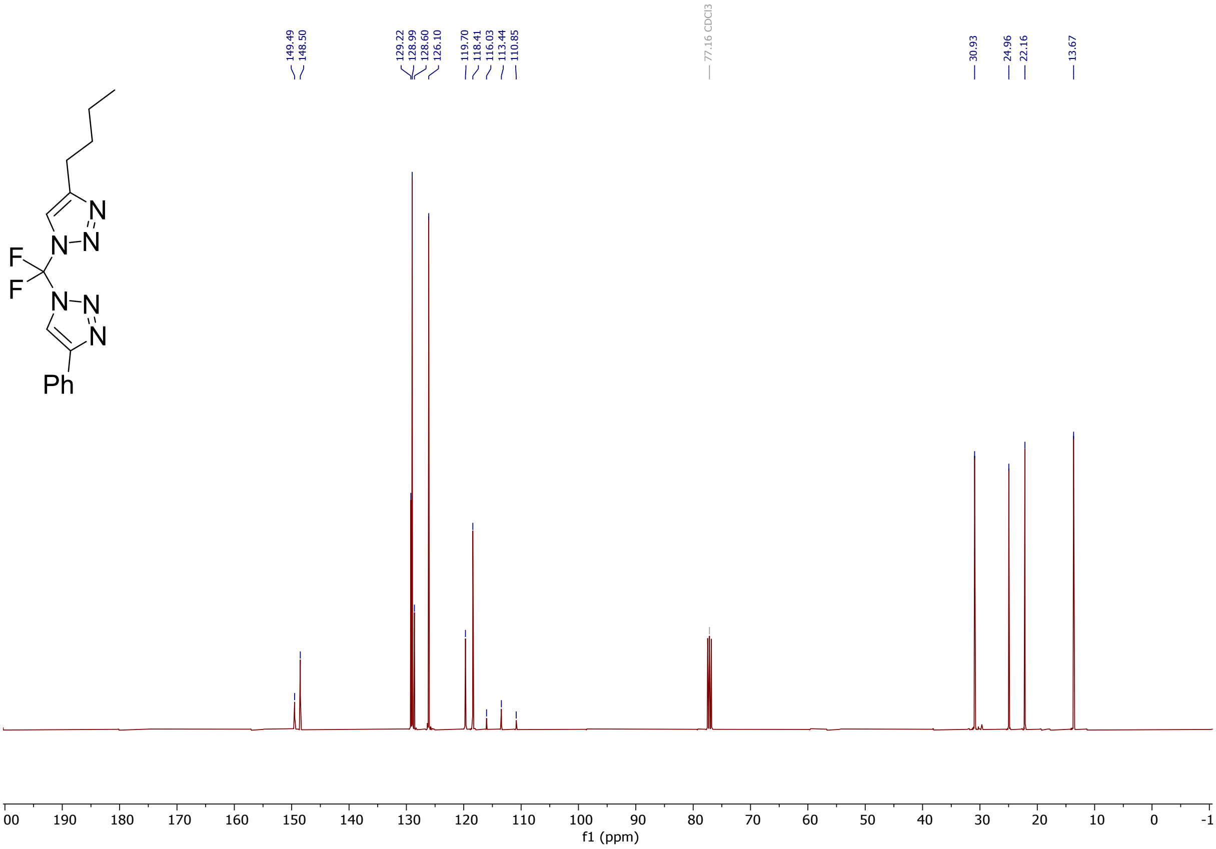

Figure S84. ^{19}F NMR spectrum of **11** ($CDCl_3$, 377 MHz)

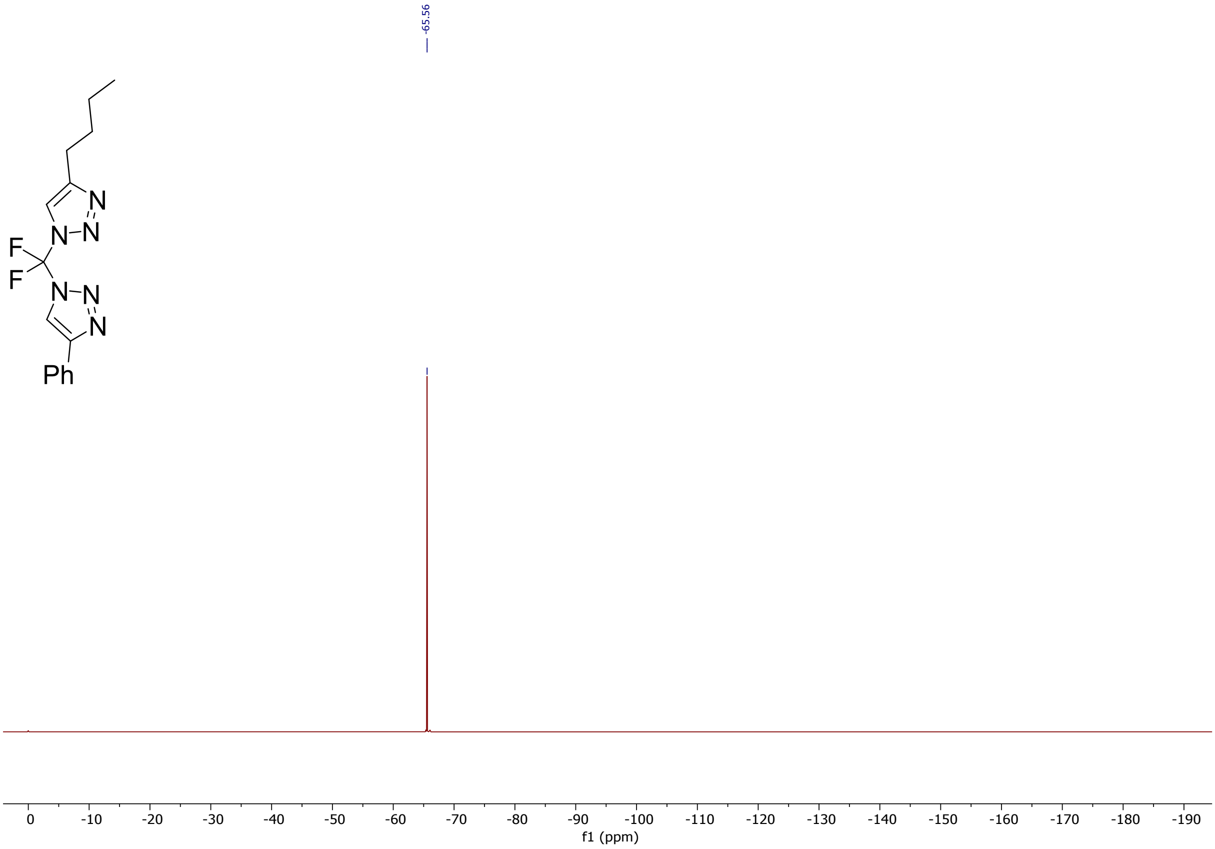

Figure S85. ^{1}H NMR spectrum of **12** (DMSO-D$_6$, 400 MHz)

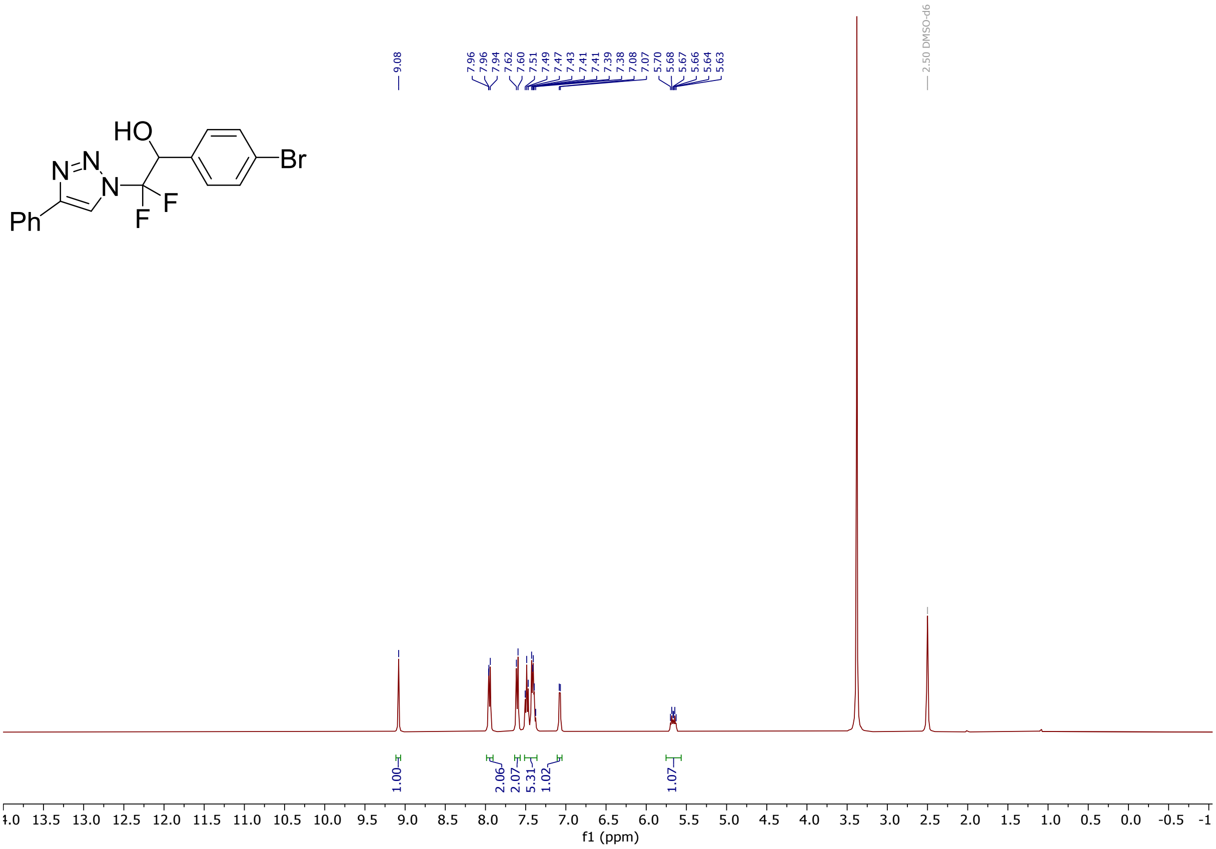

Figure S86. ^{13}C{^{1}H} NMR spectrum of **12** (DMSO-D$_6$, 101 MHz)

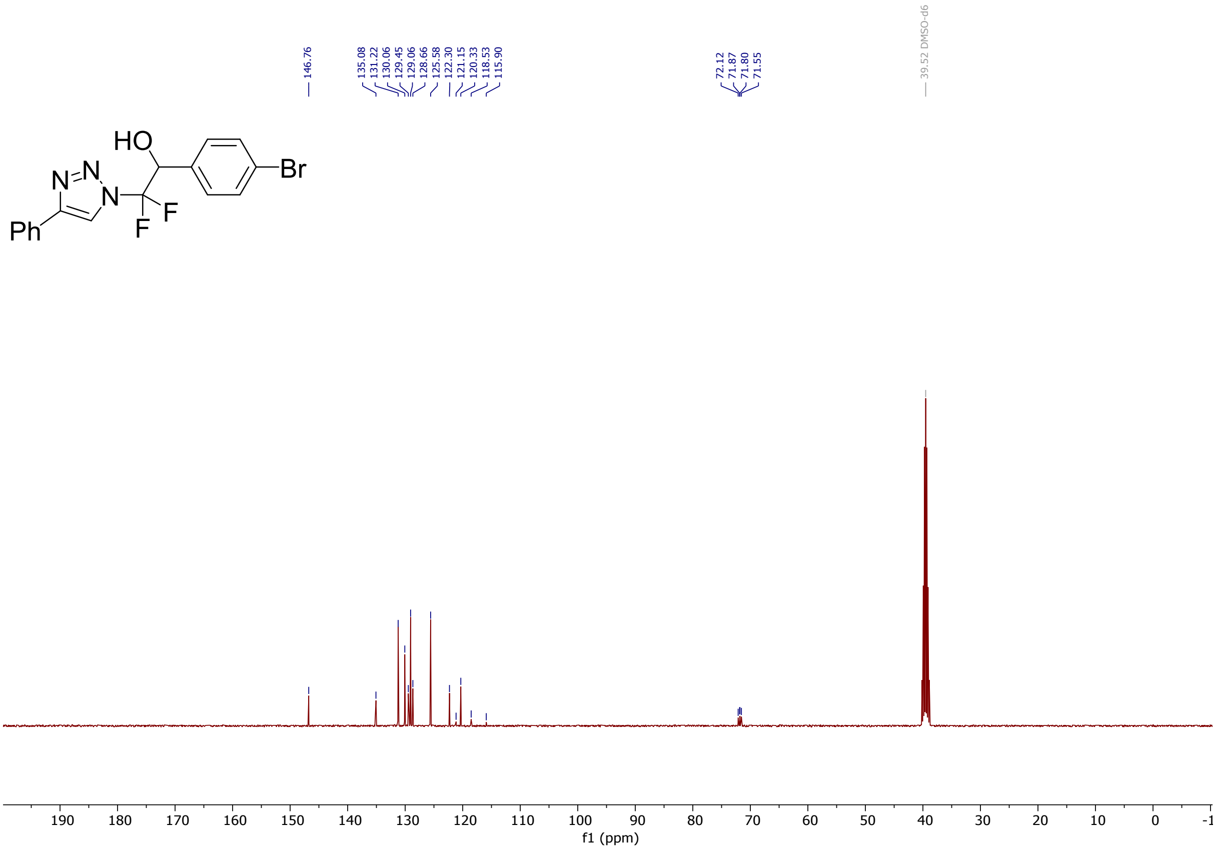

Figure S87. ^{19}F NMR spectrum of **12** (DMSO-D$_6$, 377 MHz)

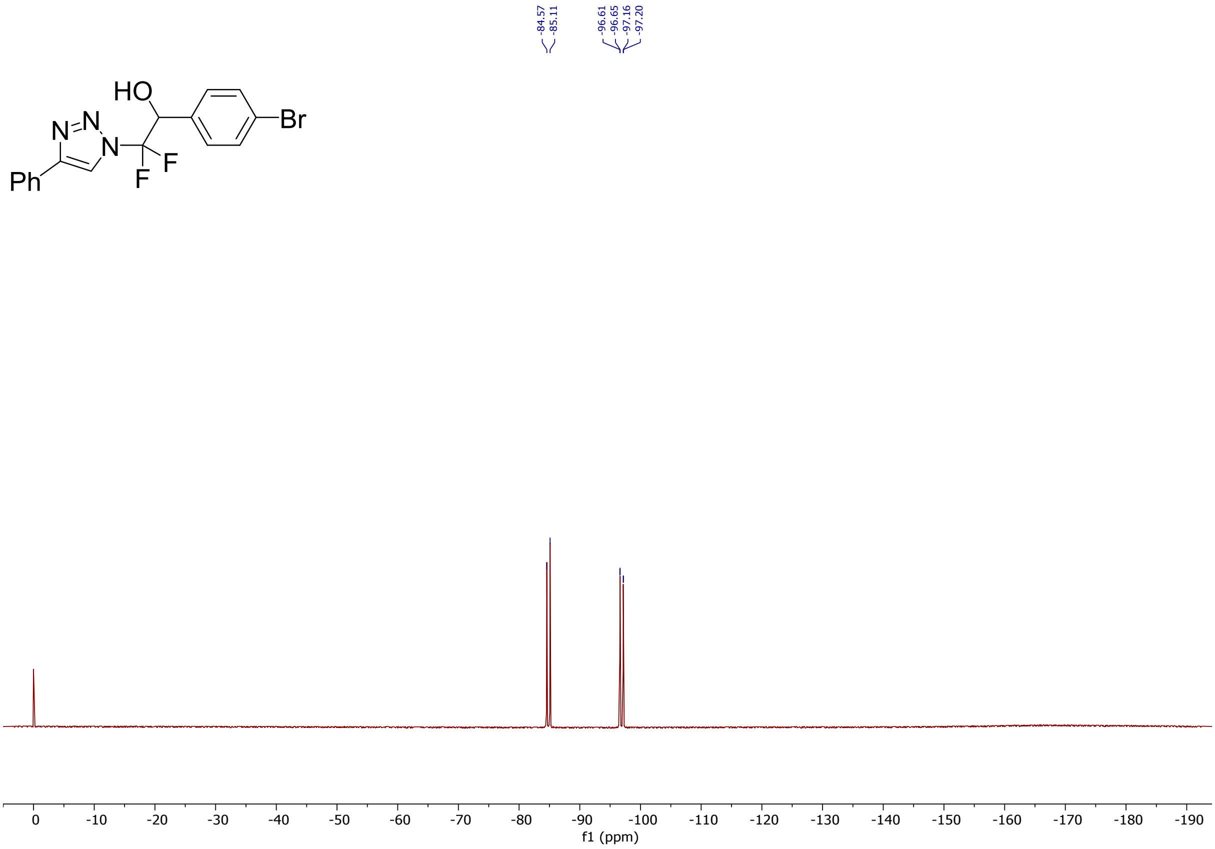

Figure S88. ^{1}H NMR spectrum of **13** ($CDCl_3$, 400 MHz)

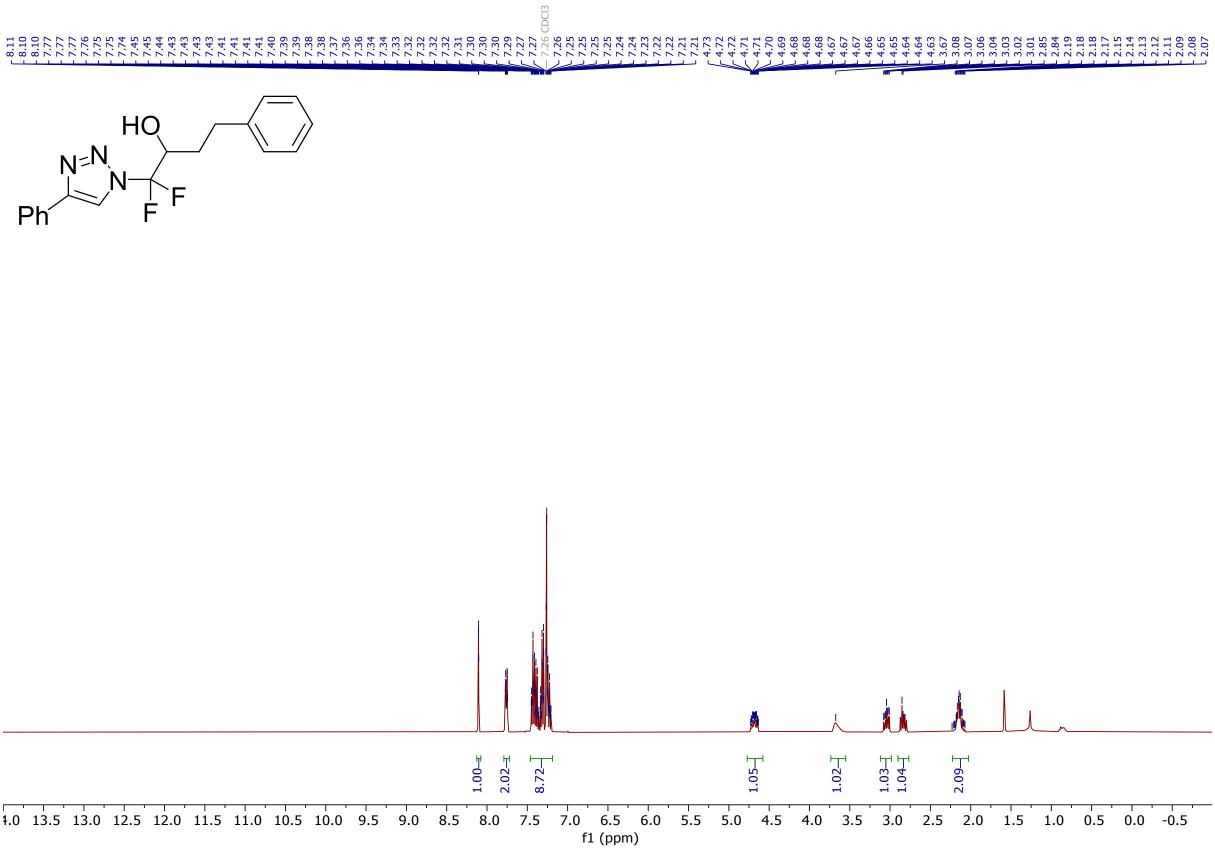

Figure S89. $^{13}C\{^{1}H\}$ NMR spectrum of **13** ($CDCl_3$, 101 MHz)

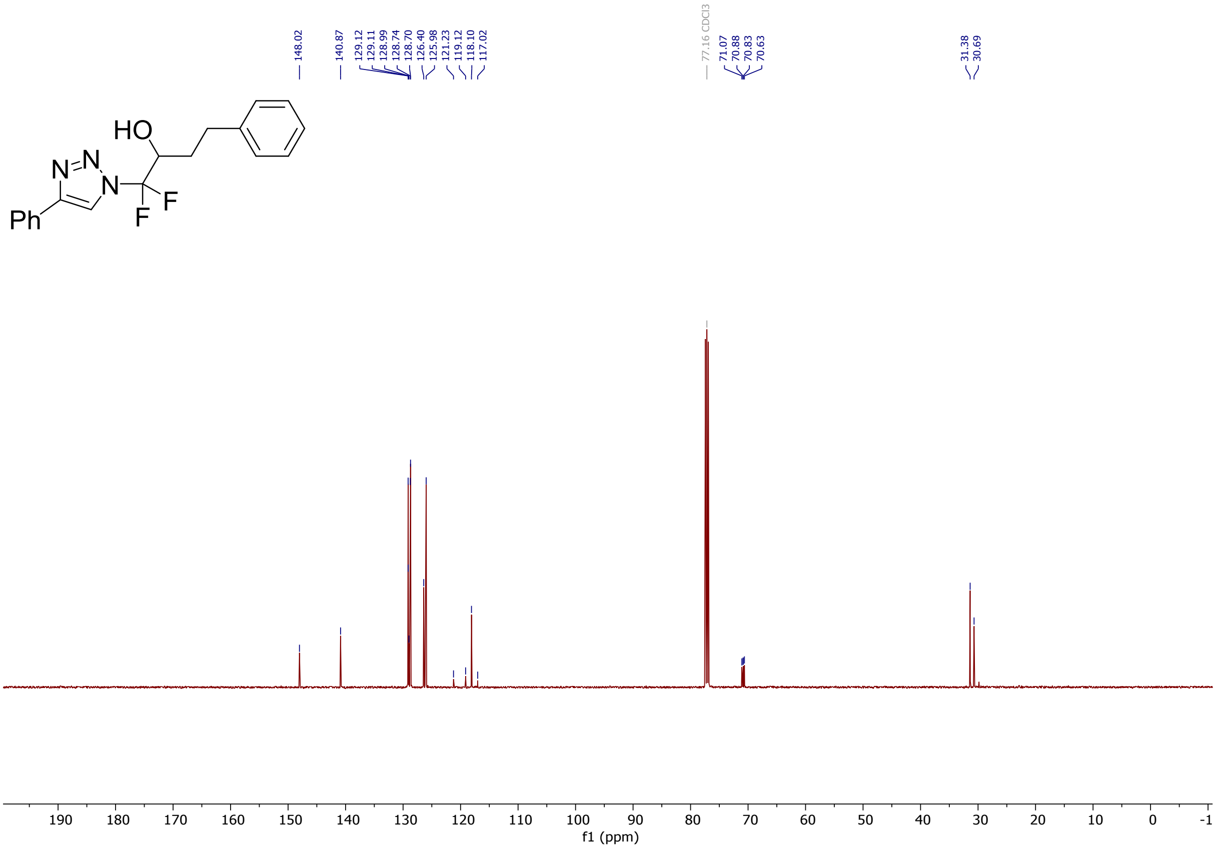

Figure S90. ^{19}F NMR spectrum of **13** ($CDCl_3$, 377 MHz)

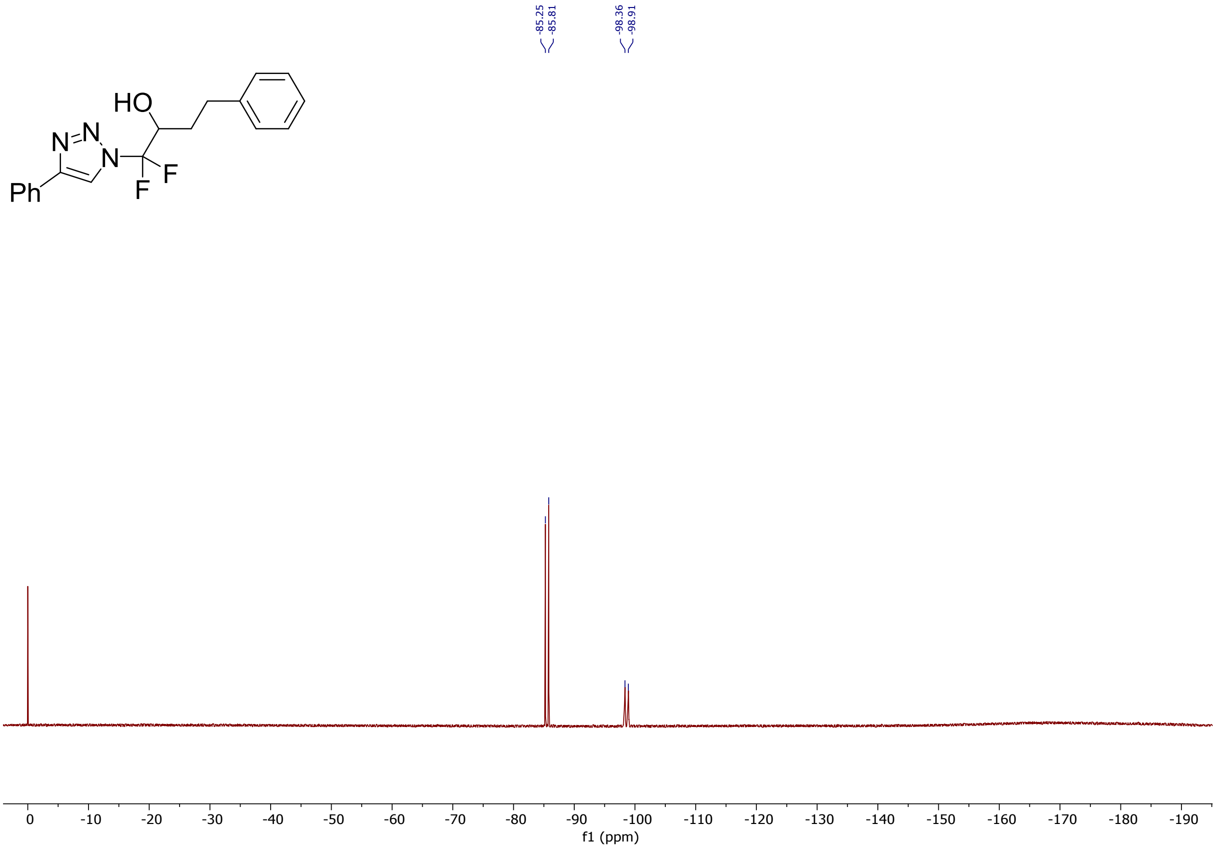

Figure S91. ^{1}H NMR spectrum of **14** ($CDCl_3$, 400 MHz)

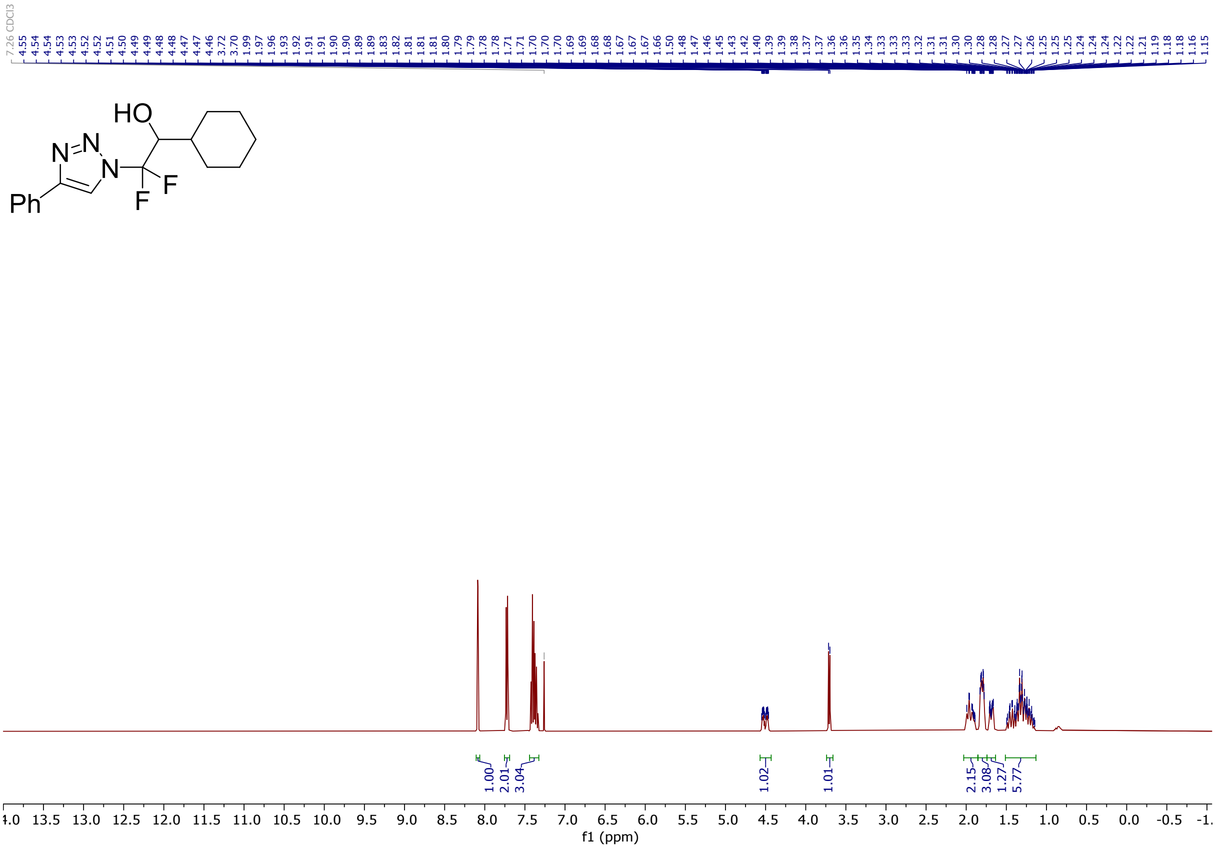

Figure S92. ^{13}C{^{1}H} NMR spectrum of **14** ($CDCl_3$, 101 MHz)

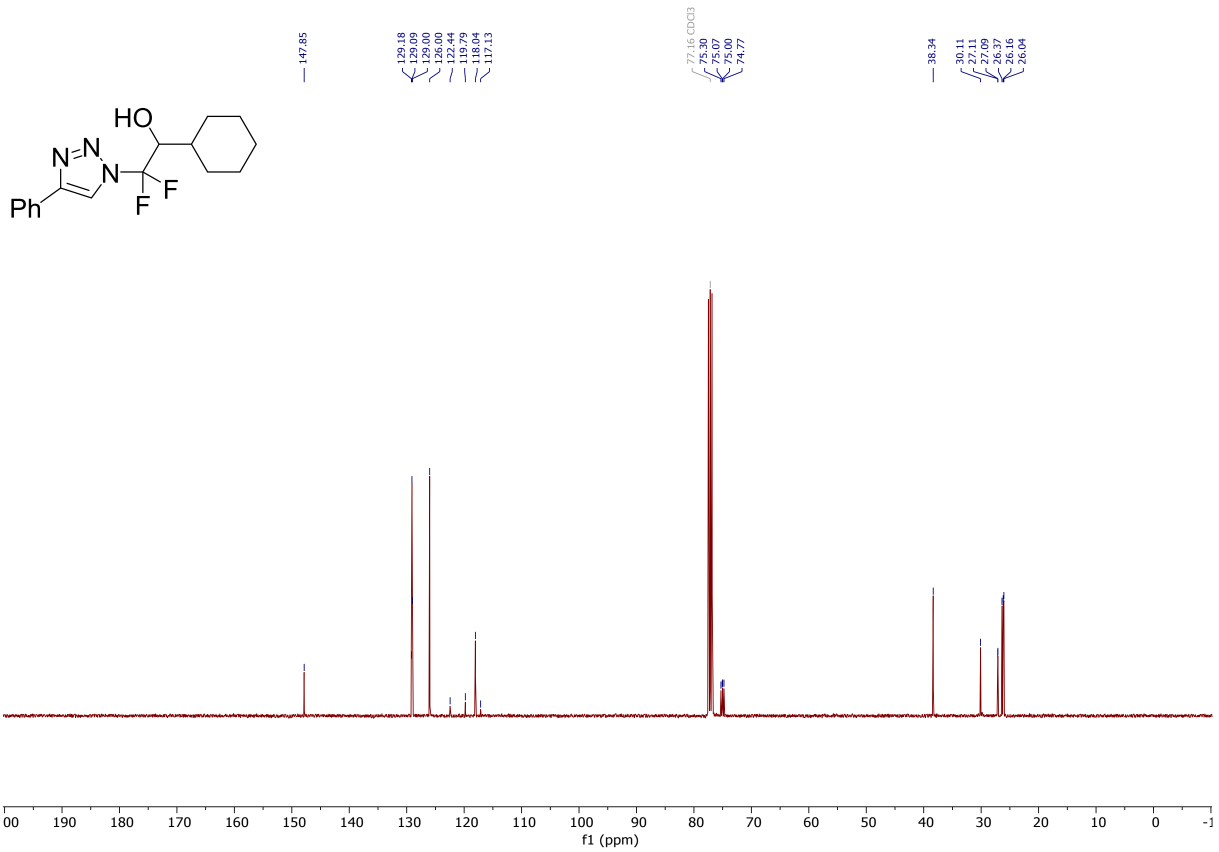

Figure S93. ^{19}F NMR spectrum of **14** ($CDCl_3$, 377 MHz)

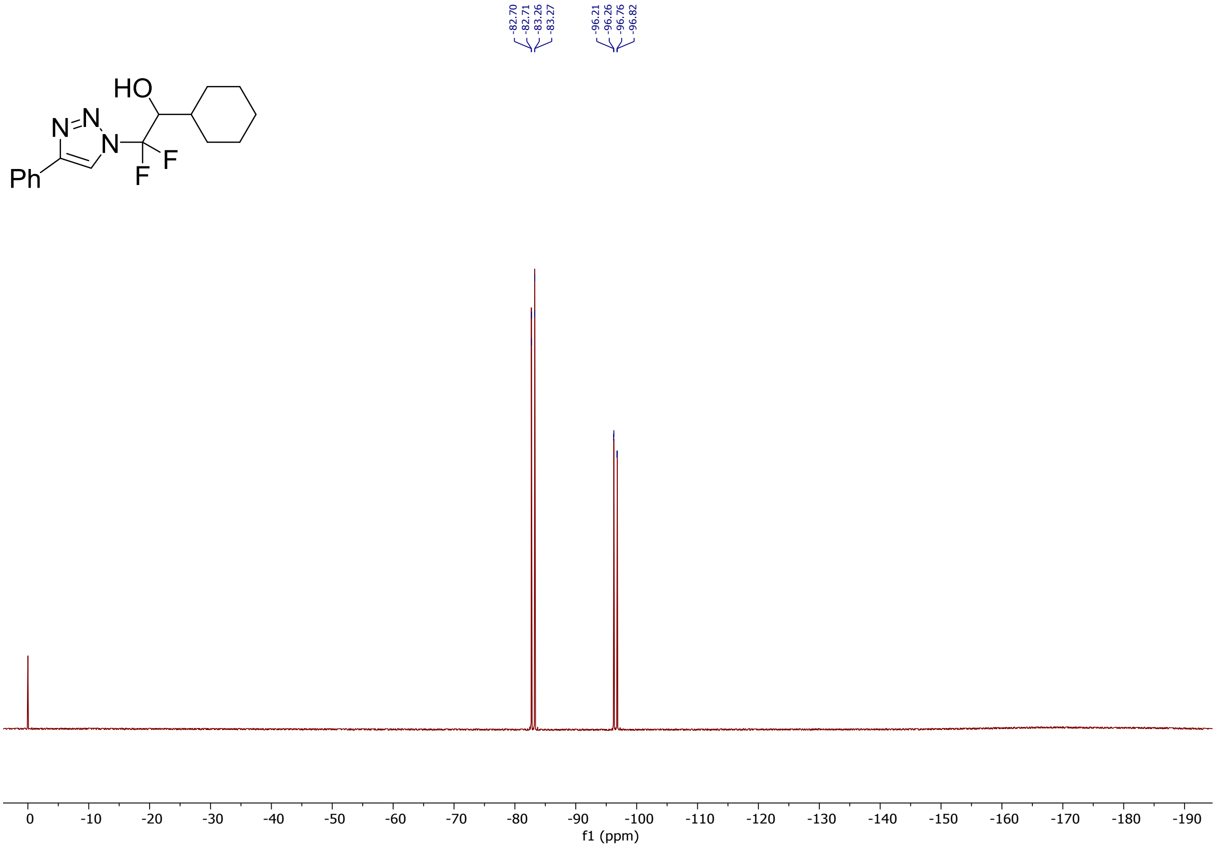

Figure S94. ^{1}H NMR spectrum of **15** ($CDCl_3$, 400 MHz)

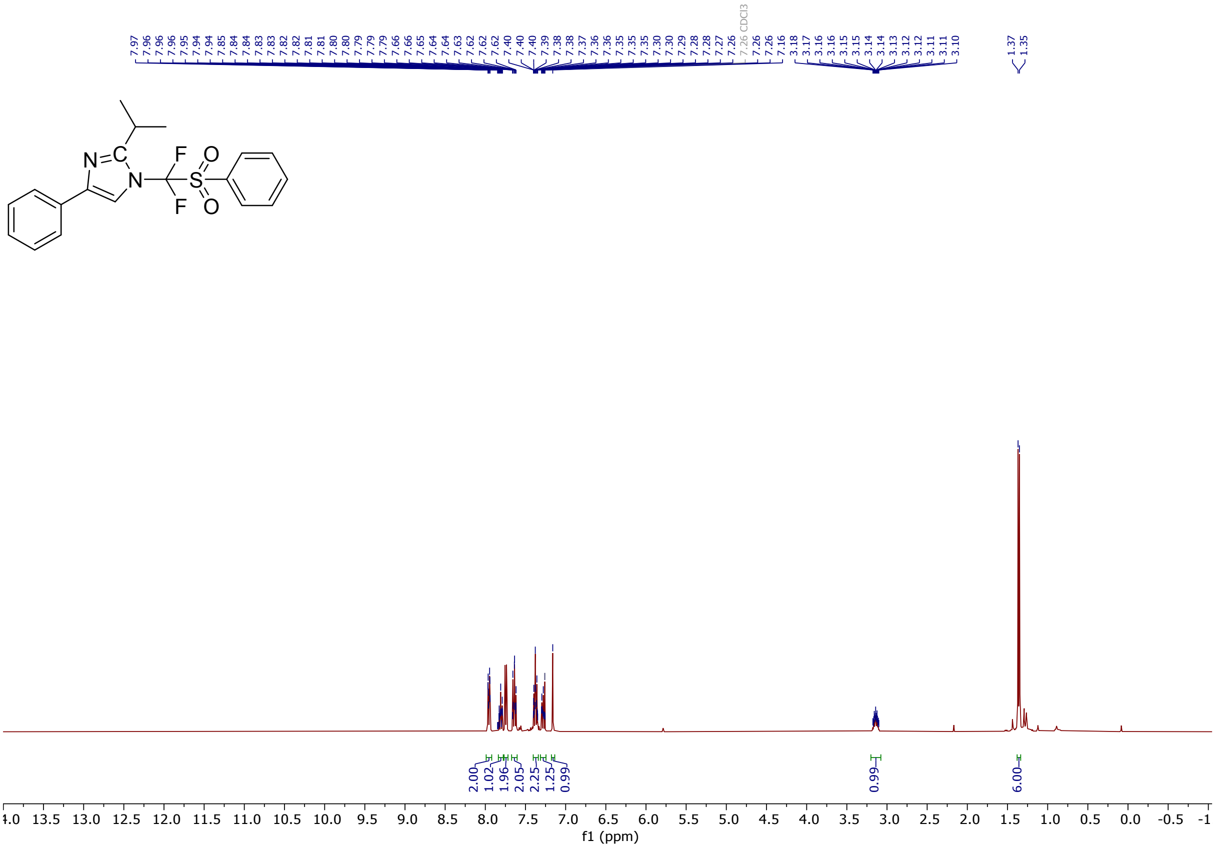

Figure S95. $^{13}C\{^{1}H\}$ NMR spectrum of **15** ($CDCl_3$, 101 MHz)

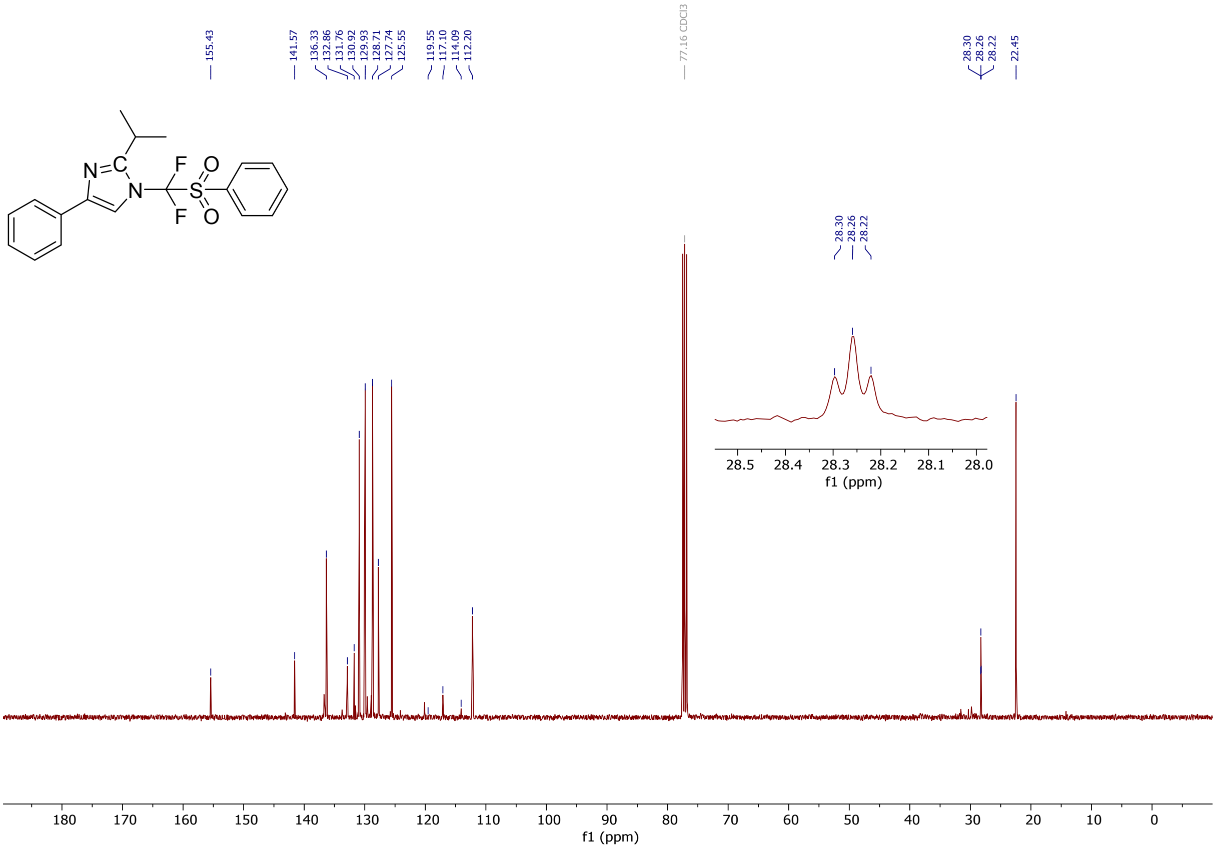

Figure S96. ^{19}F NMR spectrum of **15** ($CDCl_3$, 377 MHz)

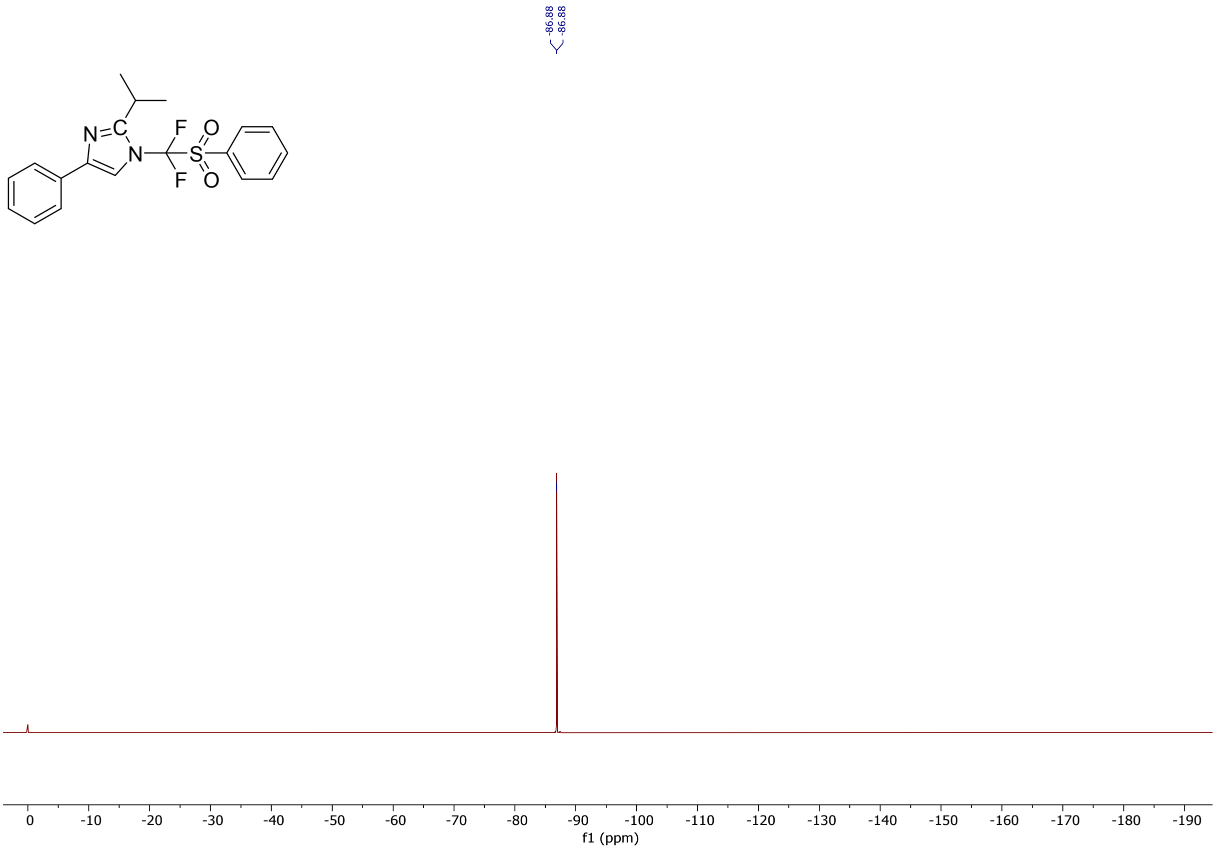

Figure S97. ^{1}H NMR spectrum of **16** ($CDCl_3$, 400 MHz)

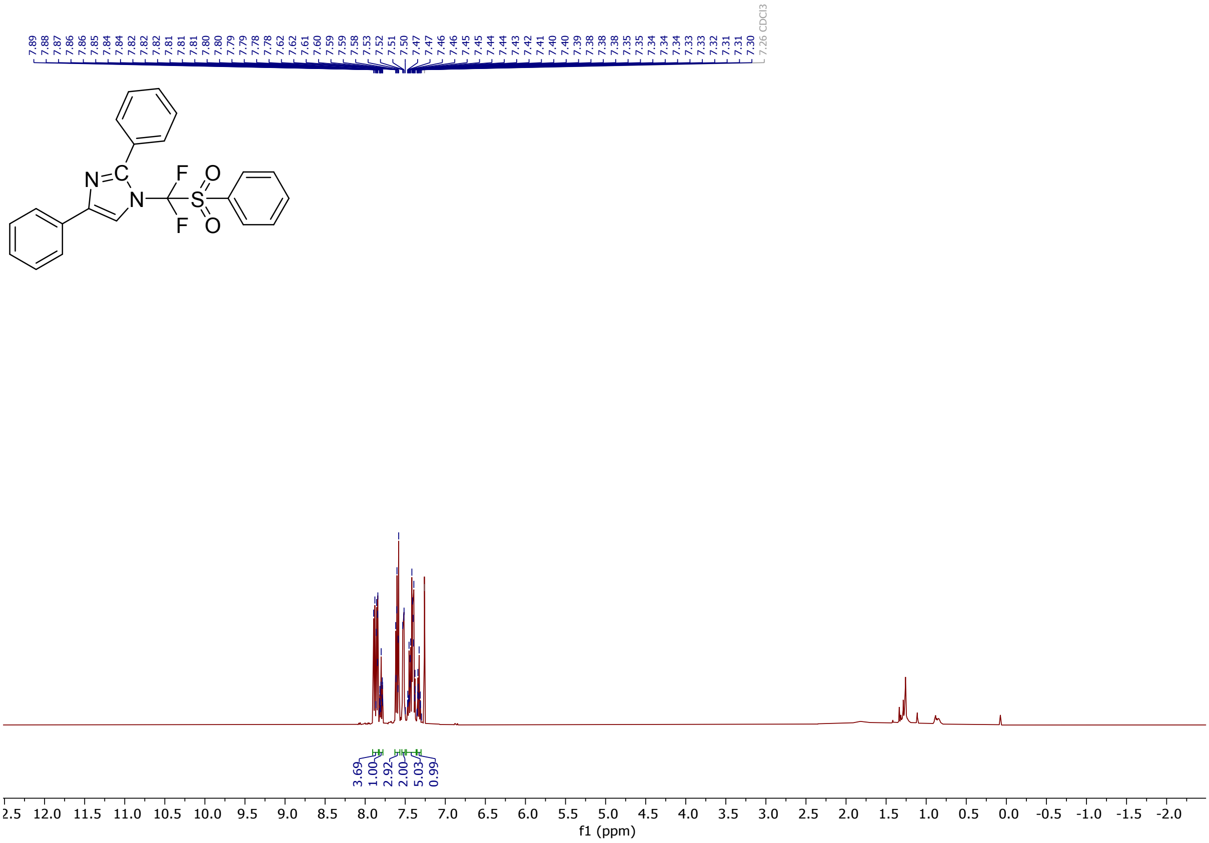

Figure S98. $^{13}C\{^{1}H\}$ NMR spectrum of **16** ($CDCl_3$, 101 MHz)

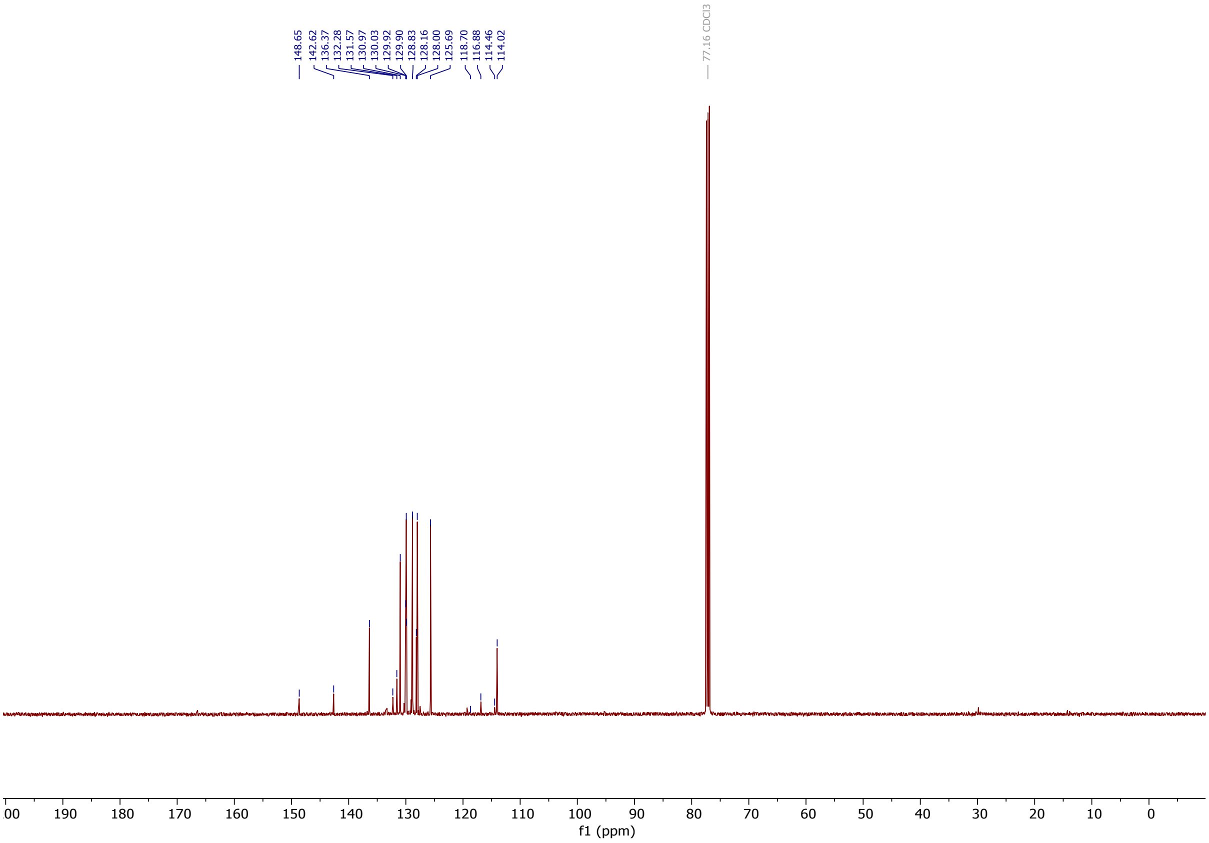

Figure S99. ^{19}F NMR spectrum of **16** ($CDCl_3$, 377 MHz)

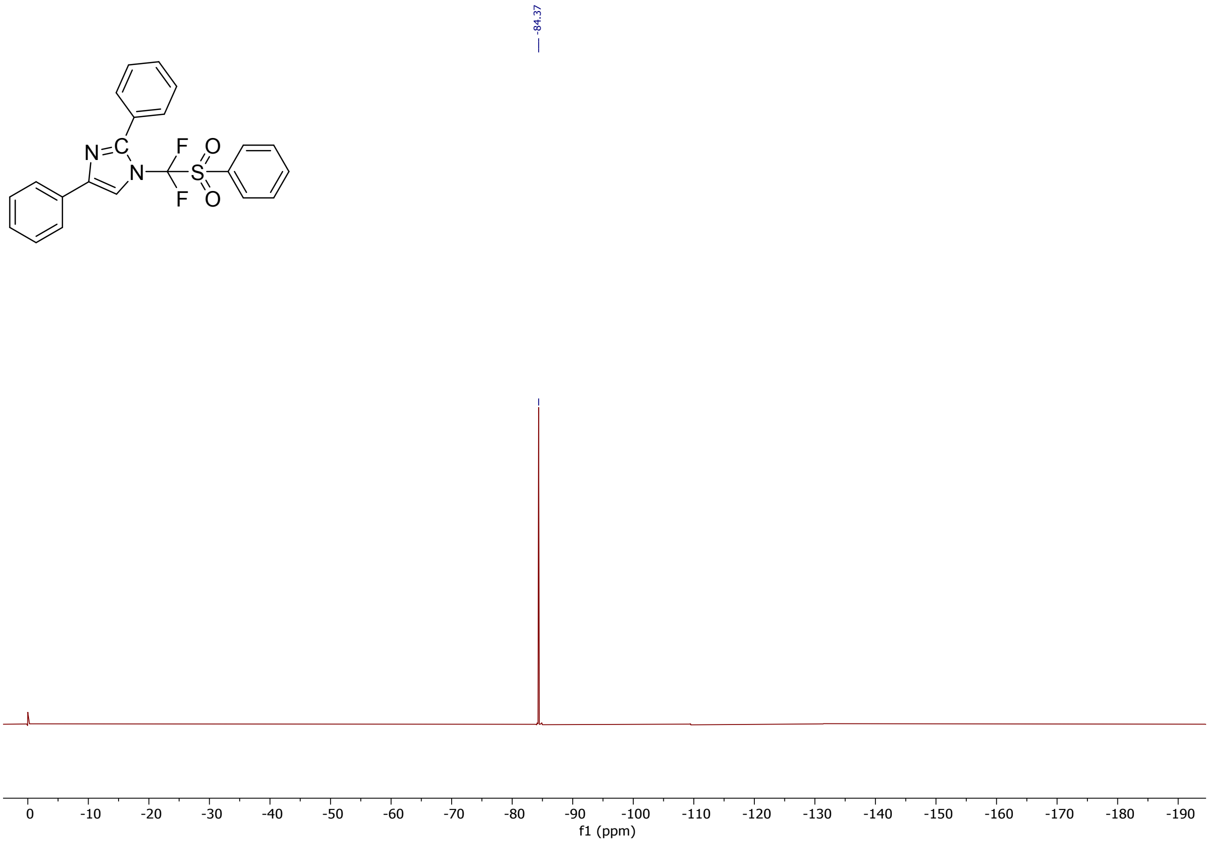

References

1 SAINT. Bruker AXS Inc., Madison, Wisconsin, USA, 2015.

2 Altomare, A.; Cascarano, G.; Giacovazzo G.; Guagliardi A.; Burla M. C.; Polidori, G.; Camalli, M. *J. Appl. Cryst.* **1994,** *27*, 435.

3 Betteridge, P. W.; Carruthers, J. R.; Cooper, R. I.; Prout, K., Watkin, D. J. *J. Appl. Cryst.* **2003**, *36*, 1487.
